# Selfing mutants link Ku proteins to mating type determination in *Tetrahymena*

**DOI:** 10.1371/journal.pbio.3000756

**Published:** 2020-08-03

**Authors:** I-Ting Lin, Meng-Chao Yao

**Affiliations:** Institute of Molecular Biology, Academia Sinica, Nankang, Taipei, Taiwan; Duke University Medical Center, UNITED STATES

## Abstract

Recognition of self and nonself is important for outcrossing organisms, and different mating types establish the barrier against self-mating. In the unicellular ciliate *T*. *thermophila*, mating type determination requires complex DNA rearrangements at a single *mat* locus during conjugation to produce a type-specific gene pair (*MTA* and *MTB*) for 1 of 7 possible mating types. Surprisingly, we found that decreased expression of the DNA breakage-repair protein Ku80 at late stages of conjugation generated persistent selfing phenotype in the progeny. DNA analysis revealed multiple mating-type gene pairs as well as a variety of mis-paired, unusually arranged mating-type genes in these selfers that resemble some proposed rearrangement intermediates. They are found also in normal cells during conjugation and are lost after 10 fissions but are retained in Ku mutants. Silencing of *TKU80* or *TKU70-2* immediately after conjugation also generated selfing phenotype, revealing a hidden DNA rearrangement process beyond conjugation. Mating reactions between the mutant and normal cells suggest a 2-component system for self–nonself-recognition through *MTA* and *MTB* genes.

## Abstract 2

In the unicellular ciliate Tetrahymena thermophila, mating type determination requires complex DNA rearrangements to produce a type-specific MAT gene pair for each of seven possible mating types. This study shows that DNA recombination produces a large number of unusual MAT gene pairs, but through a KU-dependent process only one normal pair is retained in a mature cell; retention of unusual gene pairs leads to self-mating and suggests a system for self/non-self recognition.

## Introduction

Sexual reproduction is widespread throughout nature, providing opportunities for organisms to increase genetic variety and purge deleterious mutations. It can occur by outcrossing (combining gametes from 2 mating partners) or self-fertilization (combining gametes from the same individual). Though reproduction by self-fertilization may lead to inbreeding depression, it is not uncommon and is found in plants, fungi, and protozoans [1–3]. The mechanistic distinction between these processes rests largely on the compatibility between mating partners, for which a variety of mechanisms have been analyzed [1–7], though many remain unclear.

**Fig 1:**
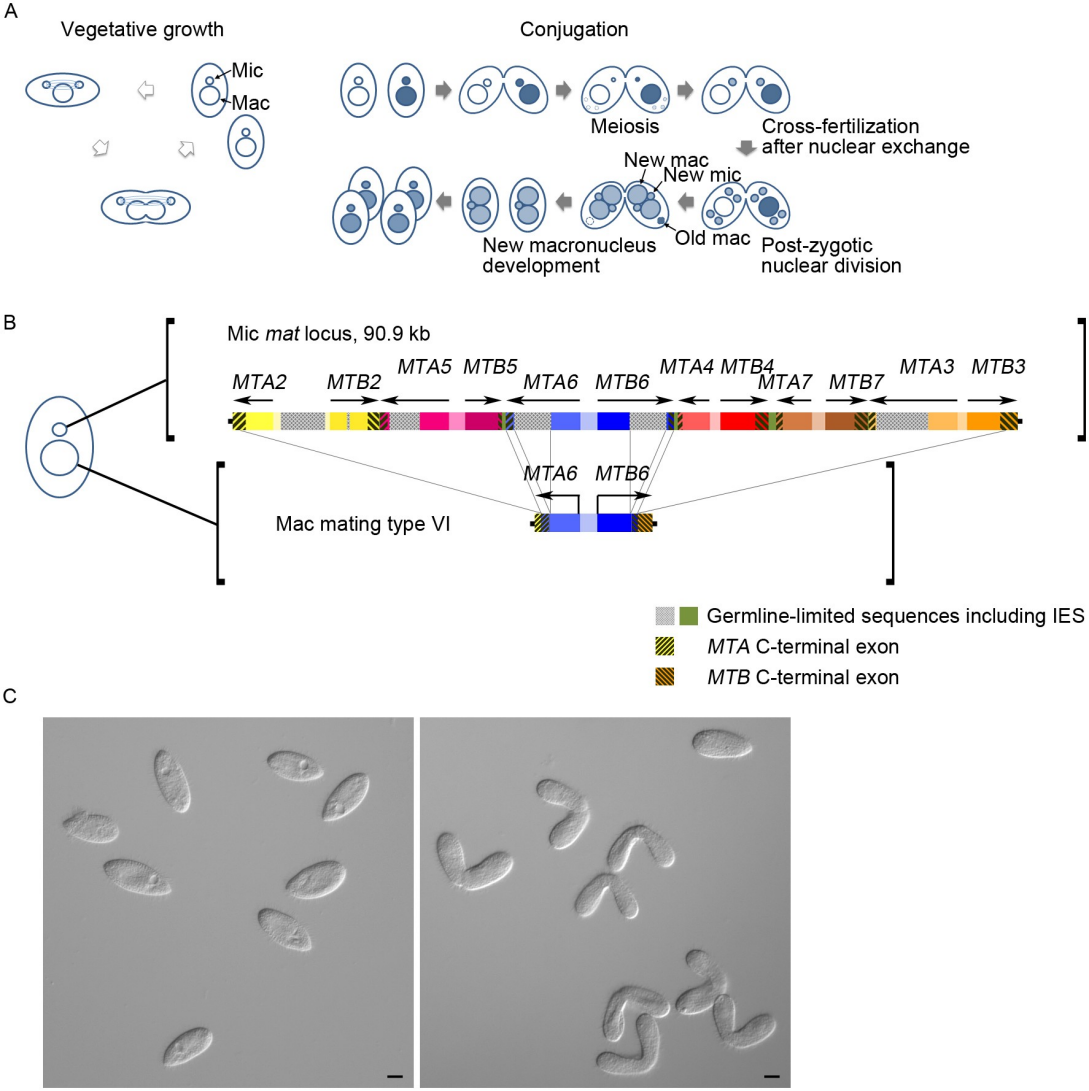
Mating type determination in *Tetrahymena*. (A) Life cycle of *Tetrahymena*. *Tetrahymena* grows asexually by binary fission (left panel). A mature cell expresses only 1 mating type during vegetative growth. The micro- and macronucleus are indicated by the small and large circle, respectively. Two sexually matured cells of different mating type can form pairs and enter the conjugation process when starved (right panel). The mating partners exchange meiotic products of the parental micronuclei and form zygotic nuclei that further divide and differentiate into the new micro- and macronuclei. Parental macronuclei degrade at the end of this process. Paired cells separate and divide to generate 4 progeny cells. (B) The *mat* locus of the micronucleus (upper panel) and the macronucleus (bottom panel) of a mating type VI cell. The connecting lines indicate DNA rearrangements including the deletion of IESs and the removal of all other mating-type gene pairs, presumably by recombination between the homologous sequences present in the *MTA/MTB* C-terminal exons [10]. (C) Selfing occurs in a Δ*TKU80* strain after starvation. Normal clonal cells do not form mating pairs (left panel), but mating pairs were observed within a clone of the Δ*TKU80* strain after starvation (right panel). Scale bar = 10 μm. IES, internal eliminated sequence; Mac, macronucleus; Mic, micronucleus.

The model ciliate *Tetrahymena thermophila*, like most protists, reproduces asexually by binary fission (Fig 1A). Growth is interrupted intermittently by the sexual process of conjugation, which occurs between cells of different mating types (Fig 1B). Interestingly, selfers—cells that form intraclonal mating pairs upon starvation—have been reported occasionally [8,9], but the mechanism behinds it remains largely unknown.

Seven mating types have been found in *T*. *thermophila*, denoted by the Roman numerals I–VII [11]. Cells of different mating types can form mating pairs after starvation and enter the sexual process (conjugation). Like other ciliates, *Tetrahymena* contains 2 kinds of nuclei with distinct functions: a silent micronucleus (germline) and an active macronucleus (soma) that determines phenotype during vegetative growth. During mating, the partners exchange the meiotic products of the parental micronuclei, which fuse to generate zygotic nuclei that further divide and differentiate into new micro- and macronuclei. The parental macronucleus is degraded and is replaced by the new macronucleus. After the mating pair separates, each cell divides once and generates 2 progeny cells (named caryonides) at the end of conjugation (Fig 1A). *Tetrahymena* mating type is thought to be determined during the differentiation of the new macronucleus [12]. Although the macronuclei of the 4 caryonides are all derived from the 2 genetically identical zygotic nuclei, they have developed independently and can determine different mating types. The young caryonides are sexually immature and require about 50 divisions to reach sexual maturity.

Mating type in *T*. *thermophila* is determined by the *mat* locus, which contains the genes for all potential mating types. Cells homozygous for the *mat-1* allele can express any of the 7 mating types except IV and VII; those homozygous for *mat-2* allele can express any but type I; and heterozygous *mat-1/mat-2* cells can express any of the 7 [11,13]. The *mat* locus has previously been mapped to an approximately 300-kb genomic region [14–16]. Subsequent genome and transcriptome sequencing analysis identified 2 candidate genes, *MTA* and *MTB*, that were arranged head-to-head at the *mat* locus [10]. Each mating type is determined by a specific *MTA* and *MTB* gene pair in the macronucleus. However, multiple incomplete *MTA* and *MTB* gene pairs are arranged in a tandem array between the complete genes (*MTA2* and *MTB3*) in the micronuclear *mat* locus (Fig 1B). In the micronucleus of *mat-2* cells, the 6 *MTA* and *MTB* gene pairs are organized as head-to-head dimers in the order [*MTA2*–*MTB2*ΔCtx]-[ΔCtx*MTA5*–*MTB5*ΔCtx]-[ΔCtx*MTA6*–*MTB6*ΔCtx]-[ΔCtx*MTA4*–*MTB4*ΔCtx]-[ΔCtx*MTA7*–*MTB7*ΔCtx]-[ΔCtx*MTA3*–*MTB3*]. Only the genes (*MTA2* and *MTB3*) at either end of the cluster are complete; the rest are missing a part of C-terminal sequences [10]. Through specific yet uncharacterized recombination, complete gene pairs are produced, and 1 gene pair is kept in the macronucleus. A model has been presented for this process [10]. Presumably, recombination between these homologous regions completes the C-terminal exon and joins the mating-type-specific N-terminal region, results in a complete, mating-type-specific gene pair in the macronucleus [10]. During conjugation, DNA endoduplication occurs in the developing macronucleus, and the macronuclear genome is amplified to about 67 copies [17]. It has been proposed that an “intranuclear coordination” process reduces the multiple types of mating-type genes to few genes, which are further reduced by random segregation (phenotypic assortment) during amitotic macronuclear division in vegetative growth [18]. In this way, the sexually matured cells usually express only 1 mating type. Once determined, the mating type is extremely stable during growth [12,19]. Cells of fixed mating types do not form intraclonal mating pairs. Clones of some young caryonides have been found to form intraclonal pairs after reaching sexual maturity [9,20]. However, when subcloned at a later stage of growth, these selfers become stabilized strains of fixed mating types. This is thought to result from the assortment of different rearranged forms of the *mat* locus during amitotic division of the macronucleus, similar to the assortment of alleles known to occur for heterozygous genes during growth [21]. Selfers have not been found in any known *Tetrahymena* mutants or in established lab strains so far.

In addition to the *mat* genes rearrangements, several types of programmed DNA rearrangements occur in the developing macronucleus during conjugation, including the deletion of thousands of internal eliminated sequences (IESs) that contain approximately 34% of the micronuclear genome, DNA fragmentation at specific chromosome breakage sequences, and endoduplication that generates approximately 67 copies of most genomic sequences and approximately 13,000 copies of the ribosomal RNA gene (rDNA) in each macronuclear genome [17]. Some of the generated minichromosomes are also eliminated between 6 and 20 fissions after conjugation [22].

Previously, we have found that *TKU80*, a conserved component of the nonhomologous end joining (NHEJ) process for repairing double-strand DNA breaks, is essential for programmed DNA rearrangements in *Tetrahymena* [23]. In the NHEJ process, Ku binds to DNA breakage sites and serves as a recruitment platform for other repair factors such as polymerases and DNA ligases IV. Ku consists of Ku70 and Ku80 subunits. Three *Ku* orthologues exist in the *Tetrahymena* genome: 2 *Ku70* (TTHERM_00684440 and TTHERM_00561799, named *TKU70-1* and *TKU70-2*) and 1 *Ku80* (TTHERM_00492460, named *TKU80*). In addition, a conserved DNA ligase IV (TTHERM_00387050) in NHEJ is also found in *Tetrahymena* genome. Although the up-regulated expression of all 3 *Ku* suggests important functions during conjugation, analysis of the 2 *Ku70* were complicated by difficulties in generating germline gene deletion mutants. In the analysis of the only *Ku80* in *Tetrahymena*, we have found that Ku80 may serve a DNA end-protective function, and a Ku-dependent DNA-repair pathway may be involved in the deletion of most IESs during conjugation. In this study, we report the surprising observation that reduced expression of *TKU80* and *TKU70-2* during specific developmental stages led to the production of stable selfers. DNA analysis showed that IES deletion at the *mat* locus occurred normally, but the overall organization of the mating-type genes was affected. Interestingly, multiple mating-type gene pairs and/or unusual mis-paired genes that resembled unfinished intermediates previously proposed [10] were detected in the macronucleus of the selfer, suggesting abnormalities in mating-type gene rearrangements and the sorting of mating-type genes in early vegetative growth. These results suggest a possible new role for Ku proteins and provide insights into mating type determination in *Tetrahymena*.

## Results

### *TKU80* mutation produced selfers

Previously we have found that Ku80 is required for DNA rearrangements during new macronuclear development [23]. However, The *TKU80* germline knockout strains (lacking *TKU80* in the micronucleus but normal in the macronucleus) were able to grow and mate normally to produce viable progeny, which now lacked *TKU80* in both nuclei (Δ*TKU80* strains). To determine their mating potentials, progeny cells were subcloned after several divisions and tested for their mating capabilities after further growth to reach sexual maturity. Unexpectedly, an intraclonal mating phenotype (selfing) was observed in some progeny—cells within the subclone population were able to form mating pairs upon starvation (Fig 1C). Although selfing can be observed in control strains, it is rare (S1 Table). In Δ*TKU80* strains about 50% subclones showed the selfing phenotype, and in each selfing line, the percentage of cells that form selfing pairs varied widely, with some exceeding 90%. The selfing pairs could be observed after 2 hours in starvation media (S1 Fig). Similar to normal cells, sexual immaturity was observed in these selfers after conjugation, and starvation was also required for inducing mating. Consistent with the previous study of *TKU80* mutants [23], these selfers (Δ*TKU80* strains) could not finish conjugation and eventually died after losing their new macronuclear DNA (S2 Fig). Although selfers have been reported in normal strains occasionally, they occur only to young progeny clones (populations derived from cells subcloned at an early stage after conjugation) [20]. To examine the selfers of Δ*TKU80* strains, we subcloned them at different fissions after conjugation and analyzed the selfing phenotype after sexual maturation (Table 1). In 3 control cell matings, selfing phenotype was observed at low rates (<6%) among subclones established during early division periods and was rarely found after approximately 20 fissions of vegetative growth. In contrast, selfing was observed frequently (>50%) even after subcloning at later growth (76 fissions). These results indicate that depletion of *TKU80* from the zygotic nuclei (but retaining its function in the parental nuclei) during conjugation and the subsequent growth leads to persistent selfing in their progeny, suggesting that zygotic function of *TKU80* is important for mating type determination in *Tetrahymena*.

**Table 1:** Selfer ratio at different fission number.

	Subcloning time^a^					
Type of mating cells	4th	10th	22nd	34th	52nd	76th
CU427 × CU428	3%	0%	0%	0%	0%	0%
BII × CU427	6%	1%	0%	0%	0%	0%
BII × CU428	3%	6%	0%	2%	1%	0%
*TKU80* germline knockout 826 × 513	64%	52%	68%	74%	70%	63%

^a^Subcloning time: estimated numbers of cell fissions after conjugation in vegetative growth. Selfing phenotype was analyzed by starvation after sexual maturation (approximately 46 to 80 fissions). *n* = 100.

### Selfing was stable through hundreds of fissions

Possible reasons for the selfing included the loss of self/nonself-recognition, frequent mating-type switching, possession of multiple mating type potentials, and others. Normally, *Tetrahymena* mating type is stably transmitted during growth [19]. We thus analyzed Δ*TKU80* selfer strains after repetitive subclonings and growth and found that the selfing phenotype could be quite stable even over hundreds of fissions. For instance, 2 selfers were subcloned at 140th fissions, and 13 of 14 subclones remained selfers. Three of the 13 selfers were further subcloned at 168th fissions, and 16 of 18 subclones maintained selfing phenotype. The most fissions that have been analyzed was 251, and all 12 subclones of 2 selfers maintained selfing phenotype. Although the selfing phenotype persisted in most progeny cells, some did generate subclones of nonselfers with fixed mating types (Table 2 and S2 Table). This change was irreversible; neither selfing phenotype nor a change in mating type was observed in these stabilized subclones. Usually 1 to 3 different mating types were derived from 1 Δ*TKU80* selfer. This phenomenon is similar to that observed in selfers collected from the wild [20]. Our results suggest that Δ*TKU80* selfers possess potentialities for multiple mating types and may express 2 or more mating-type genes after starvation.

**Table 2:** Selfers can produce nonselfers.

Selfer^a^ (subcloning time)	Subclones from the selfer: selfer/total (subcloning time)	Stabilized MT
#9 (13th)	80/87 (41st)	6 MT IV, 1 MT VI
#16 (13th)	58/71 (41st)	9 MT IV, 1 MT V, 3 MT VI
b2 (1st)	82/87 (151st)	2 MT IV, 3MT V^b^
c1 (1st)	3/88 (151st)	16 MT IV, 16 MT VI^b^
c2 (1st)	27/83 (151st)	32 MT IV^b^
e (1st)	10/88 (151st)	31 MT IV^b^
f1 (1st)	8/88 (151st)	32 MT IV^b^
f2 (1st)	10/87 (151st)	32 MT IV^b^
g4 (1st)	55/88 (151st)	7 MT IV, 25 MT V^b^
j (1st)	9/56 (151st)	30 MT II^b^
g3 (1st)	53/87 (151st)	15 MT IV, 9 MT V^b^
h3 (1st)	45/86 (151st)	21 MT II, 11 MT IV^b^

^a^Selfers (Δ*TKU80*) were the progeny of *TKU80* germline KO strains.

^b^Only part of stabilized cells were analyzed.

KO, knockout; MT, mating type.

### Selfers contained multiple mating-type genes in the macronucleus

**Fig 2:**
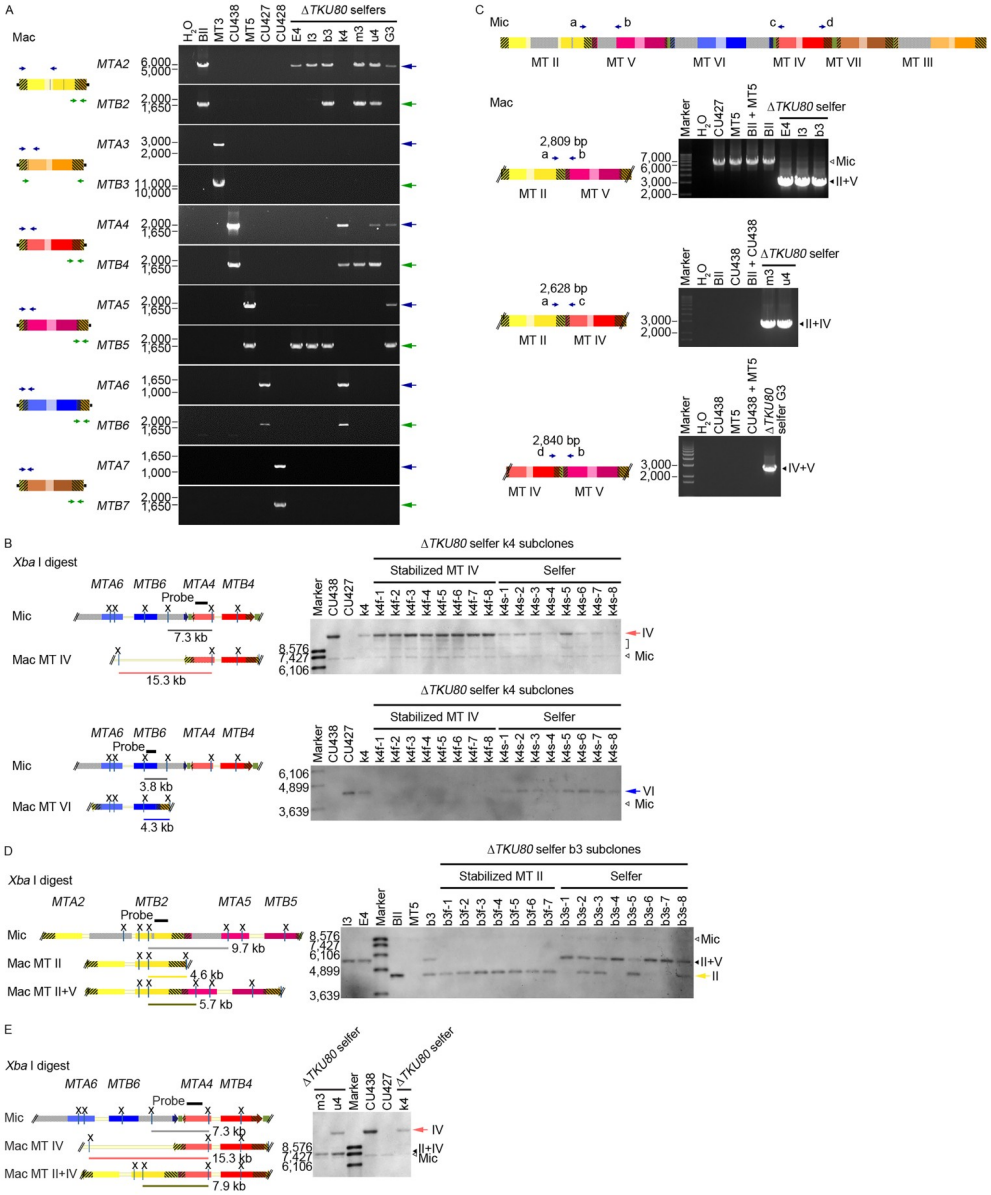
Selfers contain multiple mating-type genes in the macronucleus. (A) Analysis of the macronuclear *MTA* and *MTB* complete C-terminal exons. Left panels illustrate the macronuclear *mat* locus of 6 mating types and the primers used for detecting the *MTA* complete C-terminal exons (blue arrows) and the *MTB* complete C-terminal exons (green arrows). Black vertical lines within the gene indicate the IESs deletion junctions. DNA of 6 normal strains (strain BII, MT3, CU438, MT5, CU427, and CU428; they are of mating type II, III, IV, V, VI, and VII, respectively) and 7 Δ*TKU80* selfers were examined by PCR. The normal strains contain 1 properly rearranged pair of the *MTA* and *MTB* complete C-terminal exons. However, multiple *MTA* and *MTB* complete C-terminal exons were detected in the Δ*TKU80* selfers. Notably, the detected exon pairs were not of the same mating type in some selfers. (B) Examination of *mat* locus of MT IV and VI cells by Southern hybridization. Left panels illustrate the *mat* locus of MT IV and VI cells. IESs are indicated by gray rectangles. Probes used for detecting MT IV and VI are indicated by black bars. Xs indicate the *Xba* I sites. Cellular DNA from the Δ*TKU80* selfer k4, 8 stabilized subclones, 8 selfer subclones, and control cells was analyzed. DNA fragments corresponding to both MT IV (pink arrow) and VI (blue arrow) were detected in selfer k4 and the 8 k4 selfer subclones, whereas only the fragment corresponding to MT IV was detected in the 8 stabilized MT IV subclones. The bracket indicates nonspecific bands, and open arrowheads indicate the expected micronuclear form DNA. (C) Unusual mating-type genes are detected in Δ*TKU80* selfers. DNA of Δ*TKU80* selfers and control cells was analyzed by PCR using 3 primer pairs (blue arrows) that cannot detect the properly rearranged macronuclear forms. One pair (a+b) can detect the micronuclear form (containing IESs indicated by gray rectangles, upper panel). The unusual products presumably derived from the mixed-type gene pairs of II+V (amplified by primer a and b), II+IV (amplified by primer a and c), and IV+V (amplified by primer d and b) were detected in the selfers but not in the control cells or the mixture of 2 mating types. The expected micronuclear form was detected when using primer pair a and b in normal cells (and was likely outcompeted by the much more abundant unusual product in selfers). These unusual products are likely generated from the macronuclear forms shown in the lower left panels and are verified by DNA sequencing. (D) Examination of the *mat* locus in MT II and II+V cells by Southern hybridization. Left panels illustrate the *mat* locus of the micronucleus and the normal (MT II type) and the unusual (II+V mixed-type) *mat* genes in the macronucleus. IESs in micronucleus are indicated by gray rectangles. The probe used is indicated by the black bar. Xs indicate the *Xba* I sites. Cellular DNA from the Δ*TKU80* selfer E4, l3, b3, 7 stabilized MT II subclones of b3, 8 selfer subclones of b3, and control cells was analyzed. Unusual product corresponding to the mixed-type genes of II+V (black arrowhead) was detected in selfer E4, l3, and b3. The selfer b3 contained both fragments corresponding to MT II (yellow arrow) and II+V, which appeared to segregate in its subclones. All 8 b3 selfer subclones maintained the II+V type DNA, whereas the 7 stabilized MT II subclones contained only MT II type DNA and had lost the unusual product. (E) Examination of the *mat* locus in MT IV cells and related selfers by Southern hybridization. Left panels illustrate the *mat* locus in the micronucleus and the macronucleus in MT IV cells and selfers with the unusual II+IV type. IESs in micronucleus are indicated by gray rectangles. The probe is indicated by the black bar. Xs indicate *Xba* I sites. Cellular DNA from the Δ*TKU80* selfer m3, u4, k4, and control cells was analyzed. The unusual product corresponding to mixed-type II+IV (black arrowhead) was detected in selfer m3 and u4. Selfer u4 contains both DNA fragments corresponding to MT IV (pink arrow) and II+IV. Raw images associated with this figure can be found in S1 Raw Images. IES, internal eliminated sequence; Mac, macronucleus; Mic, micronucleus; MT, mating type.

The *Tetrahymena* micronuclear *mat-2* allele contains a cluster of 6 partial mating-type-specific gene pairs [10]. They are rearranged to produce only 1 complete gene pair in the macronucleus (Fig 1B). The macronucleus is polyploid at the time of DNA rearrangements and potentially can generate multiple mating-type gene pairs within a single nucleus if rearrangement occurs independently in each chromatid. The proportions of these gene pairs in a nucleus could be skewed if the process was not entirely random, as suggested before, possibly through a hypothetical “intranuclear coordination” [18,24,25]. When growth resumed after conjugation, these copies presumably can assort to different sister nuclei due partly to the lack of centromeric function of the macronuclear chromosomes, eventually producing cells with only 1 form of mating-type gene pair in the macronucleus. To investigate whether multiple mating-type genes existed in the macronucleus of selfers, we examined the *mat* locus of 7 persistent Δ*TKU80* selfers (subcloned 8 times over 200 fissions) that produced high rates of selfers upon subcloning. The recombined *MTA* and *MTB* C-terminal exons and the junctions from IES deletions were analyzed by PCR (Fig 2A). By using the primers locating at each *MTA* and *MTB* specific region and the primers locating at the distal *MTA2* and *MTB3* C-terminal exons, recombined complete C-terminal fragments can be amplified [10]. In the control cell, as expected, only the specific complete C-terminal exons belonging to the corresponding mating-type genes were detected. In the Δ*TKU80* selfers, however, we found multiple *MTA* and *MTB* complete C-terminal exons in each clone. In the selfer strain k4, the complete C-terminal exons of *MTA4*, *MTB4*, *MTA6*, and *MTB6* were observed, suggesting that both correctly rearranged mating-type gene pairs could be present in this clone. Indeed, upon further growth, we found subclones displaying stabilized mating type IV or mating type VI (S3 Table). Further analysis by Southern hybridization confirmed that these stabilized mating type IV subclones contained only mating type IV and not the mating type VI gene in the macronucleus, whereas the selfer k4 and its subclones with selfing phenotype contained both mating type IV and VI genes (Fig 2B). In addition, the amount of mating type IV DNA of the selfer k4 and selfer subclones appeared to be lower than in the mating type IV control CU438 and in the stabilized mating type IV subclones. It suggests that both of the IV and VI mating-type genes may constitute the macronuclear copies of *mat* locus in these selfers. Interestingly, in the remaining selfer strains, we detected unusual gene structures. In the selfer strains E4 and l3, *MTA2* and *MTB5* complete C-terminal exons were detected, whereas their corresponding partners *MTB2* and *MTA5* complete C-terminal exons were absent (Fig 2A). It suggested that the macronucleus contained unusual products with mis-paired *MTA* and *MTB* genes. In the other 4 selfer strains, the presence of both mixed types and normal types were suggested (Fig 2A). In strain b3, *MTA2*, *MTB2*, and *MTB5* complete C-terminal exons were found but not the *MTA5* complete C-terminal exon. Similarly, selfer strain m3 contained *MTA2*, *MTB2*, and *MTB4* complete C-terminal exons; selfer strain u4 contained *MTA2*, *MTB2*, *MTA4*, and *MTB4* complete C-terminal exons; selfer strain G3 contained *MTA2*, *MTA4*, *MTA5*, and *MTB5* complete C-terminal exons. Interestingly, these later strains generated subclones with stabilized mating types corresponding to the paired *MTA* and *MTB* complete C-terminal exons that they have (S3 Table). These results suggest that the deficiency of *TKU80* led to the persistence during prolonged vegetative growth of, in a single cell, multiple mating-type gene pairs as well as unusual mis-paired mating-type genes and the selfing phenotype. The deletion of IESs in *mat* locus, however, remained normal.

### Unusual rearrangements of mating-type genes in selfers

The aforementioned analysis described suggested unusual rearrangements in selfer strains. Selfer E4 and l3 contained the complete C-terminal exons of *MTA2* and *MTB5* and incomplete C-terminal exons of *MTB2* and *MTA5* in the macronucleus, suggesting the absence of further recombination that retained the middle part of the mating type II and V region including the incomplete *MTB2* and *MTA5* (*MTB2*ΔCtx and ΔCtx*MTA5*) (Fig 2C). The middle region was thus examined by PCR and sequencing, which confirmed this prediction. We found the same unusual product [*MTB2*ΔCtx]-[ΔCtx*MTA5*] sequence in the selfer strains E4, l3, and b3. This DNA segment was very similar to that in the micronucleus, containing the left end of the locus (*MTA2*) continuing through the partial *MTB2* and *MTA5* genes and ends with a properly rearranged *MTB5* gene at the right end [*MTA2*-*MTB2*ΔCtx]-[ΔCtx*MTA5*-*MTB5*] and with the IESs deleted normally. Further examination by Southern hybridization confirmed the presence of this structure in all 3 strains (Fig 2D). The selfer E4 and l3 contained only this II+V band, whereas selfer b3 also contained a band corresponding to the normal mating type II [*MTA2*–*MTB2*] gene pair. Interestingly, all subclones of E4 and l3 were selfers (S3 Table), and no independent assortment was found by repeated subcloning. The genetic linkage is consistent with the proposed structure [*MTA2*-*MTB2*ΔCtx]-[ΔCtx*MTA5*-*MTB5*]. On the other hand, some subclones of b3 became normal mating type II. Southern hybridization results of the b3 subclones revealed the assortment of these 2 DNA forms into different subclones, which corresponded to their mating behaviors (mating-type II or selfer). This result further supports the conclusion that the unusual DNA structure confers the selfing phenotype. The situation in the selfer strains m3 and u4 appeared rather similar (S3 Table). In the selfer u4, *MTB2* could be detected by PCR but not by Southern hybridization, suggesting a low abundance of the normal mating-type II genes. Both m3 and u4 strains contained 2 forms of mating-type genes in the macronucleus: an unusual II+IV form (Fig 2C and 2E) and a normal mating type II (in m3) or mating type IV (in u4). However, this II+IV form was quite different from that existed in the micronucleus. Through PCR and DNA sequencing, we found that the sequence in this form was arranged in the order of [*MTB2*ΔCtx]-[ΔCtx*MTA5*]/[ΔCtx*MTA4*]. Because of their sequence homology at the C-terminal exon, the exact crossover point between the *MTA5* and *MTA4* sequences could not be identified. The other selfer strain G3 contained yet another unusual form IV+V (Fig 2C). DNA sequencing analysis revealed the rearranged sequence order as [*MTB4*ΔCtx]/[*MTB2*ΔCtx]-[ΔCtx*MTA5*]. Taking together, these results indicated that some Δ*TKU80* selfers contain complete *MTA*/*MTB* genes that are of different mating type specificity (mis-paired genes), in addition to other incomplete mating-type genes. Presumably these mis-paired genes can confer mating reaction but with abnormal mating-type identities. Some strains also contained additional copies of properly rearranged mating-type gene pairs, which can be assorted and became pure in some subclones that also mated according to this type. If the selfer possessed only the unusual product of DNA, the selfing phenotype would persist without generating nonselfing subclones during growth.

### Formation and propagation of unusual mating-type DNA

**Fig 3:**
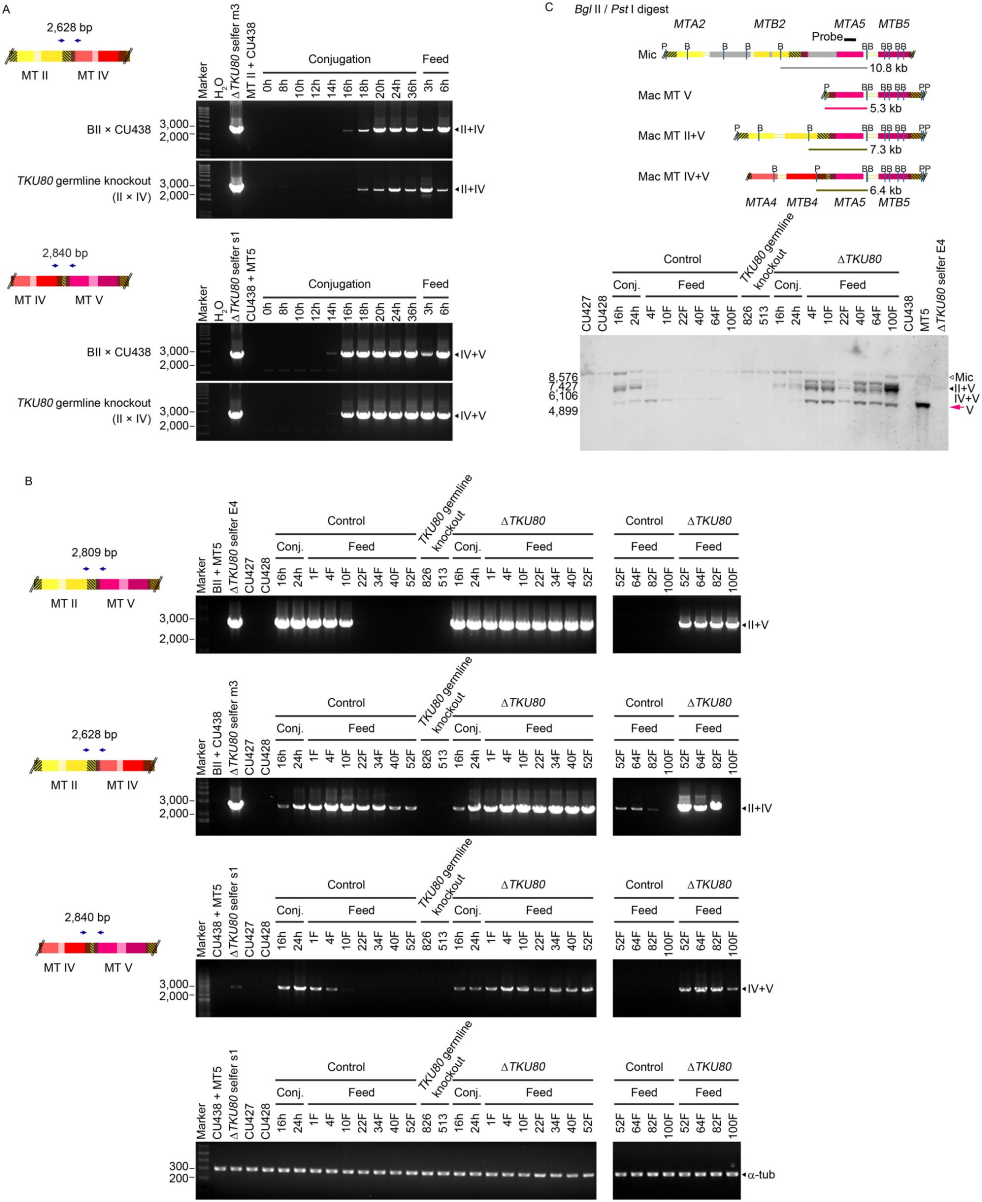
Unusual *mat* gene structures are formed during conjugation. (A) Detection of the unusual II+IV and IV+V mixed-type genes in the macronucleus during conjugation. Left panels illustrate the presumed unusual *mat* gene structures and the PCR primer locations. Cellular DNA of Δ*TKU80* selfers, normal cells, conjugating Δ*TKU80* normal cells at different time points (0, 8, 10, 12, 14, 16, 18, 20, 24, and 36 hours post-mixing) and after conjugation and feeding (3 and 6 hours) was examined by PCR. These unusual products were detected at late conjugation in both normal and mutant cells, suggesting that they could be unfinished *mat* rearrangement intermediates [10]. (B) Unusual *mat* gene products persist long after conjugation in mutants but not normal cells. Left panels illustrate the unusual *mat* gene structures and the locations of the PCR primers. DNA of Δ*TKU80*, normal cells, conjugating Δ*TKU80* and normal cells at different hours during conjugation (16 and 24 hours post-mixing) and at different numbers of fission after conjugation (1F, 4F, 10F, 22F, 34F, 40F, 52F, 64F, 82F, and 100F) was examined by PCR. α-tub gene served as the template control. These likely unfinished intermediates were detected at late conjugation and were lost later during vegetative growth in normal cells. In Δ*TKU80* cells, these unusual structures were maintained in vegetative growth. (C) Examination of unusual *mat* genes during and after conjugation by Southern hybridization. Left panels illustrate the *mat* locus in the micronucleus and the normal and unusual structures in the macronucleus. IESs in the micronucleus are indicated by gray rectangles and the probe by the black bar. Bs and Ps indicate the *Bgl* II and *Pst* I sites, respectively. Cellular DNA from normal cells, *TKU80* germline KO strains, Δ*TKU80* selfer E4, and normal and *TKU80* mutant cells during and after conjugation was analyzed. In normal cells, these unusual structures were observed in late conjugation and disappeared at approximately 10 fissions after conjugation and feeding. However, they were abundantly present and did not disappear in progeny of Δ*TKU80* cells. Raw images associated with this figure can be found in S1 Raw Images. Conj, conjugation; F, fissions; IES, internal eliminated sequence; Mac, macronucleus; Mic, micronucleus; MT, mating type.

To investigate the occurrence of unusual rearrangements in the *mat* locus, we examined *TKU80* mutant cells at different time points during and after conjugation. The unusual products were detected by PCR in both normal and *TKU80* mutant cells at and after the 14–16 hours during conjugation (Fig 3A). They decreased and disappeared soon after conjugation in normal cells but were maintained for over 100 fissions during vegetative growth in Δ*TKU80* cells (Fig 3B and 3C). Southern hybridization analysis showed that these unusual forms were abundantly present in those cells (Fig 3C).

These results suggest that the unusual forms are generated during the normal processes of rearrangements in late conjugation either as intermediates or by-products but are removed within several fissions after conjugation. In the absence of *TKU80*, however, these forms were somehow maintained in the macronucleus.

### Silencing *TKU70-2/80* after conjugation produced selfers

**Fig 4:**
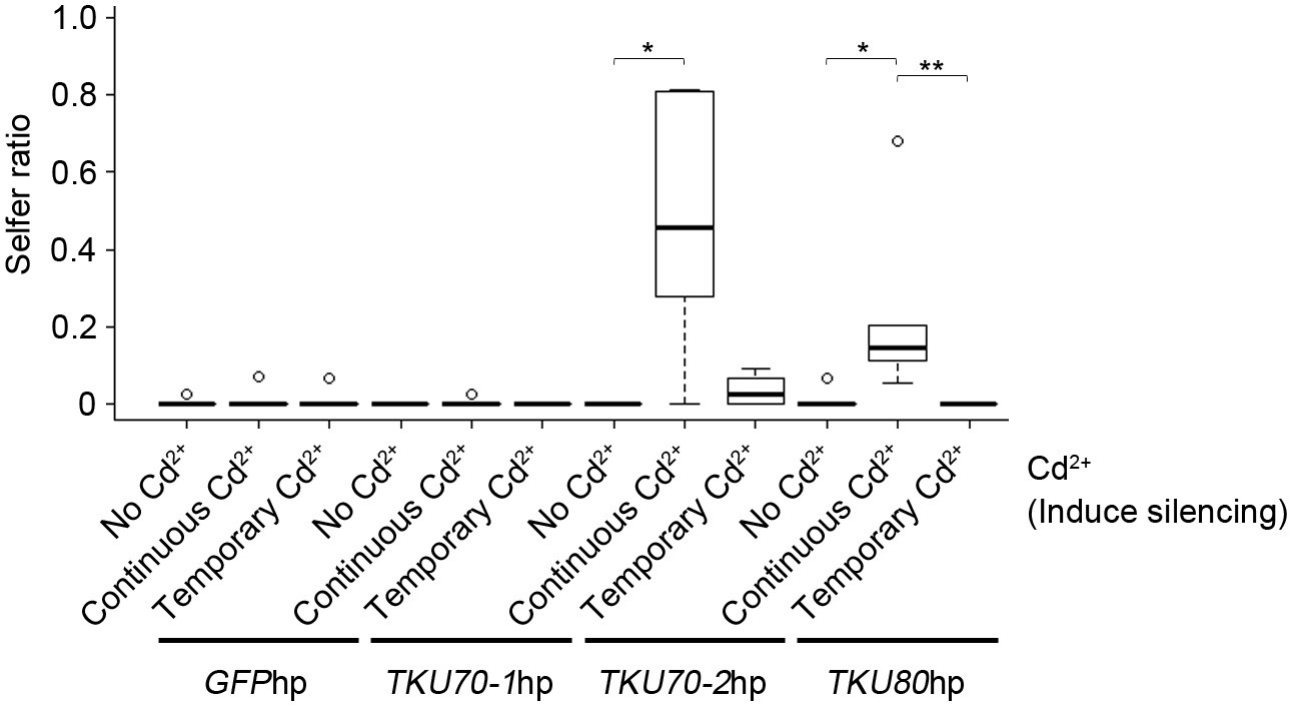
Selfers are generated by silencing *TKU70-2 or TKU80* after conjugation. Hairpin RNA to induce gene silencing was induced at approximately 1 fission time and terminated at approximately 22 fissions or continuously during vegetative growth. The ratios of selfing cells without silencing, temporary silencing (1–22 fissions), and continuous silencing are measured and shown in the boxplot. Selfing was examined after 69 vegetative fissions. Selfers increased significantly after silencing *TKU70-2* or *TKU80* (*n* = 5; *p*-values: *p < 0.05; **p < 0.01, Wilcoxon rank-sum test) but not *TKU70-2* or the GFP control. The data underlying this figure can be found in S1 Data. Thick horizontal lines represent medians of each distribution, the open box shows the middle 2 quartiles, and the circles show outliers. GFP, green fluorescent protein.

Earlier studies indicated that Ku80 is required for programmed DNA rearrangements during conjugation. However, its role in mating type determination might be different and could potentially occur in late conjugation and/or during growth. To address this question, hairpin RNA-induced gene silencing (RNAi) was adopted. We introduced an inducible hairpin RNA expression vector into conjugating cells, induced the silencing effect (S3 Fig) at various points during or after conjugation and tested these cells for selfing after they had reached sexual maturity (Fig 4 and S4 Table). Remarkably, we found that selfers could be generated by inducing *TKU80* silencing even after conjugation (Fig 4). About 24% of progeny cells exhibited selfing phenotype when *TKU80* silencing was induced after conjugation and cells began to grow (approximately 1 fission). Selfers could still be observed when *TKU80* silencing was induced after 20 fissions. Furthermore, no selfer was observed if *TKU80* silencing was induced at 1 fission but the silencing removed at approximately 22 fissions. Only approximately 5% progeny remained when the silencing was removed at approximately 29 fissions. These results indicated that *TKU80* expression was important for mating type determination even after the time of DNA rearrangements. This effect allowed us to examine the role of the other Ku protein genes, *TKU70-1* and *TKU70-2*, for which knockout mutations could not be generated after repeated tries. Silencing *TKU70-2* after conjugation also generated selfers. About 47% of progeny cells were selfers if *TKU70-2* silencing was induced at approximately 1 fission time. Removing the silencing at approximately 29 fissions also greatly reduced selfing progeny (approximately 2%). On the other hand, silencing of *TKU70-1* had no effects on the production of selfing progeny, agreeing with our expectation because *TKU70-1* is expressed only during conjugation. These results suggest that Tku70-2p and Tku80p are involved in mating type determination after conjugation.

### Phenotypic assortment was not significantly changed in the absence of *TKU80*

**Fig 5:**
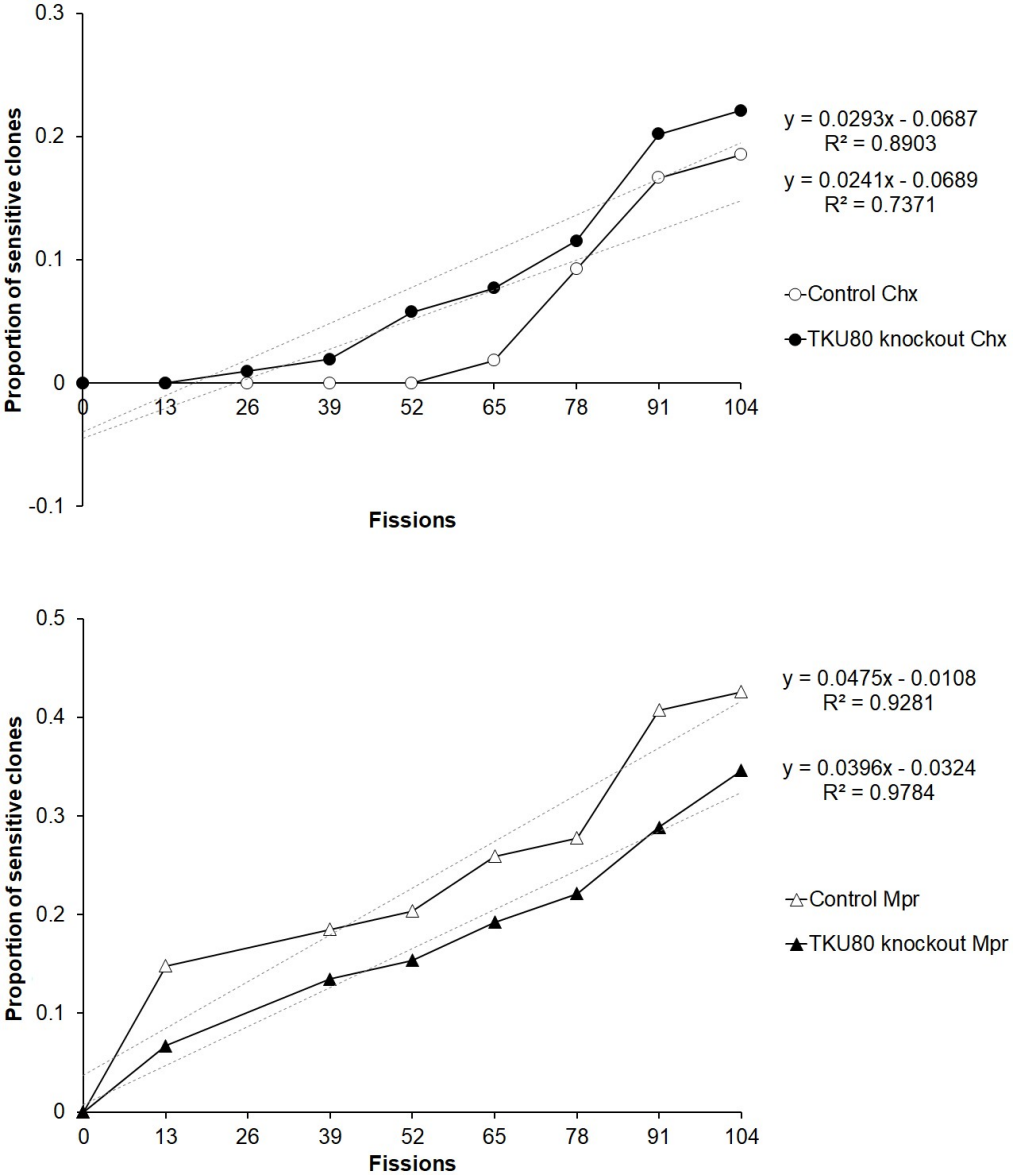
Phenotypic assortment was not significantly affected in other loci in the absence of *TKU80*. Caryonides containing heterozygous *Chx* and *Mpr* were examined in serial vegetative fissions. The cumulative proportions of drug-sensitive clones in different fissions are illustrated in up (cycloheximide) and down (6-methylpurine) panels with the regression lines (gray dash) and regression equations. The difference of the slops was not significant between control and Δ*TKU80* cells in *Chx* (*p* = 0.4797, *t* test) and *Mpr* (*p* = 0.2229, *t* test). *n* = 54 (control cells); *n* = 104 (Δ*TKU80* cells). The data underlying this figure can be found in S1 Data. Chx, cycloheximide resistance; Mpr, 6-methylpurine resistance.

During vegetative growth, the macronucleus divides amitotically; thus, a heterozygous locus can become homozygous eventually through assortment even in the absence of selection. This assortment could explain why some selfers were able to produce stabilized clones of fixed mating types in earlier studies [13]. Because multiple mating-type genes existed stably in some *TKU80* mutant selfers, it could be due to some abnormalities or delay in the sorting of these genes. To investigate whether phenotypic assortment in general was affected in the Δ*TKU80* strains, we examined the assortments of 2 established antibiotic-resistance makers *Chx* and *Mpr* (Fig 5). Compared with the control cells, the assortment behavior of the 2 genes in Δ*TKU80* strains did not significantly change. The result suggests that phenotypic assortment in general is not affected at the absence of *TKU80*.

### Mating-type recognition (self/nonself–recognition) in *T*. *thermophila*

**Fig 6:**
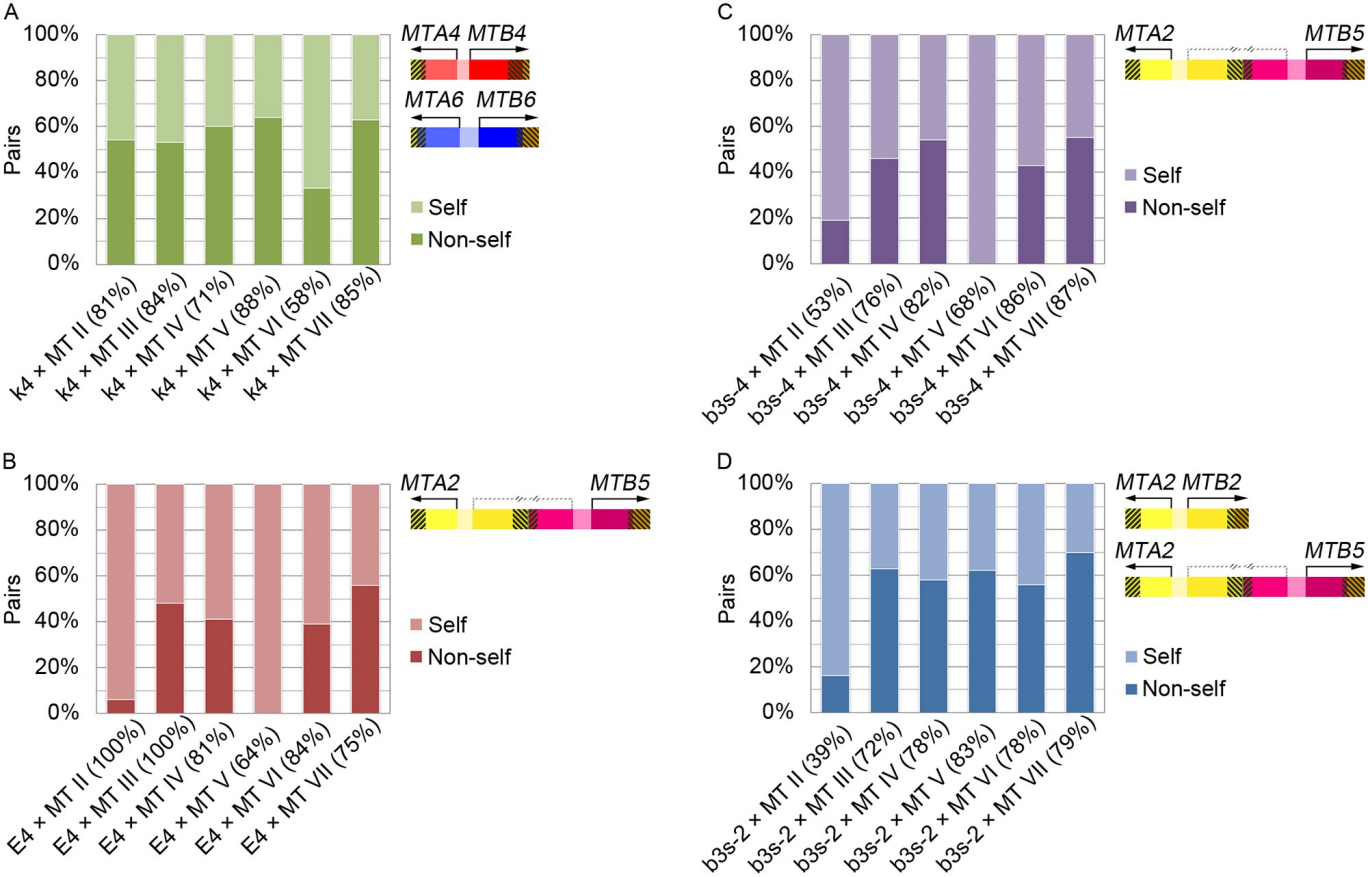
Mating compatibility of the selfers. Tester cells of 6 different MTs were labeled with NHS-Rhodamine. After starvation, selfers were mixed with tester cells at the ratio of 2:1. Selfing and/or nonselfing (pairing with tester) pairs were fixed and analyzed under a microscope at 4 hours post-mixing. (A) Selfer k4 (containing both MT IV and MT VI genes) formed mating pairs with all 6 tester cells. (B) Selfer E4 (containing mixed-type II+V genes) formed mating pairs with 5 tester cells but not with MT V cells. (C and D) Subclones of selfer b3 that containing only the mixed-type II+V genes showed the same mating compatibility as selfer E4. (D) Subclones of selfer b3 that contained both MT II and the mixed-type II+V genes could form mating pairs with all strains including MT V cells. Pair ratio is indicated in the parentheses. *n* = 100 pairs. The data underlying this figure can be found in S1 Data. MT, mating type.

The selfing phenotype raises the question of whether the selfers are able to mate with cells of any mating type. To investigate their mating compatibilities, we mated the selfers with tester strains labeled with a fluorescent dye, so that selfing and nonselfing pairs could be visually distinguished (S4 Fig). After starvation, a tester strain of a specific mating-type was mixed with the selfer, and the formation of mating pairs were analyzed (Fig 6 and S5 Fig). We found that the selfer strain k4, which contained mating type IV and mating type VI genes, could form mating pairs with all 6 different mating-type cells at different mixing ratios (Fig 6A and S5 Fig). Selfer strain E4, which contained only the mis-paired II+V (*MTA2*/*MTB5*) genes, formed mating pairs with mating type III, IV, VI, and VII cells, but poorly with mating type II (*MTA2*/*MTB2*) cells and failed totally with mating type V (*MTA5*/*MTB5*) cells (Fig 6B). This phenomenon was not changed when mixing with different ratios of tester strains (S5 Fig). In the mating with mating type II cells, some single cells with meiotic micronuclei or crescent were found at the early stage, suggesting that *MTA2*/*MTB5* cells formed loose pairs with *MTA2*/*MTB2* cells to trigger meiosis before aborted pairing. Selfer strain b3s-2 and b3s-4 were both subcloned from selfer strain b3, which contained mating type II genes (*MTA2*/*MTB2*) and mis-paired *MTA2*/*MTB5*. They differ in that b3s-2 contained both mating type II and II+V, whereas the b3s-4 only contained II+V and lost mating type II (Fig 2D). Similar to strain E4, low mating pairs were formed between selfer b3s-4 (*MTA2*/*MTB5*) and mating type II (*MTA2*/*MTB2*) cells but not at all between selfer b3s-4 (*MTA2*/*MTB5*) and mating type V (*MTA5*/*MTB5*) cells (Fig 6C). On the other hand, selfer b3s-2 (*MTA2*/*MTB2* and *MTA2*/*MTB5*) cells were able to form mating pairs with mating type V (*MTA5*/*MTB5*) cells (Fig 6D). We further analyzed expression of *MTA*/*MTB* in these selfers by reverse transcription polymerase chain reaction (RT-PCR) and found that multiple mating-type genes were expressed and the transcripts found were consistent with the mating-type genes they possessed (S6 Fig). Although the expression of the C-terminal truncated genes were also observed, they are not likely to produce functional proteins for lacking the complete C-terminal exons that presumably encode transmembrane helices [10]. These results indicated that selfers possessing multiple mating-type gene pairs could mate with all mating-type cells including themselves and behaved as no mating type at all. On the other hand, selfers that contained only 1 mis-paired mating-type genes could form pairs with the other mating-type cells but not with the same *MTB* cells, and they were able to formed loose pairs with the same *MTA* cells. This difference between the effects of *MTA* and *MTB* is reminiscent of the difference in knocking out *MTA* or *MTB* genes [10]. *MTB* knockout abolished the cell’s ability to pair and the *MTA* knockout cells paired poorly when mixed with normal cells of a different mating type [10]. Our results further support the earlier notion that *MTA* and *MTB* have different functions in self/nonself-recognition.

## Discussion

The conserved Ku proteins play key roles in DNA repair in most organisms. Unexpectedly, we found that abolishing Ku protein gene function perturbs mating type determination and generates selfing clones in *Tetrahymena*. Detailed analysis uncovers unusual DNA rearrangement steps in *Tetrahymena* mating type determination, sheds light on the molecular nature of self–nonself-discrimination, and reveals an unexpected phase of genome maturation during growth of this ciliate. Ku proteins appear to have an interesting role in modulating inbreeding of an organism that possesses multiple mating types.

### Not mating-type switching but the possessing of multiple mating-type genes in selfers

Selfing was first observed in a wild collection of *Tetrahymena* in 1952, an AA strain, which, like the *TKU80* knockout selfers, failed to generate viable progeny [8,26]. The strain no longer exists, and the molecular basis of its selfing remains unknown. Previously we have found that without *TKU80*, the macronucleus is unable to complete DNA rearrangements, and the DNA is degraded during conjugation [23]. Here we found that if *TKU80* is deficient only in the zygotic nucleus but is present in the old macronucleus, global DNA deletion is normal (S7 Fig), and the progeny is viable but performs selfing. It suggested a late function for Ku80 in mating type determination. Furthermore, silencing of *TKU80* after conjugation also generated selfers that contain similar gene structures (S8 Fig). Thus, the stage of Ku action for mating type determination is distinctly different from its action on global DNA deletion.

In the budding yeast, mating-type switching occurs after mitotic division to enable intraclonal mating [6,7]. Mating-type switching has also been found in the ciliate *Blepharisma japonicum* [4]. In *Candida albicans*, presumed same-sex mating can be induced by environmental stresses [27]. In these cases, each cell expresses no more than 1 mating-type determinant. In the ciliate *Euplotes raikovi*, selfing has been observed in 1 heterozygous (*mat-1/mat2*) strain (strain 13) that encodes 2 mating pheromones [5]. In our study here, we found that a single *T*. *thermophila* cell can contain multiple mating-type genes to enable selfing. This is likely facilitated by the polyploidy nature of the macronucleus and its potential to generate multiple mating-type determining genes from a single inherited allele.

### Mating type determination in *T*. *thermophila*

In *Tetrahymena*, complex DNA rearrangements produce 1 complete mating-type genes pair in the new macronucleus to specify 1 mating type. The exact mechanism of the rearrangement is not known. Previous reports suggested an sequential intrachromosomal recombination process between either end of the conserved C-terminal exons of the *mat* locus and those incomplete *MTA*/*MTB* genes inside [10,18]. During this process, the incomplete *MTA* and *MTB* could be excised in circular forms as by-products or potential intermediates. Through several such recombination events, a single mating-type gene pair was formed. This model provided a nice explanation for the generation of only 1 complete gene pair from an array of incomplete genes. In selfing cells, we have uncovered 2 types of DNA abnormality: dual normal *MTA*/*MTB* gene pairs and/or mis-paired *MTA*/*MTB* genes. It has been shown that roughly half of the newly developed macronuclei are heterogeneous, with multiple potentials for mating types [24,25]. The heterogeneity usually consisted of 2 mating types (occasionally 3) as the newly developed macronucleus starts to divide, and the mating type combinations were not entirely random. This observation led to the suggestion that a certain “intranuclear coordination” process might have taken place to reduce mating type diversity during macronuclear development [18,24,25]. Upon growth and amitotic divisions, this heterogeneity was gradually sorted out such that the subclones were mostly of 1 pure mating type. It remains unclear whether Ku plays a role in the proposed nuclear formation process. On the other hand, the presence of 2 different normal gene pairs in a cell after prolonged growth (more than 100 fissions) suggests cosegregation, possibly through direct or indirect physical linkage of these mating-type genes during amitosis. This sorting problem appears specific to the *mat* genes because it did not occur to the other 2 loci examined. Interestingly, secondary recombination events have been detected in mating-type genes during growth and are thought to have resulted from gene conversion among multiple *mat* gene copies [10]. We did not examine these secondary recombination events in the *MTA*/*MTB* C-terminal exons of the selfers. If this recombination occurred or were elevated in the absence of Ku, it could potentially add to the complexity of mating-type genes either through linking of different *mat* genes and/or delaying their sorting during vegetative growth. This possibility is in line with our finding that selfers can be generated by *Ku* silencing after conjugation.

The other defect uncovered here is the presence of mis-paired *MTA*/*MTB*, which specifies 2 mating types in a single gene pair. We have detected mis-paired genes *MTA2*/*MTB5* and *MTA2*/*MTB4* and suspect that more probably exist. Remarkably, these unusual DNA forms are also present abundantly in normal strains during conjugation and thus, could be intermediates or by-products of the normal rearrangement process. Similar structures have been proposed as rearrangement intermediates to explain *Tetrahymena mat* gene formation [10,18]. Our observations provide direct supports for this idea. Along this line, we have also sought for possible circular forms of the mis-paired *MTA*/*MTB* by PCR using inverse primers but had failed to detect them in the selfers. However, it does not rule out the possibility that circular intermediates are produced in normal mating cells, and the particular structure [*MTB4*ΔCtx]/[*MTB2*ΔCtx]-[ΔCtx*MTA5*] could also be derived from some circular rearrangement intermediates. To further speculate along this line, the unusual form II+V could be generated by several recombination events to remove the incomplete mating-type gene pairs III, VII, IV, and VI following the previously proposed model [10,18], or from homologous recombination between *MTB5* and *MTB3* C-terminal exons and directly excised the other 4 incomplete mating-type gene pairs in 1 event (S9 Fig). The unusual form [*MTB4*ΔCtx]/[*MTB2*ΔCtx]-[ΔCtx*MTA5*] could be generated by the reinsertion of an excised circular form into the chromosome (S9 Fig) or by an interchromosomal recombination event following intrachromosomal recombinations, for instance, to switch the mating type II into IV in the unusual form II+V. These unusual intermediates imply that the recombination may not be limited to the C-terminal exons of the mating-type genes as the previous model has suggested [10,18]. These unusual forms disappeared only after about 10 fissions after conjugation in normal cells, providing opportunities for further *mat* gene rearrangements after conjugation. Remarkably, in mutants these forms persisted for more than 100 fissions. Thus, the effects of Ku proteins deficiency are not in the construction but rather, in the retention of these unusual forms. This is a surprising finding. It suggests that *mat* gene recombination is regulated not only in its construction but also in its retention, and the selfing phenotype from Ku deficiency appears to be a failure in the second step. In this regard, it is interesting to consider that both types of DNA abnormality revealed here, the retention of mis-paired genes and the retention of dual *mat* rearranged forms described previously, may be due to defects in the same or related Ku-dependent process.

The disappearances of these rearrangement intermediates/by-products in normal cells, interestingly, appear to coincide in stage with that of programmed minichromosome elimination [22]. Thus, the early growing period after conjugation (10–20 fissions) marks an important and interesting stage of macronuclear genome formation, during which products of DNA rearrangements that are no longer needed, including some minichromosomes, excised IESs [28–30], and the extra *mat* genes, are selectively removed. How they occur and whether they shared a molecular mechanism will be interesting to reveal. We have examined the elimination of 10 minichromosomes in the Δ*TKU80* cells and found it to be normal; thus, Ku is not required for all of these events (S10 Fig). How Ku enables the disposition of unwanted *mat* rearrangement products or help to assort 1 pure *mat* form is yet unclear. It is not apparent that the evolutionary conserved function of Ku in DNA repair is involved. However, because Ku80 (*TKU80*) and Ku70 (*TKU70-2*) are both required here, they may also form the typical heterodimeric protein complex with an ring-like structure with sequence-independent DNA binding ability [31]. Perhaps its function is related to the distribution of *mat* forms toward the 2 daughter cells as Ku has been shown to help localize telomeres to the nuclear periphery in yeast [32]. The molecular action of Ku in mating-type DNA disposal/distribution remains an intriguing question.

The rearrangements of *mat* genes also include the deletions of 6 IESs [10], which were thought to proceed by a different mechanism from *mat* gene recombination [10]. Indeed, we observed the appearance of *mat* gene recombination intermediates at a time after the deletion of these 6, and most other IESs in the genome, are completed (Fig 3) [28,29].

### Mating type recognition in selfers

**Fig 7:**
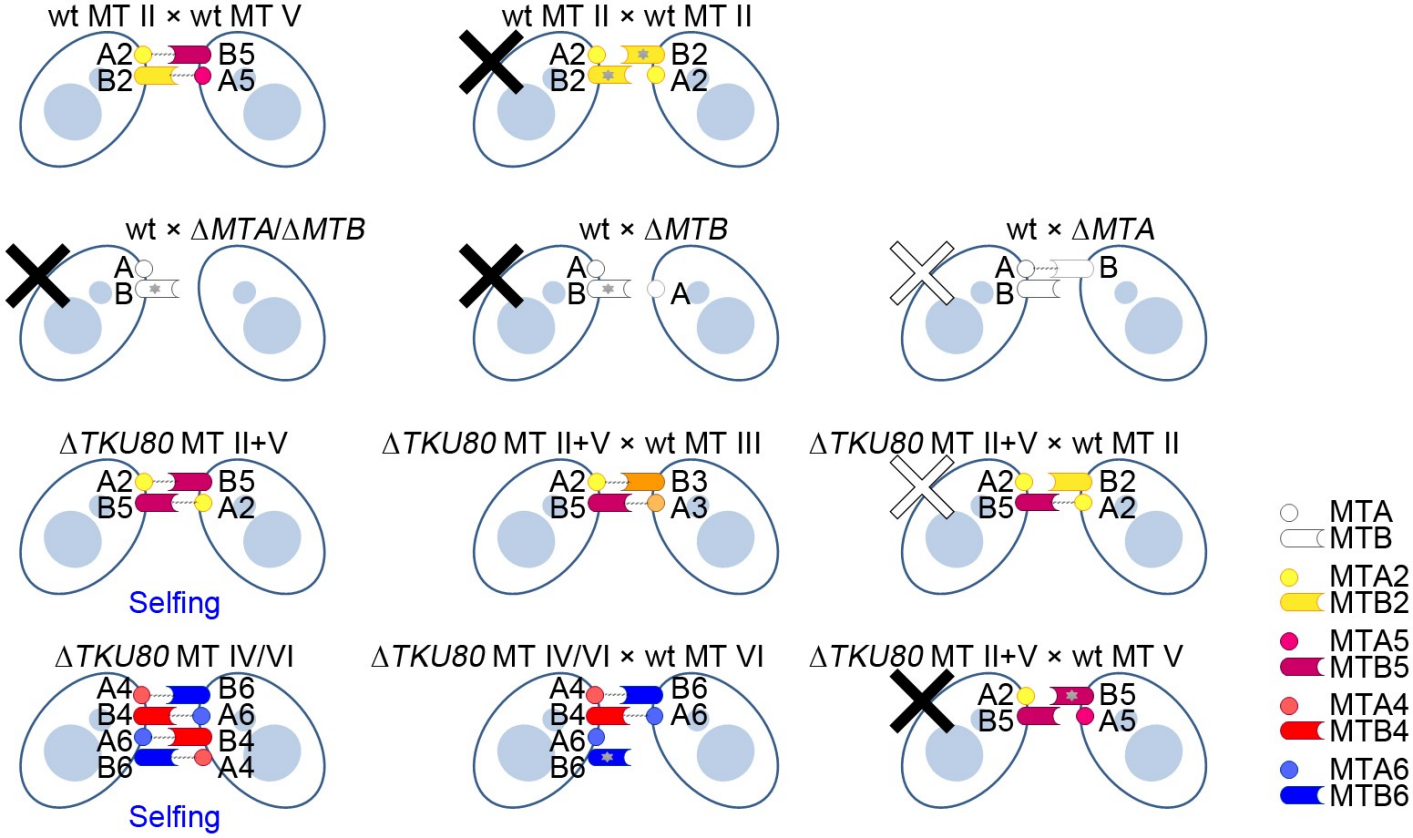
Predicted model of mating compatibility in *T*. *thermophila*. The 2-component model of self/nonself-recognition for mating is illustrated. MTA of 1 mating partner interacts with different mating-type MTB of the other partner reciprocally (dash line). Cells of the same mating type cannot form mating pairs because of the lack of either interaction (solid X mark). Although there was only 1 interaction of MTA and MTB, loose mating pairs formed if cells possessing different MTB (hollow X mark); no mating pair formed if cells possessing the same MTB (solid X mark). When the selfer II+V (MTA2/MTB5) cells encounter normal MT II (MTA2/MTB2) cells, the active form of MTB5 (without MTA5 inhibition) forms stable interaction with MTA2 of MT II, which allows loose pairs to form. When the selfer II+V (MTA2/MTB5) encountering normal MT V (MTA5/MTB5) cells, no interaction occurs between the selfer MTB5 and the MT V MTA5. Therefore, the MTB5 of MT V is inhibited by MTA5, and thus, no pairing occurs. Wt represents normal mating-type strains. MT IV/VI represents multiple *mat* alleles of both MT IV and MT VI. MTA and MTB are represented by A and B, respectively. Gray stars mark the inactive MTBs. The matings involving *MTA*/*MTB* knockout strains (second row) are taken from a previous report [10]. MT, mating type.

Three steps can be recognized in normal mating reaction in *Tetrahymena*. The first “initiation” step consists of roughly one hour of starvation at 30 °C. The second “co-stimulation” step demands cell–cell contact between different mating types for about 1 hour before the final step of mating pair formation [33,34]. A cell co-stimulated by another cell of different mating type can form a mating pair immediately with any other co-stimulated cell of a complementary mating type but not of the same mating type [35]. Thus, both co-stimulation and mating pair formation requires self/nonself-discrimination. How *MTA* and *MTB* help distinguish self from nonself is not yet clear. It has been suggested that these 2 proteins attach to the plasma membrane through their putative transmembrane domains to facilitate cell–cell contact and initiate mating reactions [18,36]. The *MTA*/*MTB* double-knockout strains cannot pair with normal cells [10]. *MTB* deletion alone abolishes paring with normal cells, whereas *MTA* deletion allows cells to form unstable mating pairs, indicating a different essential role for each gene [10]. Our analysis of the selfing strains provides new insights and suggests a 2-component scheme for self/nonself-recognition (Fig 7), in which 2 pairs of MTA and MTB interactions, each between different mating types, are required. We suggest that MTB of 1 mating partner interacts with MTA of the other partner in a reciprocal fashion, and this pair of interactions can occur only between proteins that belong to different mating types (e.g., MTB2 interacts with MTA5). These interactions activate MTB and MTA differently (further discussed next) in both cells and eventually lead to stable pairing. In this way, cells of the same mating type cannot form mating pairs because of the lack of either interaction, but selfers can form both interactions within a clone. The model further predicts that selfers with mis-paired *MTA*/*MTB* genes can form only 1 of the 2 required interactions when trying to pair with a normal strain that has either mating-type gene present in the mix pair and thus form only loose pairs or no pair at all, possibly from the failure in pair formation or co-stimulation. On the other hand, they should form stable pairs with normal cells of the other 5 mating types, and this prediction is born out in our tests. Similarly, the selfer with II+V mis-paired genes (*MTA2*/*MTB5*) can form mating pairs well with the normal type III, IV, VI, and VII cells, but form only transient pairs with type II (*MTA2*/*MTB2*) and not at all with type V (*MTA5*/*MTB5*) cell. Interestingly, selfers that contain dual mating-type gene pairs are able to form stable pairs with normal cells of any mating types. This is in good agreement with our prediction. Furthermore, it indicates that the presence of the same mating-type genes in both mating partners does not prevent pairing. Our results also suggest different roles for MTA and MTB, as were previously shown [10]. To explain this difference, we suggest that MTB is normally inhibited by MTA of the same type within a cell. Activation of MTA by interacting with a different MTB of the mating partner during co-stimulation would release this inhibition. We suggest that this inhibition on MTB needs to be released in both mating partners for pairing to occur. This inhibition occurs only between MTA and MTB of the same type (such as in the wild-type cell) but not between different types as in certain selfers. In this scheme, MTB plays a starting role in self/nonself-recognition to initiate cell pairing. Stable pairing can be produced only when both stable MTA–MTB interactions occur. How these similar proteins interact within and between cells and how they acquire their specificities through evolution are intriguing issues to further explore.

Selfing has been observed in some old vegetative stocks of *T*. *pigmentosa* [37]. In *Paramecium aurelia* and *T*. *rostrate*, cells undergo autogamy—the fusion of gamete-like nuclei in a single cell without mating—to generate new macronuclei under nutritional stress or during clonal senescence [38,39]. Thus, selfing may have evolved in some species to alleviate the problem of somatic aging. *T*. *thermophila* has not been known to display somatic clonal senescence. The selfing we have reported occurs throughout somatic life. Ku is evolutionary conserved and serves a role in repairing double-strand DNA breaks. If massive DNA damages occurs during macronuclear formation, it may occupy Ku for DNA repair and affect the *mat* locus rearrangement, as suggested by our Ku depletion results. In this way, perhaps Ku plays a role in sensing the accuracy of the massive DNA rearrangements during macronuclear formation. When stressed from imperfect rearrangements, selfing would occur to facilitate inbreeding and a new somatic life.

## Materials and methods

### Strains and cell culture

Inbred strains B2086 (mating type II), CU438 (Pmr/Pmr [mating type IV, pm-s]), CU427 (Chx/Chx [mating type VI, cy-s]), and CU428 (Mpr/Mpr [mating type VII, mp-s]) were obtained originally from Peter Bruns (Cornell University, Ithaca, NY). The mature tester strains of mating type III and mating type V were F1 progeny of CU427 and CU428. *TKU80* homozygous germline knockout strains were generated as described before [23]. Δ*TKU80* strains that lacked *TKU80* in both the germline and macronucleus were generated by mating 2 *TKU80* homozygous germline knockout strains at 30 °C. Mating pairs were isolated and incubated in SPP medium at 5–6 hours post-mixing. *TKU70-1*, *TKU70-2*, *TKU80*, and *GFP* hairpin RNA-generating strains were generated by using CU427 and CU428 as parental strains through the method described before [40]. Cells were cultured in axenic media as previously described [41].

### Selfing examination

Subcloned cells (approximately 2 × 10^6^ cells) were washed and starved in 10 mL of 10 mM Tris-HCl buffer (pH 7.4) at 30 °C. *Tetrahymena* mating pairs were fixed in phosphate-buffered saline (PBS) containing 2% paraformaldehyde at 6 hours after cells were starved. Mating pair ratio was examined under a microscope (Zeiss Axio Imager Z1; Carl Zeiss, Jena, Germany). For mass screening of selfers, subclones that had been grown to saturation in Neff medium were diluted 50-fold in 10 mM Tris-HCl buffer (pH 7.4) and incubated at 30 °C. Mating pairs were examined 12–24 hours after dilution. Mating pairs were viewed under a dissecting microscope (Leica MZ 125; Leica Microsystems GmbH, Wetzlar, Germany).

### *MTA*/*MTB* analysis

Whole cell DNA (approximately 95% was macronuclear DNA) was isolated for PCR analysis and Southern blot hybridization as previously described [42]. For PCR amplification of the *MTA* and *MTB* C-terminal junctions, we used 1 primer located on the *MTA* (or *MTB*) specific region and the other primer located on the constant region as described before [10]. For analysis by Southern hybridization, genomic DNA was digested by restriction enzymes and subjected to electrophoresis in a 0.8% agarose gel. DNA was transferred to a nylon membrane (IMMOBILON-NY+; Millipore, Bedford, MA) and hybridized with probes labeled with digoxigenin by a DIG High Prime DNA Labeling and Detection Starter Kit II (Roche). All primers used for the PCR reactions are listed in S5 Table. The membrane was washed first in 2× saline–sodium citrate (SSC) with 0.1% SDS and then in 0.5× SSC with 0.1% SDS at 65 °C several times. Luminescence signals was detected following manufacturer’s instructions.

### Hairpin RNAi gene silencing (knockdown)

To generate *TKU70-1*, *TKU70-2*, *TKU80*, and *GFP* knockdown constructs, the regions within the respective ORFs were amplified by PCR and cloned into the PCRII-I vector with 2 sets of restriction enzyme sites, producing 2 copies in inverted orientation, and then were moved into the pIBF rDNA vectors. The expression of the inverted dimer was driven by the cadmium-inducible *MTT1* promoter [40]. Each construct was transformed into mating *Tetrahymena* by electroporation using 10 μg of hairpin vector DNA [43]. Cells were transferred into SPP medium after electroporation (approximately 10 hours post-mixing) at 30 °C, followed by plating into 96-well plates (approximately 16 hours post-mixing). Transformants were selected in 120 μg/ml paromomycin (approximately 28 hours post-mixing). Silencing was induced using cadmium (1 μg/ml) at approximately 1 fission (approximately 25 hours post-mixing), approximately 16 fissions, and approximately 22 fissions. For continuously silencing of *TKU70-1*hp, *TKU70-2*hp, and *TKU80*hp, silencing were terminated at 69 fissions when cells were subcloned for selfing analysis after sexual maturation; continuous silencing of *GFP*, the proper control for normal cells, was terminated at 82 fissions when cells were subcloned for selfing analysis. The silencing effect was analyzed by quantitative real-time PCR (qRT-PCR). Total RNA was extracted from cells by using an RNA isolation kit (Roche). First-strand cDNA synthesis was performed using Transcriptor First Strand cDNA Synthesis Kit (Roche) and oligo (dT)18 as a primer. The qRT-PCR analysis was carried out by using the LightCycler Carousel-Based PCR system and LightCycler FastStart DNA Master^PLUS^ SYBR Green I (Roche). The relative expression levels were normalized by using α-tubulin mRNA as an internal control. Primer sequences are listed in S5 Table.

### Assortment assay

Single cells were subcloned into individual drops of medium on Petri dishes and kept in a humid box at 30 °C. One cell propagating to saturation in a drop requires approximately 13 fissions. At this time, a single cell was subcloned again to a fresh drop, and the remaining cells were replicated to medium with cycloheximide (25 μg/mL) or 6-methylpurine (15 μg/mL) for the analysis of drug resistance [44]. The drug-sensitive proportions were examined in serial vegetative fissions and analyzed by linear regression.

### Mating compatibility analysis

Tester cells of different mating types were starved in Dryl’s medium (Na citrate-2H_2_O (2 mM), NaH_2_PO_4_·H_2_O (1 mM), Na_2_HPO_4_ (1 mM), and CaCl_2_ (1.5 mM)) [45] at 30 °C for 5 hours and labeled with 40 μg/mL NHS-Rhodamine (Thermo Fisher Scientific) in Dryl’s medium for 2 hours. To avoid selfing, selfer strains were starved in 50 mM Tris-HCl (pH 7.4) at 30 °C for 7 hours. Before mixing, both selfer and tester cells were washed with 10 mM Tris-HCl (pH 7.4). Cells were collected at 4 hours post-mixing and fixed in PBS containing 2% paraformaldehyde, followed by DAPI staining (100 ng/mL). Images were captured using a fluorescent microscope (Zeiss Axio Imager Z1; Carl Zeiss, Jena, Germany). Unpaired single cells that showed meiotic nuclei were taken as indications of loose pairs that had initiated the mating reaction (including meiosis) but had separated prematurely. They were counted to determine the fraction of loose pairs.

### *MTA*/*MTB* expression analysis by RT-PCR

Total RNA was extracted from 3 hours’ starved cells by using an RNA isolation kit (Roche) and treated with DNAse followed by reverse transcription into cDNA using Transcriptor reverse transcriptase (Roche) with oligo (dT) primers. For examining the *MTAs* and *MTBs* expression, we used primers located on exons. The sequences of primers are used in RT-PCR are listed in S5 Table.

### IES elimination and chromosome breakage analysis

Genomic DNA was purified from cells in conjugation, feeding after conjugation, and vegetative growth. Deletion of IESs and chromosome breakage were examined by PCR analysis [46–48]. The primers used in these experiments are listed (S5 Table).

### Telomere-anchored PCR

Genomic DNA was extracted from vegetative cells collected at different fissions. Ten eliminated minichromosomes were examined by PCR analysis using a specific primer at 1 minichromosome end with a telomeric sequence primer [22]. The primers used in the experiment are listed in S5 Table.

## Supporting information

S1 Table *TKU80* mutation produced selfers. In the absence of *TKU80* in the micronucleus (germline knockout), cells could grow and mate to produce viable progeny (lacking *TKU80* in both nuclei). These progeny cells were subcloned, and their mating types examined after sexual maturation. About 50% of these subclones were selfers—cells within the subclone form mating pairs after starvation. Selfers are rare in the progeny of normal strains (CU427 and CU428).
(XLSX)

S2 Table Selfers can produce nonselfers (additional examples). Though *TKU80* selfers can stably maintained selfing phenotype, some of them can also produce nonselfing daughters of fixed mating types during vegetative growth. These nonselfers could be found by subcloning. Note that no new stabilized mating types were found in the later subcloning times.
(XLSX)

S3 Table *MTA*/*MTB* C-terminal exons of the selfers and their subclones. Selfers possessed multiple or mixed types of *MTA* and *MTB* complete C-terminal exons. In their subclones, nonselfers with fixed mating types were found, and their mating types were consistent with the detected *MTA*/*MTB* complete C-terminal exons in the selfers.
(XLSX)

S4 Table Silencing of *TKU70-2*/*TKU80* after conjugation produced selfers. Silencing of each gene was induced at different time periods after conjugation: at approximately 1, 16, and 22 fissions continuously, or temporary (1–22 fissions). Selfers were produced after silencing of *TKU70-2* or *TKU80* during vegetative growth.
(XLSX)

S5 Table List of oligonucleotide DNA. The primers used in each experiment (bold text) are listed. The predicted PCR product length amplified from Mic, Mac, and the length of IESs within the Mic are indicated. IES, internal eliminated sequence; Mac, macronucleus; Mic, micronucleus.
(XLSX)

S1 Fig Selfer could form mating pairs at high efficiencies after starvation. A sexually matured selfer was starved (2×10^5^ cells/mL), and the intraclonal mating pairs were counted at different time points after starvation. *n* ≥ 200. The data underlying this figure can be found in S1 Data.
(TIF)

S2 Fig Selfing of Δ*TKU80* cells were arrested at late conjugation stage. The conjugation of selfers also generated the new macronuclei, but they were degraded eventually at late stage. Arrows and arrowheads indicate new micronuclei and new macronuclei, respectively. Pm, parental macronucleus.
(TIF)

S3 Fig Silencing of *TKU70-2* and *TKU80* by inducing hairpin RNA in *Tetrahymena*. Expression of *TKU70-2* and *TKU80* were dramatically reduced in *TKU70-2* and *TKU80* knockdown cells, as measured by qRT-PCR. *n* = 12. The data underlying this figure can be found in S1 Data. qRT-PCR, quantitative real-time polymerase chain reaction.
(TIF)

S4 Fig Mating compatibility analysis using NHS-Rhodamine labeling. Tester cells was labeled by NHS-Rhodamine (upper panel). After starvation, Δ*TKU80* selfer E4 was mixed with the NHS-Rhodamine labeled tester CU427 (lower panel). Nonselfing pair (E4×CU427, right) and selfing pair (E4×E4, left) are shown. Scale bar = 10 μm. DIC, differential interference contrast.
(TIF)

S5 Fig Mating compatibility between selfers and normal cells at different mixing ratios. As in Fig 6, tester cells of 6 different MTs were labeled with NHS-Rhodamine. Starved selfer k4 (containing both MT IV and MT VI genes, upper panels) and selfer E4 (containing II+V mixed-type genes, lower panels) were mixed with tester cells at the ratios of 10:1, 1:1, and 1:10. Selfing and/or nonselfing (pairing with tester) pairs formed after 4 hours post-mixing were counted under a microscope. *n* = 100 pairs. The data underlying this figure can be found in S1 Data. MT, mating type.
(TIF)

S6 Fig Multiple *MTA*/*MTB* genes expressed in Δ*TKU80* cells. Expression of *MTA*/*MTB* genes after 3 hours’ starvation was examined in control cells and Δ*TKU80* selfers by RT-PCR. The open rectangles and black arrows indicate exons and PCR primers, respectively (left panels). The RT-PCR results are shown in right panels. Multiple mating-type gene transcripts were detected in the selfers. RT+ and RT- indicate with or without reverse transcriptase for sample preparation. The amplified cDNA and genomic DNA are indicated by black and open arrowheads, respectively. Raw images associated with this figure can be found in S1 Raw Images. RT-PCR, reverse transcription polymerase chain reaction.
(TIF)

S7 Fig Most events of programmed global DNA rearrangements occurred normally in Δ*TKU80* cells. (A) *TPB*1-dependent IES deletions. Schematic diagrams on left panels illustrate PCR-based assay on *TPB*1-dependent IES deletions using primers (arrows) flanking each IES (gray box). Deletion of 4 *TPB1-*dependent IESs (E, G, N, and O elements) was not affected after conjugation of *TKU80* germline knockout cells (24 hour stage of conjugation) or in their progeny pools (at 4 and 22 fissions or fissions after conjugation) and 4 selfer strains. The progeny cells of *TPB1* knockdown mating (*TPB1*-KD) served as the control for *TPB1-*dependent IESs deletion failure. The open and black arrowheads indicate the expected micronuclear and macronuclear form products. (B) *TPB2-*depedent IESs deletion. Schematic representations of the assay for *TPB2-*depedent IESs deletions are shown in the left panels. Arrows represent the PCR primers used in the assay. Deletion of M element (light and dark gray boxes) generates 2 alternative junctions, an approximately 0.9-kb or an approximately 0.6-kb deletion (dark gray) in the macronucleus. Deletion of Tlr1 and rdn element generates variable junctions. Deletion of 5 *TPB2-*dependent IESs (M, R, cam, Tlr1, and rdn elements) occurred normally in the conjugation of *TKU80* germline knockout cells (24 h) and Δ*TKU80* progeny cells (4F, 22F, and selfers). The open arrowheads indicate the expected PCR product of the micronuclear form. The solid arrowheads and the bracket indicate the PCR products of the macronuclear forms. (C) DNA fragmentation coupled with de novo telomere (gray bar) addition at chromosome breakages site (gray dot box). These sites were analyzed by PCR using telomere sequence as one of the primers (left panel). Chromosome breakages were not affected in conjugation of *TKU80* germline knockout cells (24 hours) and Δ*TKU80* cells (4F, 22F, and selfers) at the 4 Cbs sites (Cbs819, Cbs826, Cbs4L-2, and Cbs5-2). The open and black arrowheads indicate the micronuclear and macronuclear form products. Raw images associated with this figure can be found in S1 Raw Images. Conj, conjugation; F, number of fissions; IES, internal eliminated sequence.
(TIF)

S8 Fig The *mat* rearranged intermediates were observed in selfers generated by *TKU80* knockdown. The *mat* rearranged intermediates were examined by PCR in selfers that generated by inducing *TKU80* hairpin silencing within 16 fissions after conjugation. These selfer was examined after sex matured, and DNA was collected at approximately 120 fissions (a and b are pool DNA of 10 selfers). PCR primers for detection of the intermediates are indicated by blue arrows in left panel. Raw images associated with this figure can be found in S1 Raw Images.
(TIF)

S9 Fig A model of possible *mat* rearrangements in *Tetrahymena*. One possible way with 2 steps of the complex *mat* rearrangements is illustrated (modified from the previous model [10,18]). The first step is to delete all the IESs in *mat* locus of the new developing macronucleus. The blue connecting lines indicate the deletion of IESs (gray rectangles, upper panel). The second step includes several recombinations between intrachromosomal *MTA/MTB* C-terminal homologous exon sequences (gray connecting lines, lower panel). The unusual products, or intermediates, could be formed through this step. In the absence of Ku, multiple normal *mat* genes and/or the rearrangement intermediates may be retained in the macronucleus and produce selfers.
(TIF)

S10 Fig Examination of minichromosome elimination in Δ*TKU80* cells. The telomere-anchored PCR results are shown for 10 minichromosomes at different fissions after conjugation. They were generated and removed normally in Δ*TKU80* cells as in normal cells, suggesting that *TKU80* is not involved in the programmed minichromosome elimination process. Raw images associated with this figure can be found in S1 Raw Images.
(TIF)

S1 Data Numerical raw data. The Excel file contains separate spreadsheets of the numerical raw data of the graph in Figs 4, 5 and 6, S1, S3 and S5 Figs.
(XLSX)

S1 Raw Images Original blot and gel data. The PDF file contains all the original blot and gel data in Figs 2 and 3, S6, S7, S8 and S10 Figs.
(PDF)

## Abbreviations

IES: internal eliminated sequence
NHEJ: nonhomologous end joining
rDNA: ribosomal RNA gene
RNAi: RNA-induced gene silencing
RT-PCR: reverse transcription polymerase chain reaction
