# Supplementary figures and images for: Selfing mutants link Ku proteins to mating type determination in Tetrahymena
Source: PLoS Biol. 2020 Aug 3;18(8):e3000756. doi: 10.1371/journal.pbio.3000756 (PMC7398496; doi:10.1371/journal.pbio.3000756)

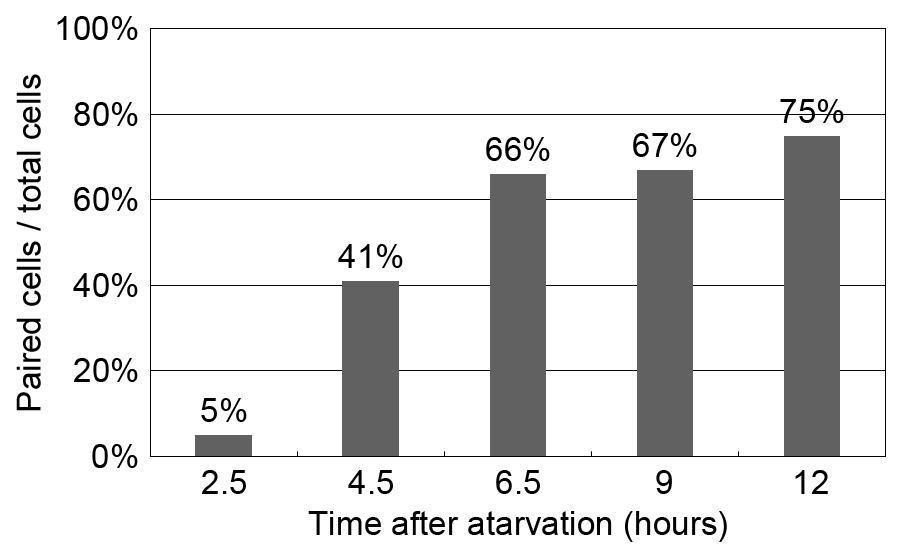

Supplement: S1 Fig — A sexually matured selfer was starved (2×10^5^ cells/mL), and the intraclonal mating pairs were counted at different time points after starvation. n ≥ 200. The data underlying this figure can be found in S1 Data. (TIF) [file pbio.3000756.s006.tif]

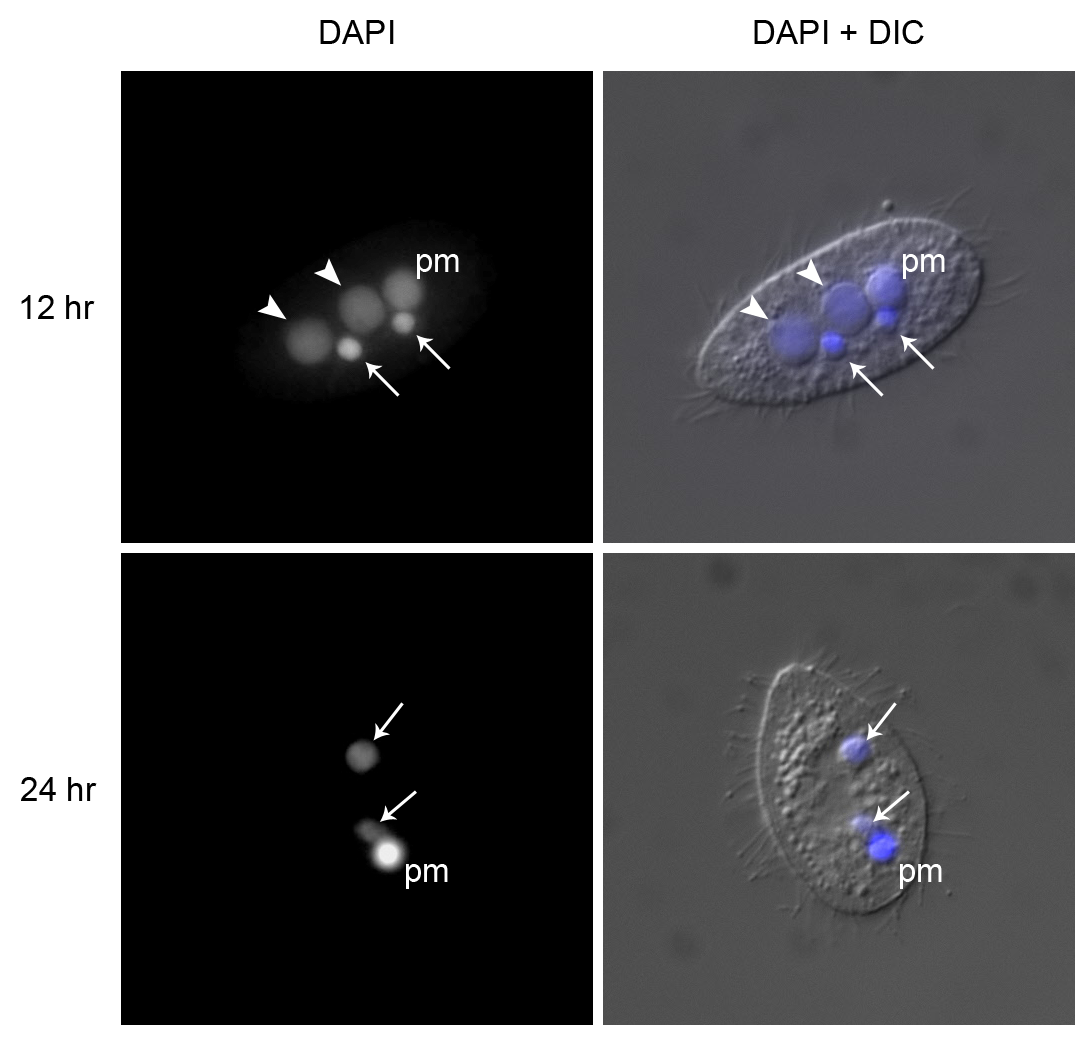

Supplement: S2 Fig — The conjugation of selfers also generated the new macronuclei, but they were degraded eventually at late stage. Arrows and arrowheads indicate new micronuclei and new macronuclei, respectively. Pm, parental macronucleus. (TIF) [file pbio.3000756.s007.tif]

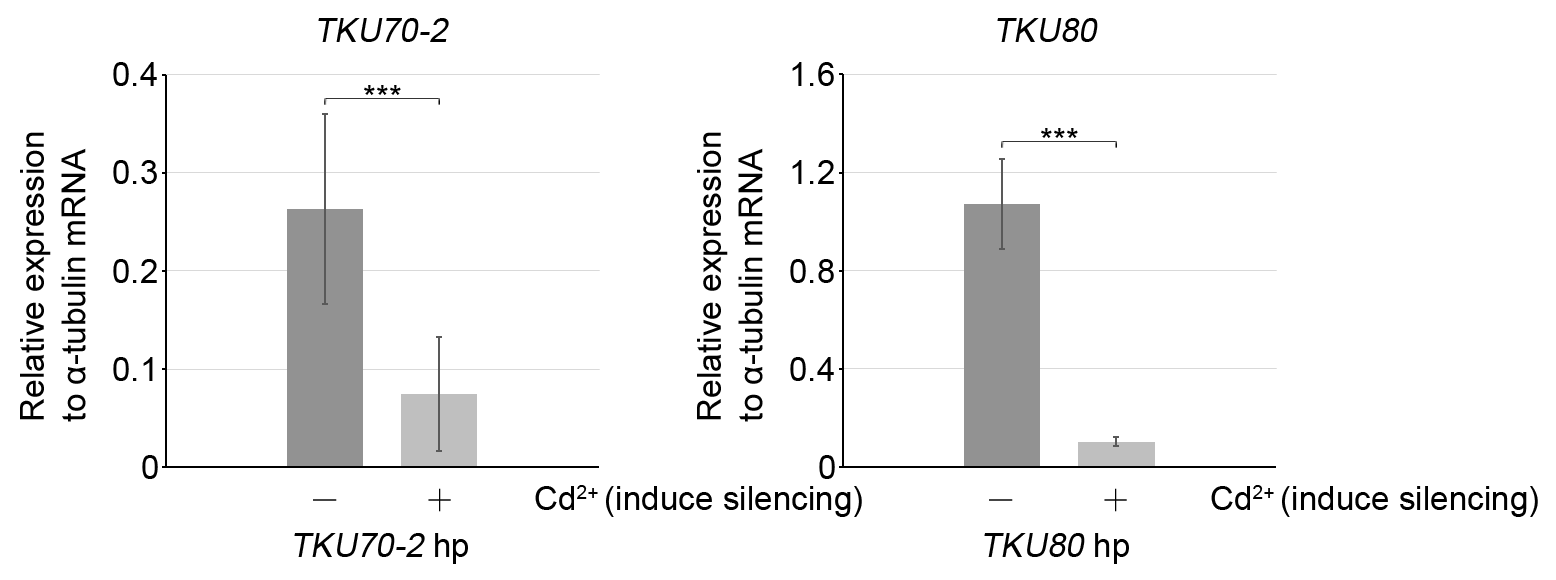

Supplement: S3 Fig — Expression of TKU70-2 and TKU80 were dramatically reduced in TKU70-2 and TKU80 knockdown cells, as measured by qRT-PCR. n = 12. The data underlying this figure can be found in S1 Data. qRT-PCR, quantitative real-time polymerase chain reaction. (TIF) [file pbio.3000756.s008.tif]

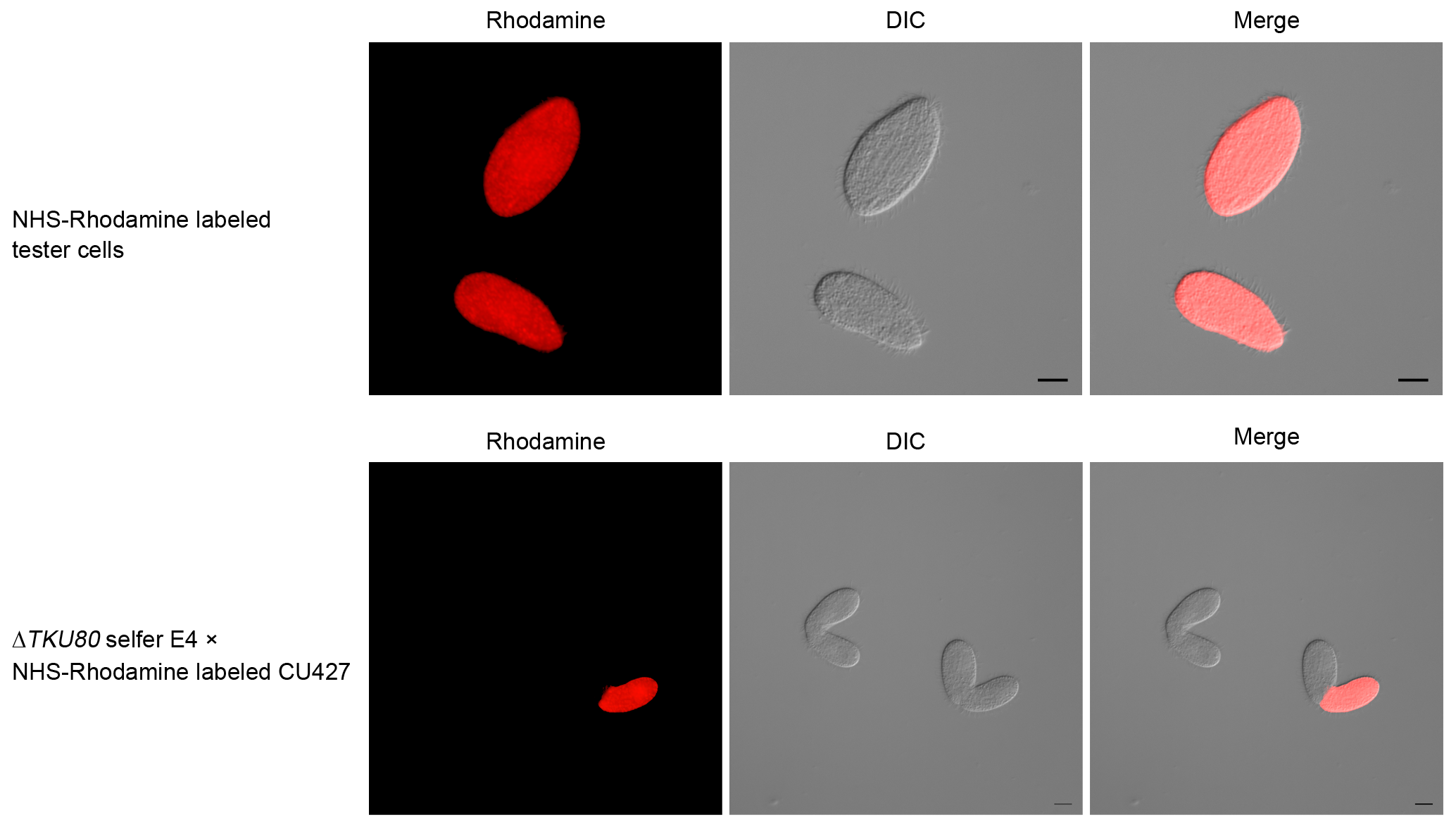

Supplement: S4 Fig — Tester cells was labeled by NHS-Rhodamine (upper panel). After starvation, ΔTKU80 selfer E4 was mixed with the NHS-Rhodamine labeled tester CU427 (lower panel). Nonselfing pair (E4×CU427, right) and selfing pair (E4×E4, left) are shown. Scale bar = 10 μm. DIC, differential interference contrast. (TIF) [file pbio.3000756.s009.tif]

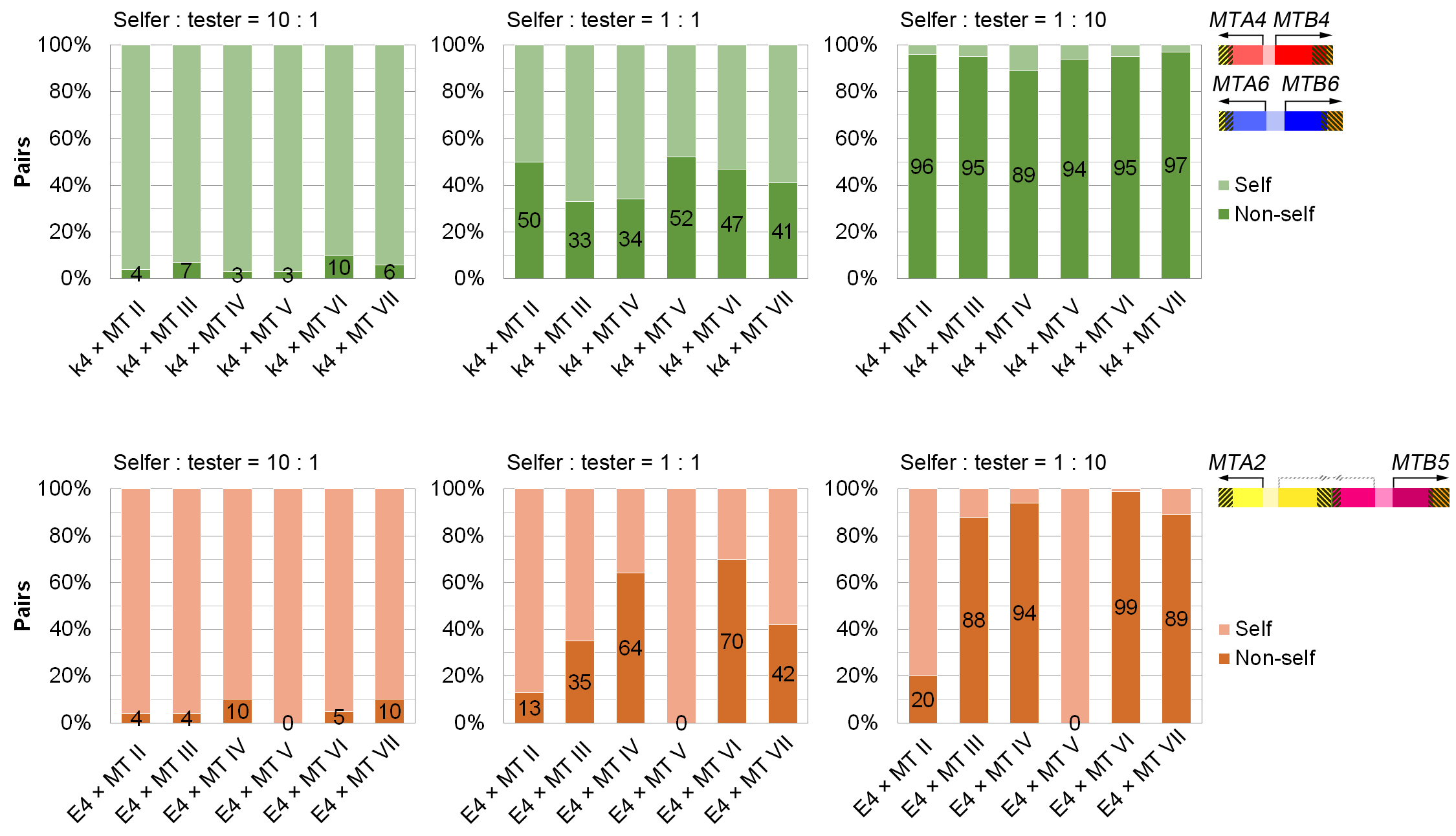

Supplement: S5 Fig — As in Fig 6, tester cells of 6 different MTs were labeled with NHS-Rhodamine. Starved selfer k4 (containing both MT IV and MT VI genes, upper panels) and selfer E4 (containing II+V mixed-type genes, lower panels) were mixed with tester cells at the ratios of 10:1, 1:1, and 1:10. Selfing and/or nonselfing (pairing with tester) pairs formed after 4 hours post-mixing were counted under a microscope. n = 100 pairs. The data underlying this figure can be found in S1 Data. MT, mating type. (TIF) [file pbio.3000756.s010.tif]

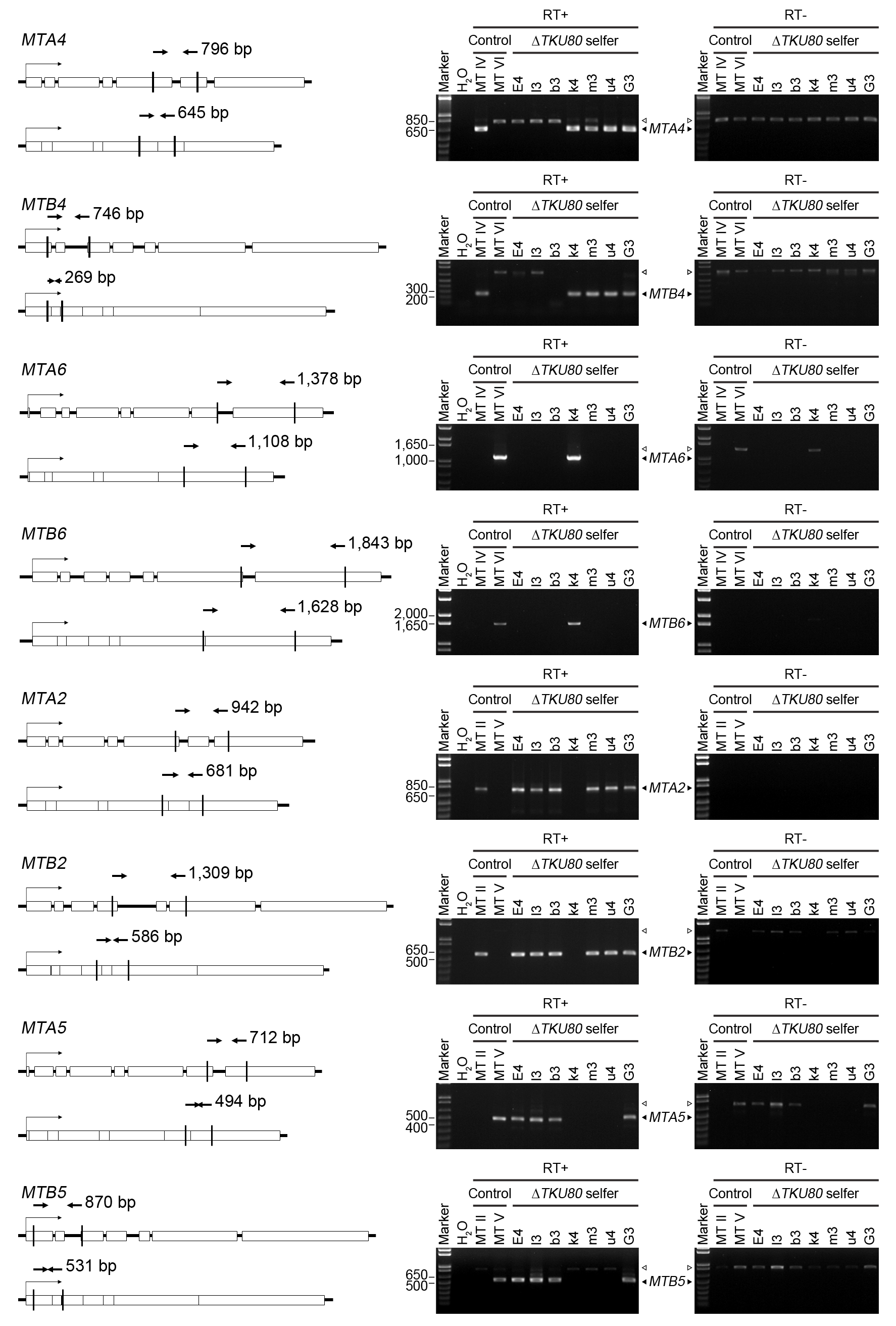

Supplement: S6 Fig — Expression of MTA/MTB genes after 3 hours’ starvation was examined in control cells and ΔTKU80 selfers by RT-PCR. The open rectangles and black arrows indicate exons and PCR primers, respectively (left panels). The RT-PCR results are shown in right panels. Multiple mating-type gene transcripts were detected in the selfers. RT+ and RT- indicate with or without reverse transcriptase for sample preparation. The amplified cDNA and genomic DNA are indicated by black and open arrowheads, respectively. Raw images associated with this figure can be found in S1 Raw Images. RT-PCR, reverse transcription polymerase chain reaction. (TIF) [file pbio.3000756.s011.tif]

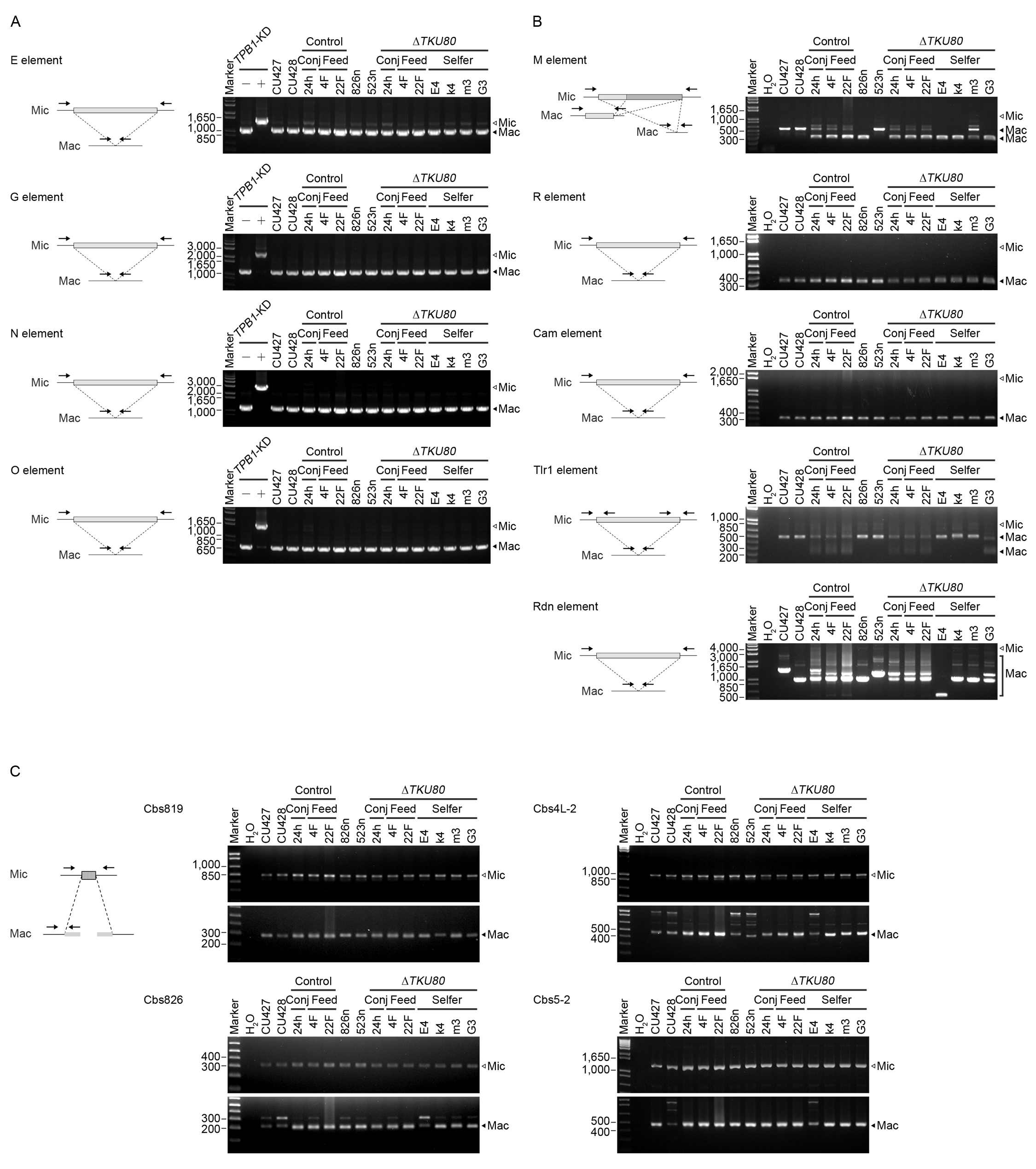

Supplement: S7 Fig — (A) TPB1-dependent IES deletions. Schematic diagrams on left panels illustrate PCR-based assay on TPB1-dependent IES deletions using primers (arrows) flanking each IES (gray box). Deletion of 4 TPB1-dependent IESs (E, G, N, and O elements) was not affected after conjugation of TKU80 germline knockout cells (24 hour stage of conjugation) or in their progeny pools (at 4 and 22 fissions or fissions after conjugation) and 4 selfer strains. The progeny cells of TPB1 knockdown mating (TPB1-KD) served as the control for TPB1-dependent IESs deletion failure. The open and black arrowheads indicate the expected micronuclear and macronuclear form products. (B) TPB2-depedent IESs deletion. Schematic representations of the assay for TPB2-depedent IESs deletions are shown in the left panels. Arrows represent the PCR primers used in the assay. Deletion of M element (light and dark gray boxes) generates 2 alternative junctions, an approximately 0.9-kb or an approximately 0.6-kb deletion (dark gray) in the macronucleus. Deletion of Tlr1 and rdn element generates variable junctions. Deletion of 5 TPB2-dependent IESs (M, R, cam, Tlr1, and rdn elements) occurred normally in the conjugation of TKU80 germline knockout cells (24 h) and ΔTKU80 progeny cells (4F, 22F, and selfers). The open arrowheads indicate the expected PCR product of the micronuclear form. The solid arrowheads and the bracket indicate the PCR products of the macronuclear forms. (C) DNA fragmentation coupled with de novo telomere (gray bar) addition at chromosome breakages site (gray dot box). These sites were analyzed by PCR using telomere sequence as one of the primers (left panel). Chromosome breakages were not affected in conjugation of TKU80 germline knockout cells (24 hours) and ΔTKU80 cells (4F, 22F, and selfers) at the 4 Cbs sites (Cbs819, Cbs826, Cbs4L-2, and Cbs5-2). The open and black arrowheads indicate the micronuclear and macronuclear form products. Raw images associated with this figure can be found in S1 [file pbio.3000756.s012.tif]

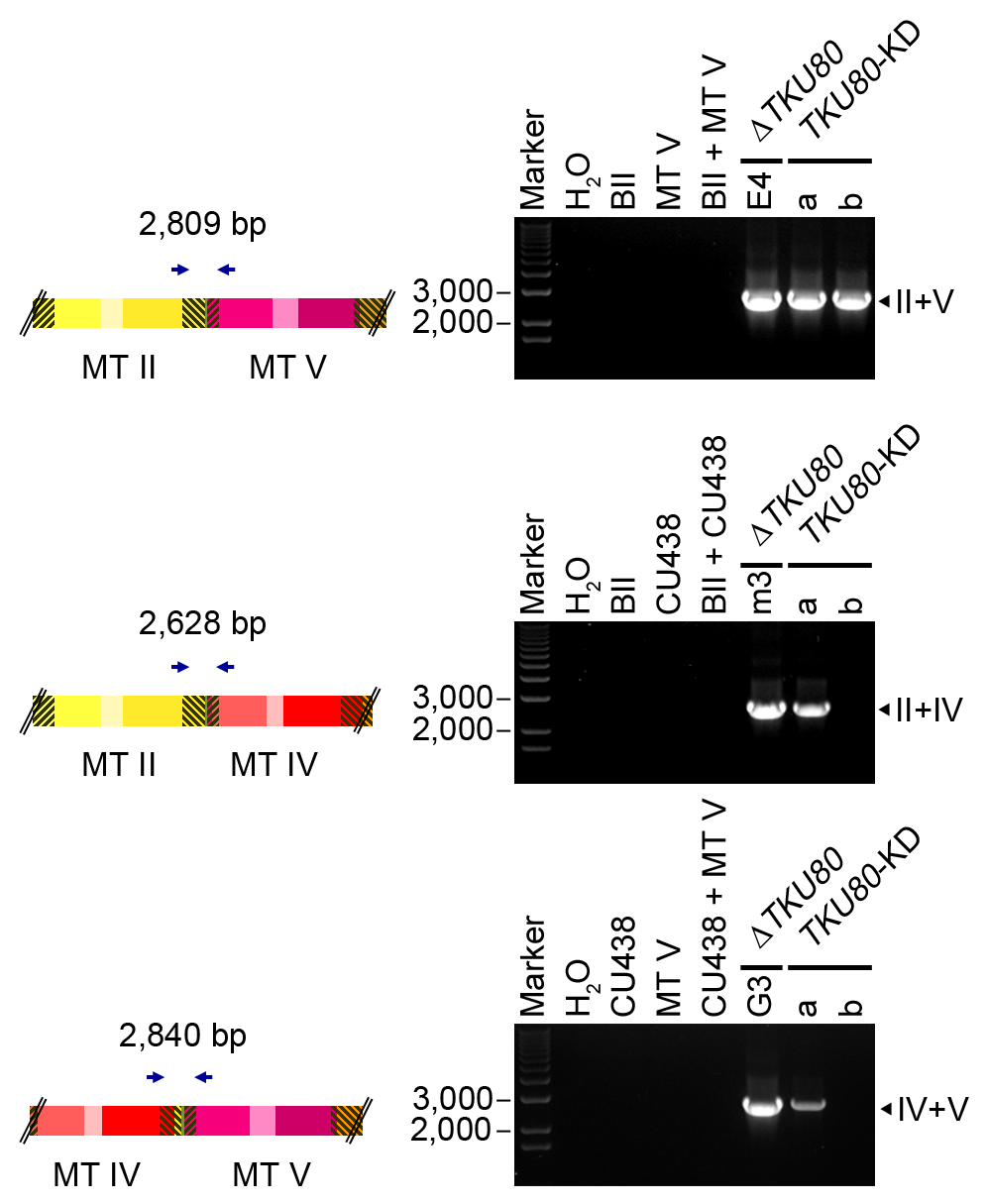

Supplement: S8 Fig — The mat rearranged intermediates were examined by PCR in selfers that generated by inducing TKU80 hairpin silencing within 16 fissions after conjugation. These selfer was examined after sex matured, and DNA was collected at approximately 120 fissions (a and b are pool DNA of 10 selfers). PCR primers for detection of the intermediates are indicated by blue arrows in left panel. Raw images associated with this figure can be found in S1 Raw Images. (TIF) [file pbio.3000756.s013.tif]

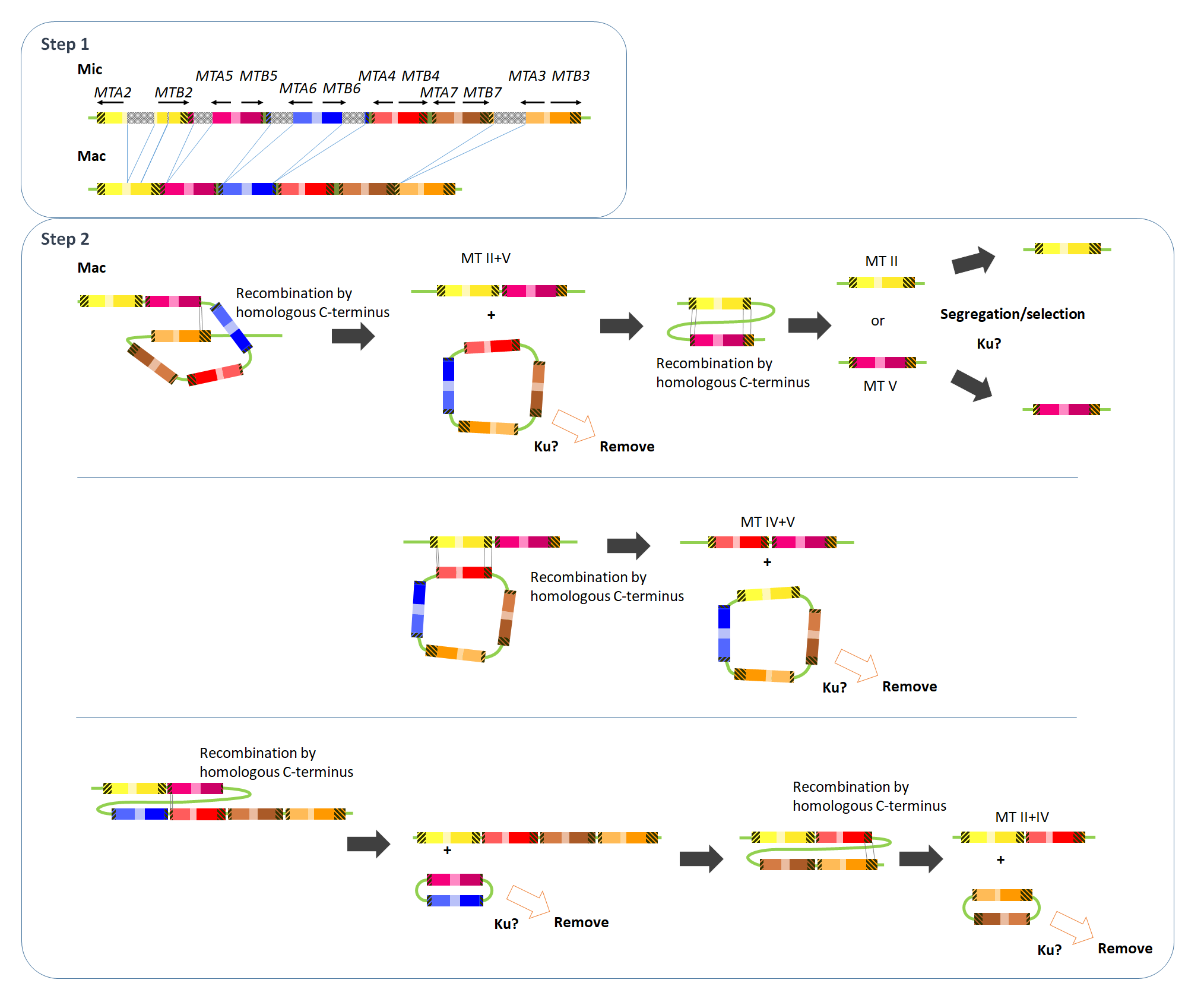

Supplement: S9 Fig — One possible way with 2 steps of the complex mat rearrangements is illustrated (modified from the previous model [10,18]). The first step is to delete all the IESs in mat locus of the new developing macronucleus. The blue connecting lines indicate the deletion of IESs (gray rectangles, upper panel). The second step includes several recombinations between intrachromosomal MTA/MTB C-terminal homologous exon sequences (gray connecting lines, lower panel). The unusual products, or intermediates, could be formed through this step. In the absence of Ku, multiple normal mat genes and/or the rearrangement intermediates may be retained in the macronucleus and produce selfers. (TIF) [file pbio.3000756.s014.tif]

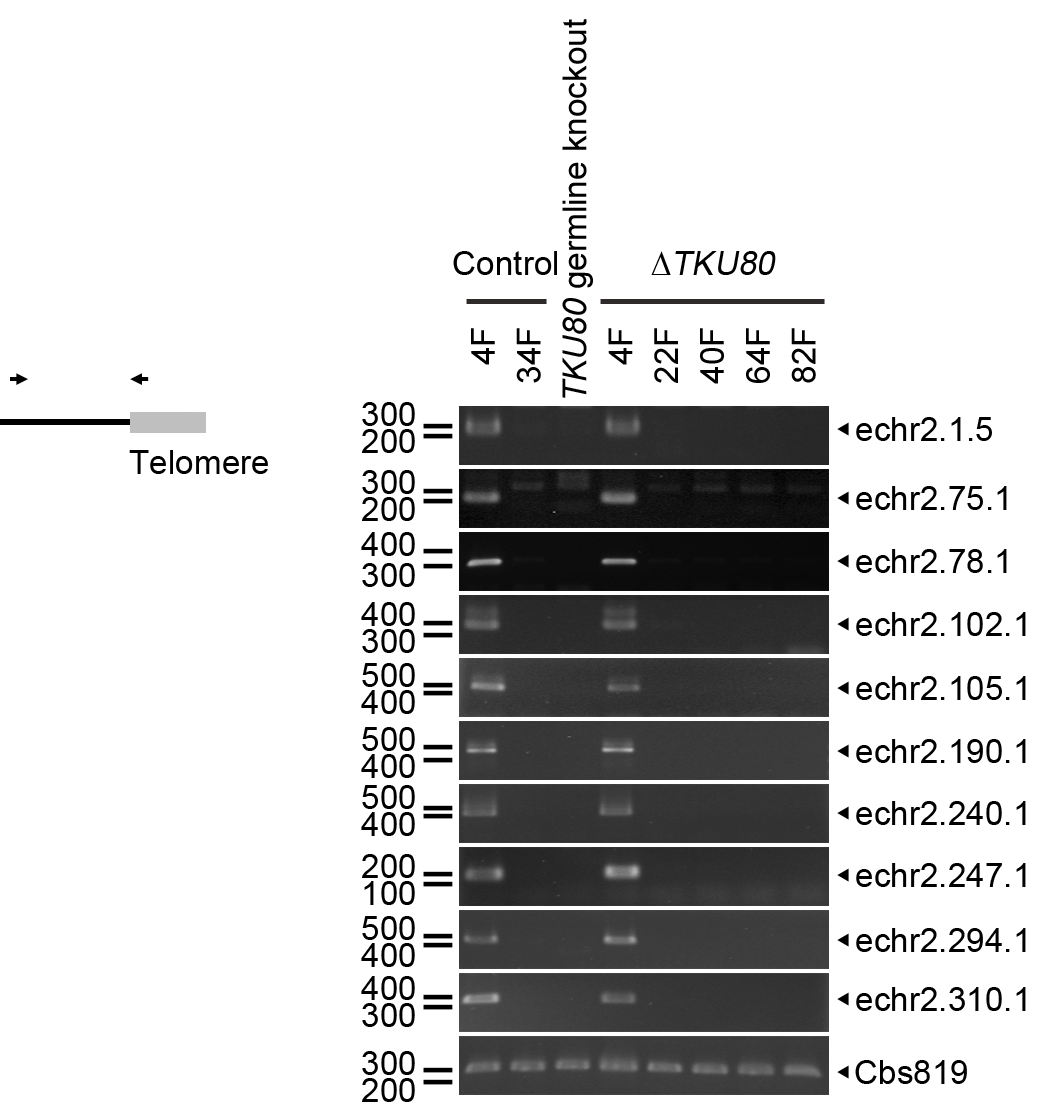

Supplement: S10 Fig — The telomere-anchored PCR results are shown for 10 minichromosomes at different fissions after conjugation. They were generated and removed normally in ΔTKU80 cells as in normal cells, suggesting that TKU80 is not involved in the programmed minichromosome elimination process. Raw images associated with this figure can be found in S1 Raw Images. (TIF) [file pbio.3000756.s015.tif]
