# Supplementary material for: Selfing mutants link Ku proteins to mating type determination in Tetrahymena
Source: PLoS Biol. 2020 Aug 3;18(8):e3000756. doi: 10.1371/journal.pbio.3000756 (PMC7398496; doi:10.1371/journal.pbio.3000756)
Supplement: S1 Raw Images — The PDF file contains all the original blot and gel data in Figs 2 and 3, S6, S7, S8 and S10 Figs. (PDF) [file pbio.3000756.s017.pdf]

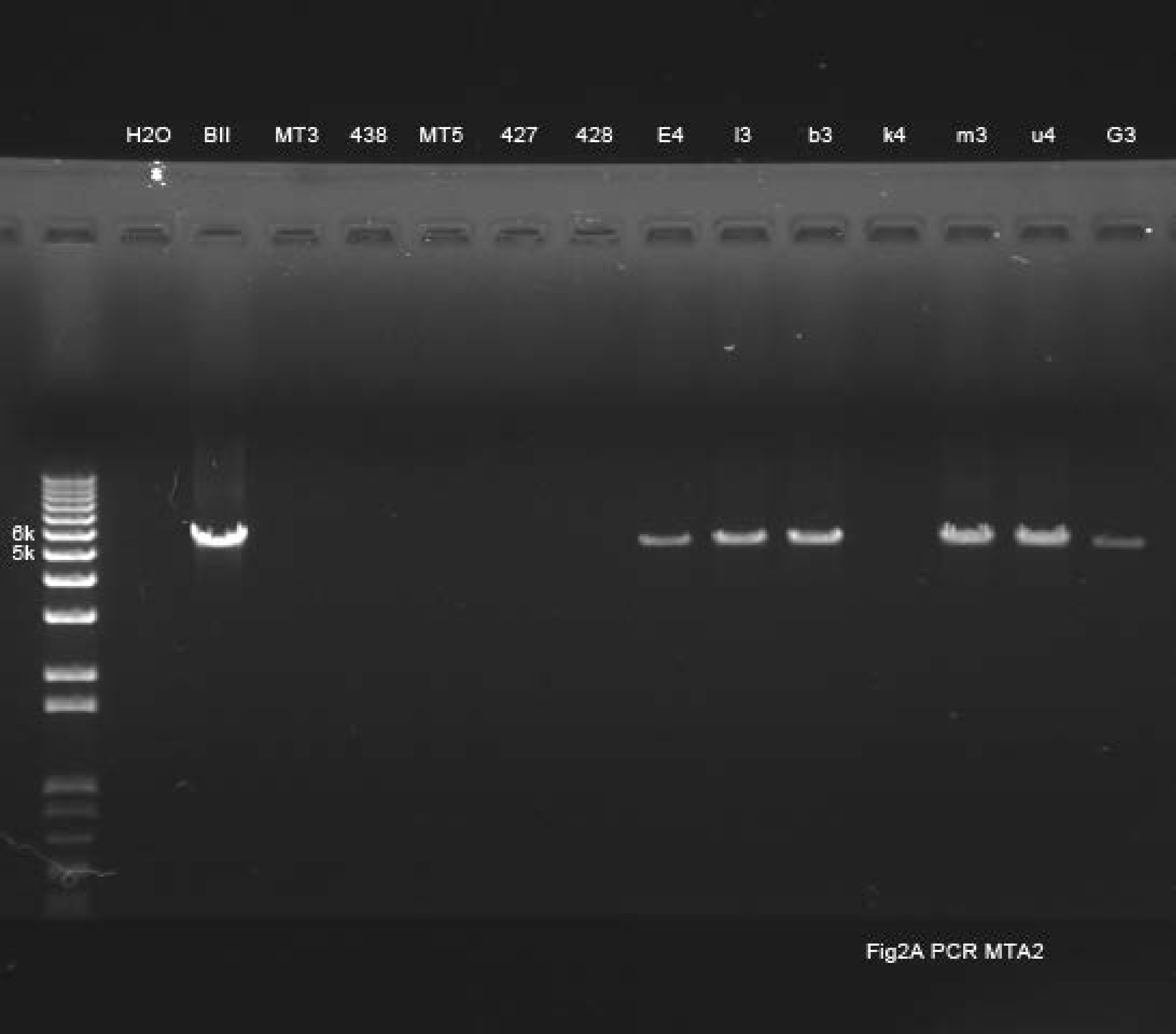
H2O
BII
MT3
438
MT5
427
428
E4
I3
b3
k4
m3
u4
G3
6k
5k
Fig2A PCR MTA2

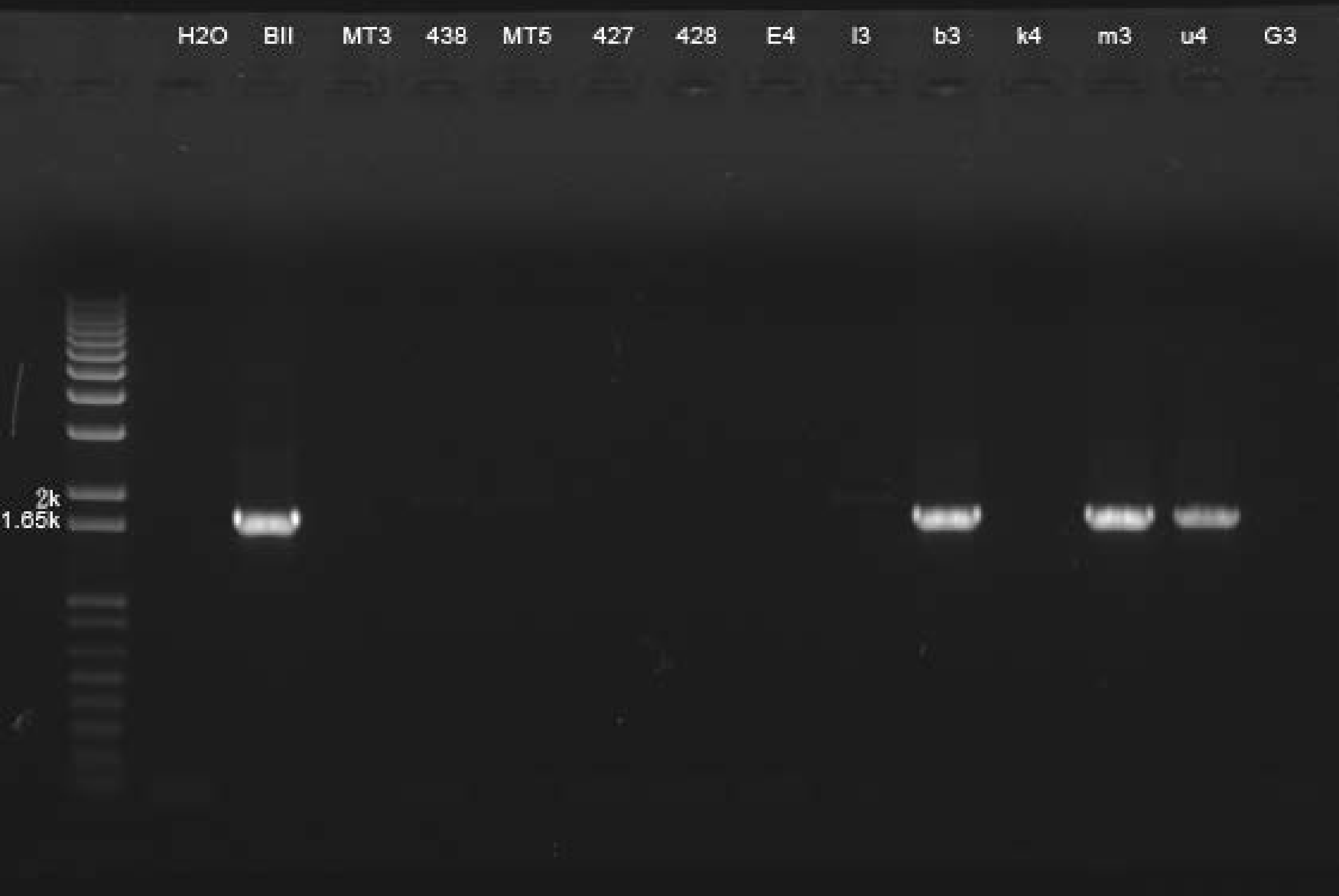

Fig2A PCR MTB2

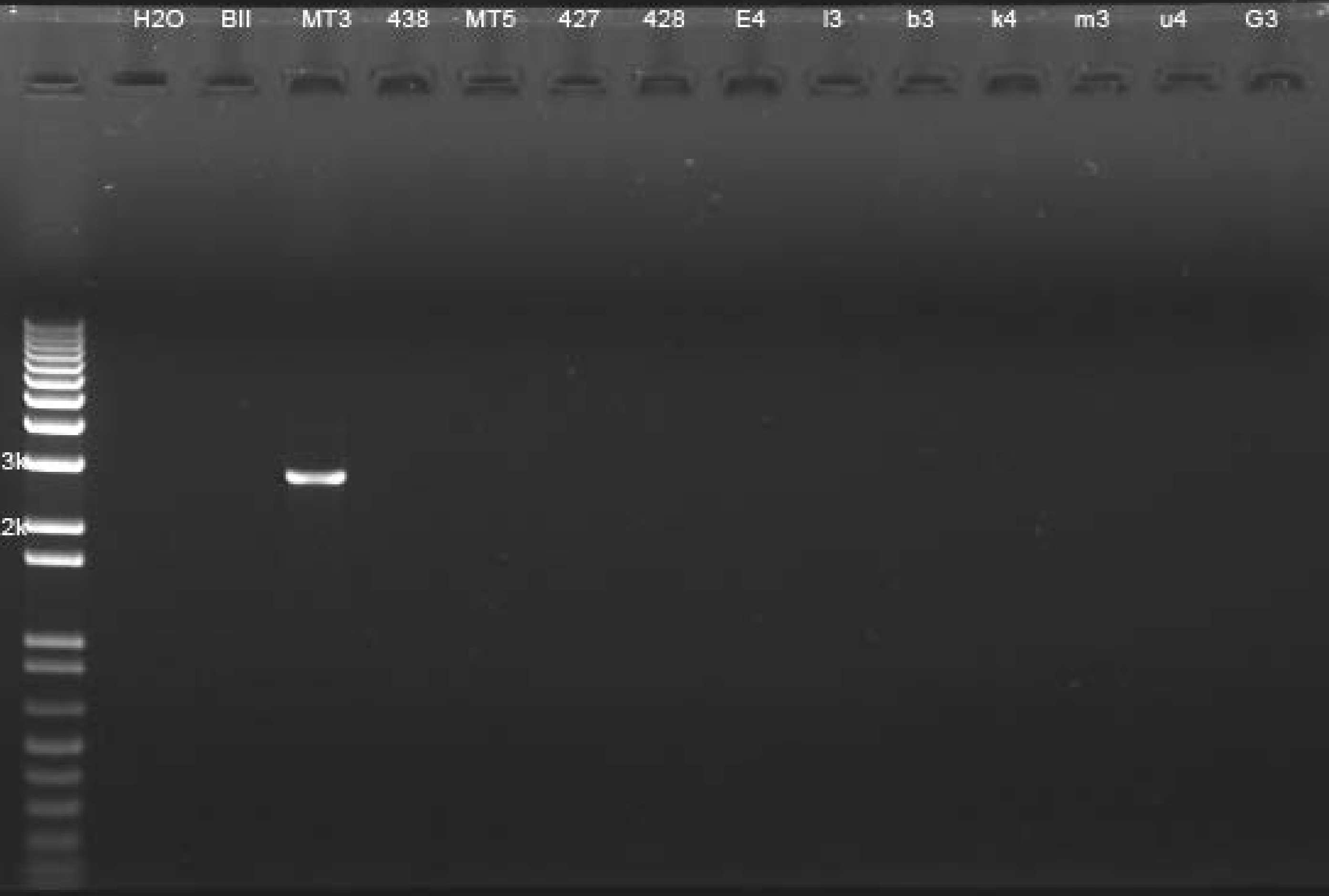

Fig2A PCR MTA3

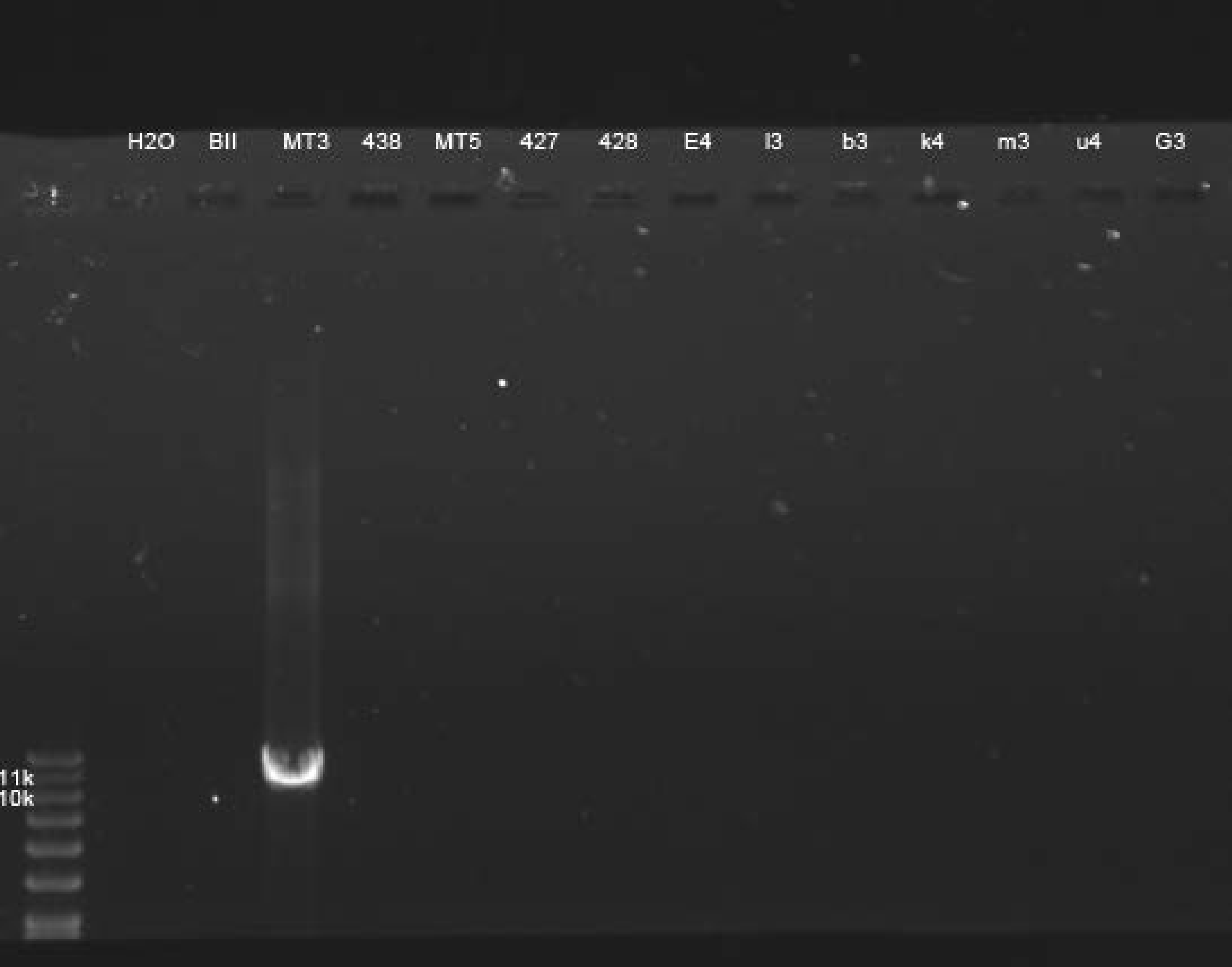

Fig2A PCR MTB3

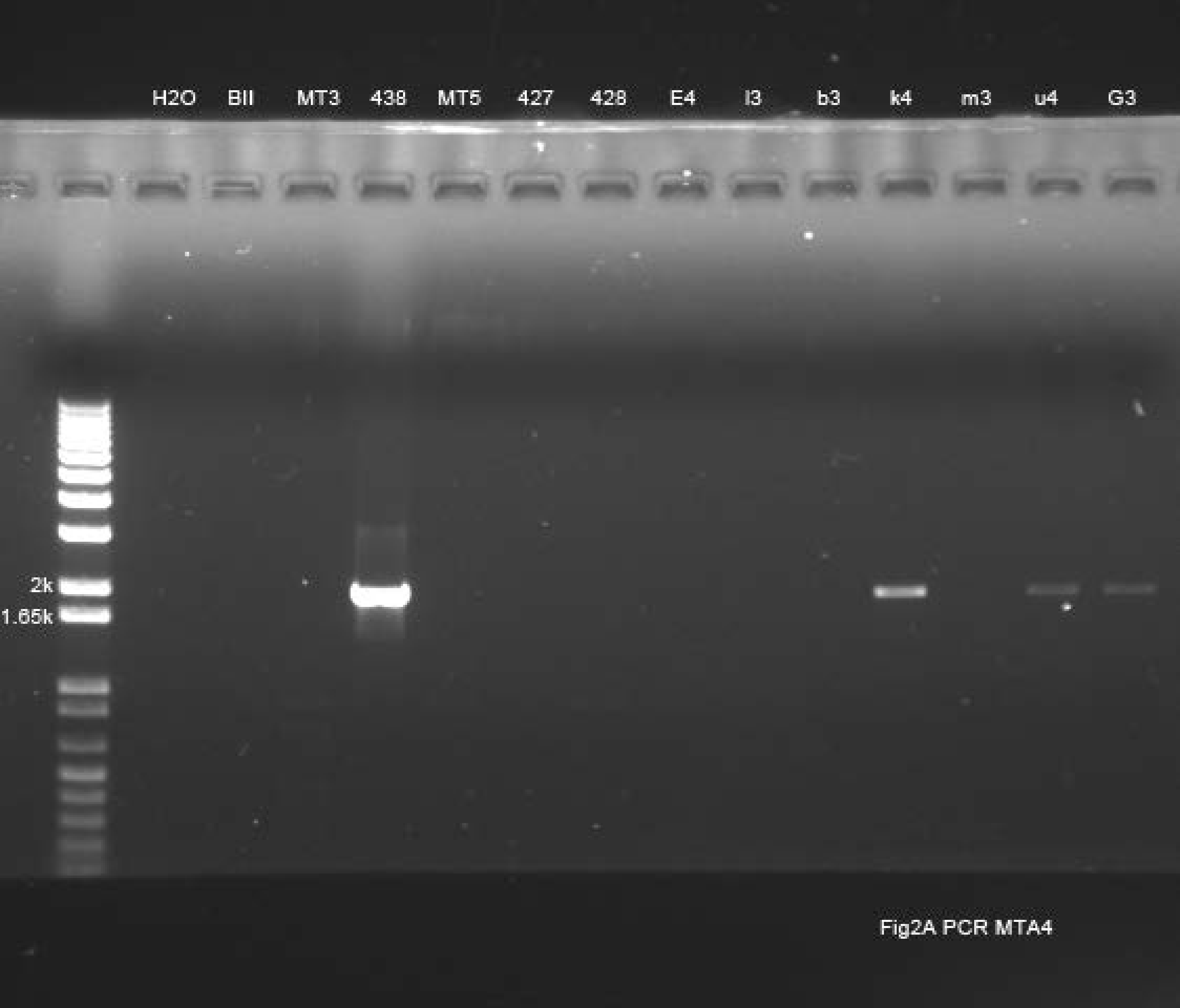
H2O
BII
MT3
438
MT5
427
428
E4
I3
b3
k4
m3
u4
G3
2k
1.65k
Fig2A PCR MTA4

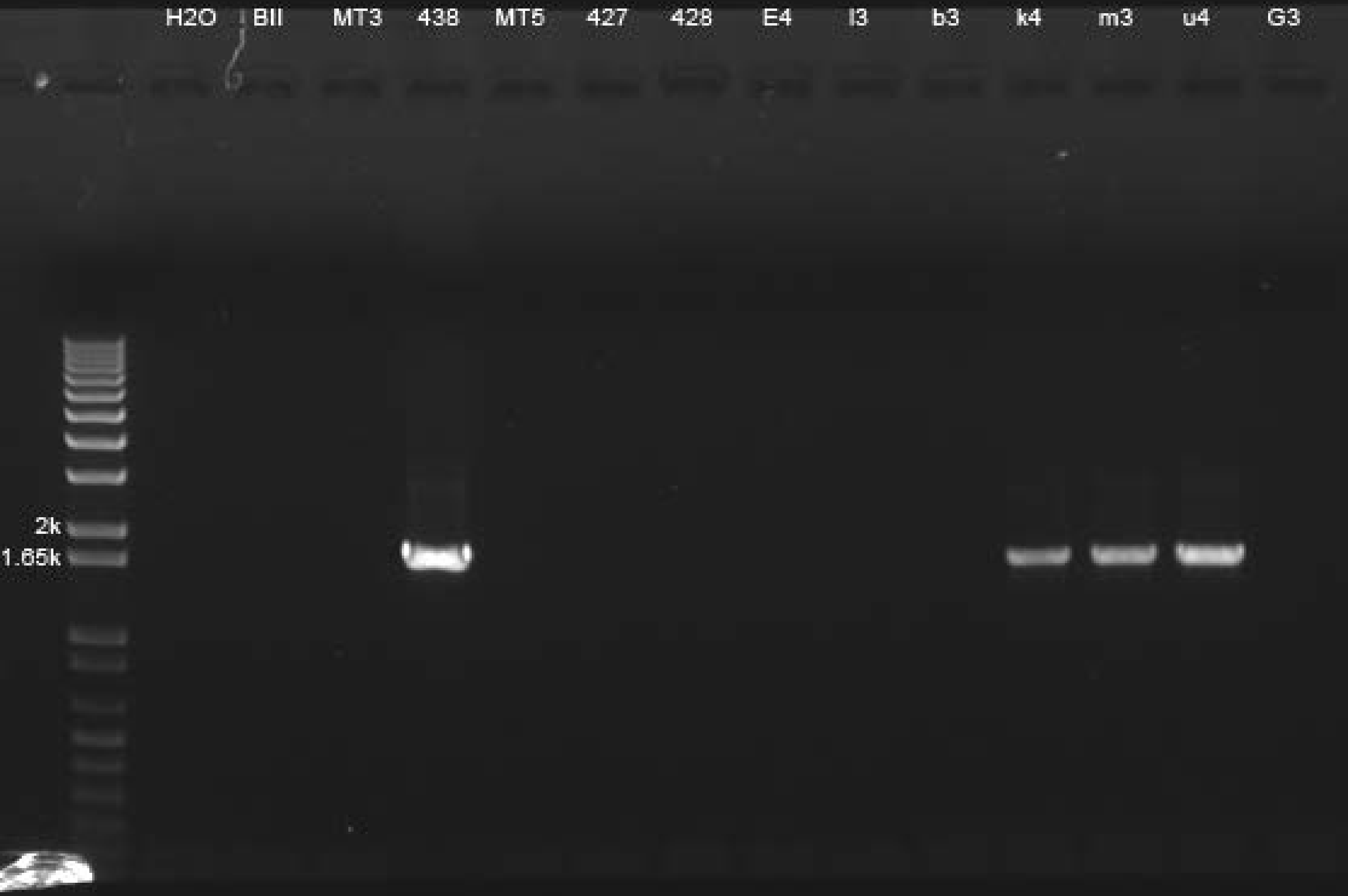

Fig2A PCR MTB4

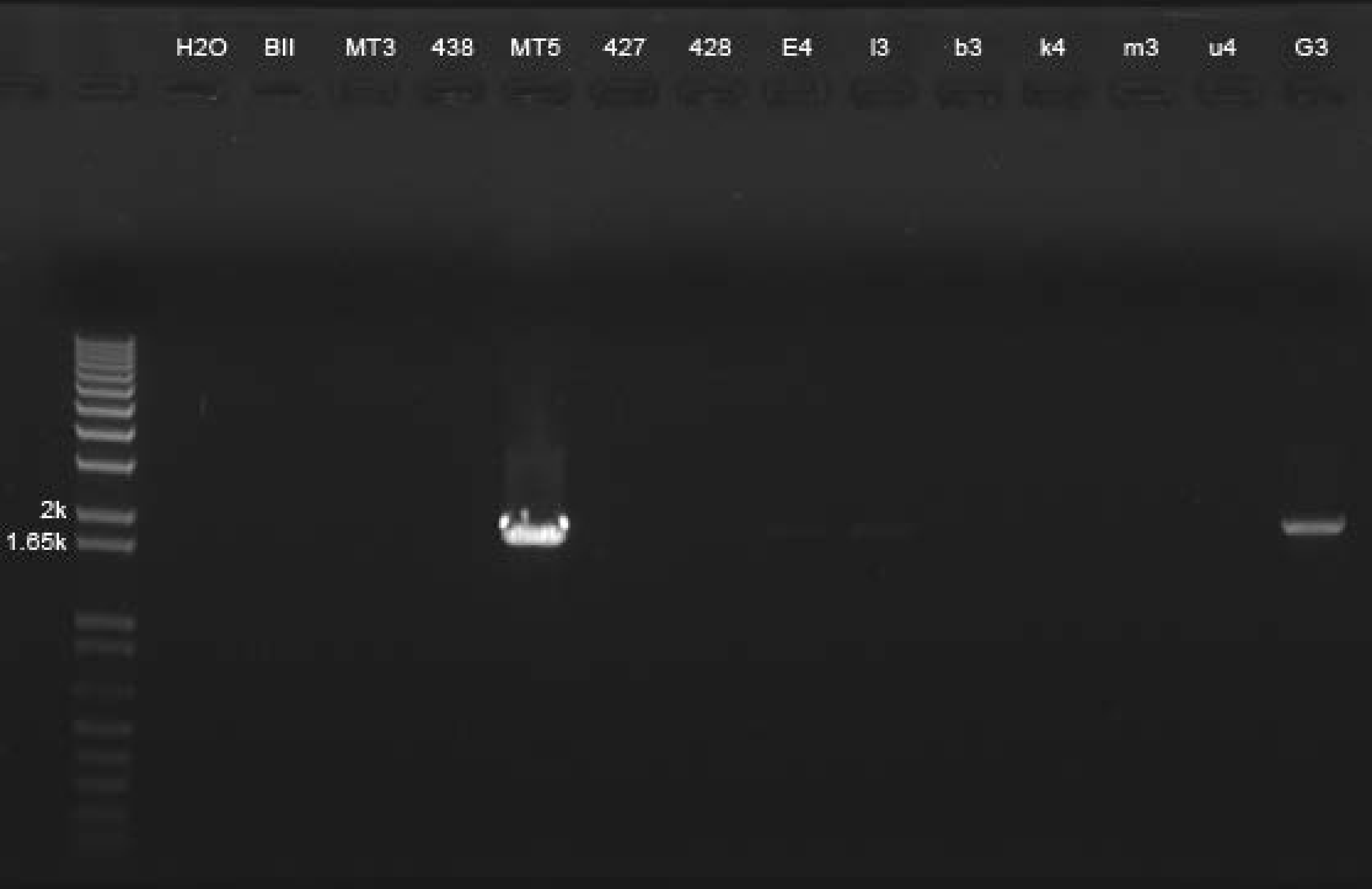

Fig2A PCR MTA5

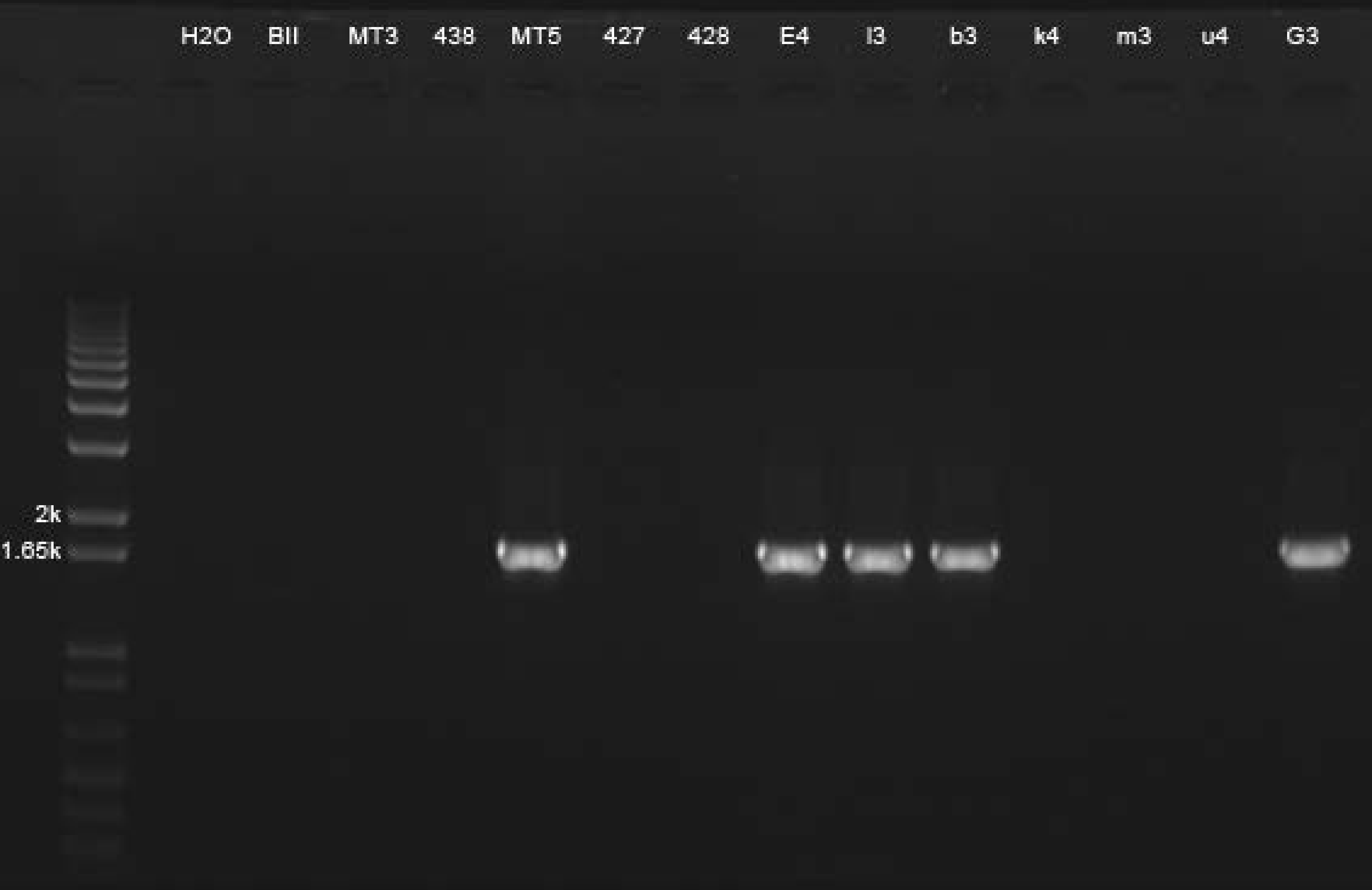

Fig2A PCR MTB5

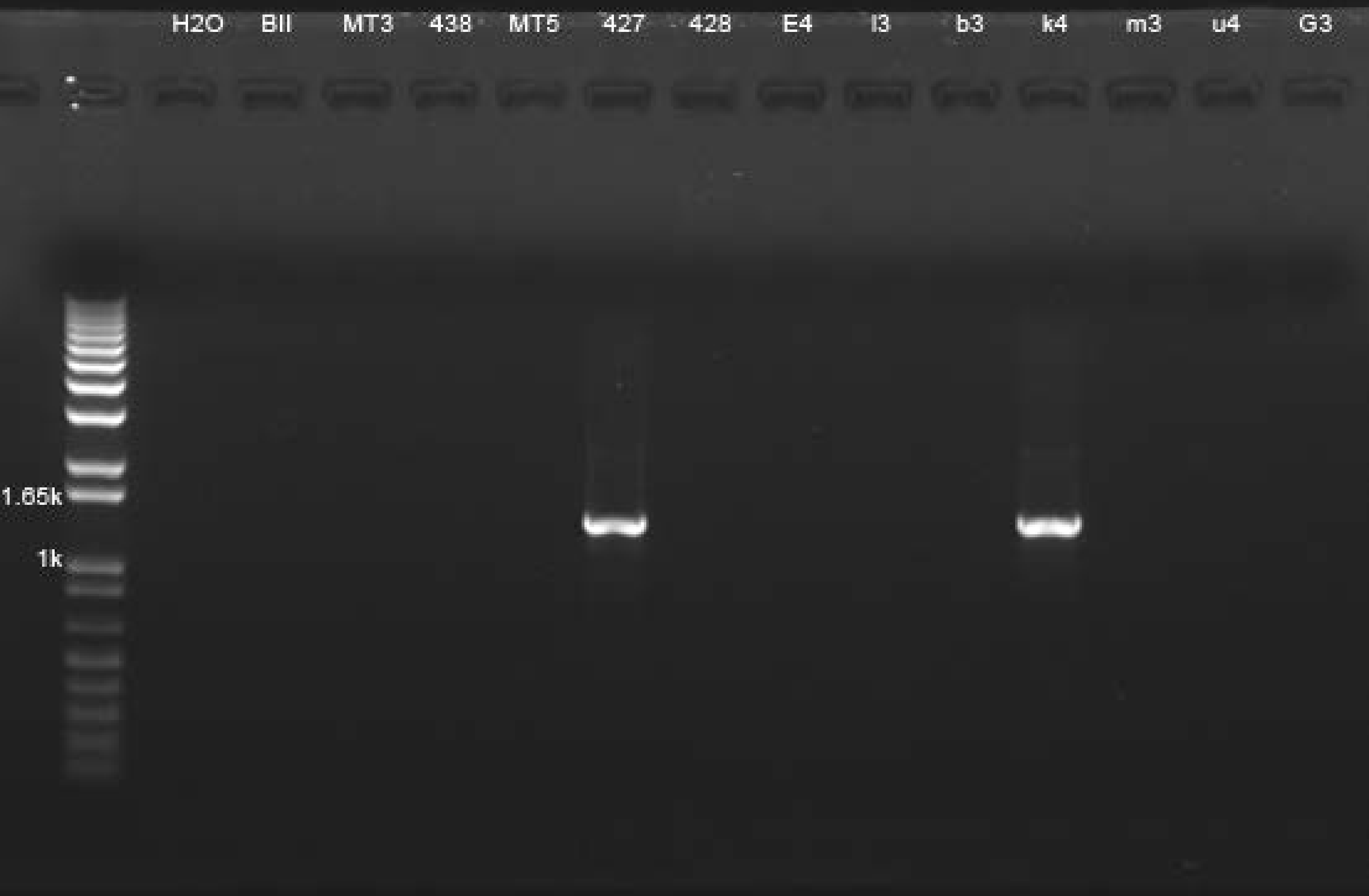

Fig2A PCR MTA6

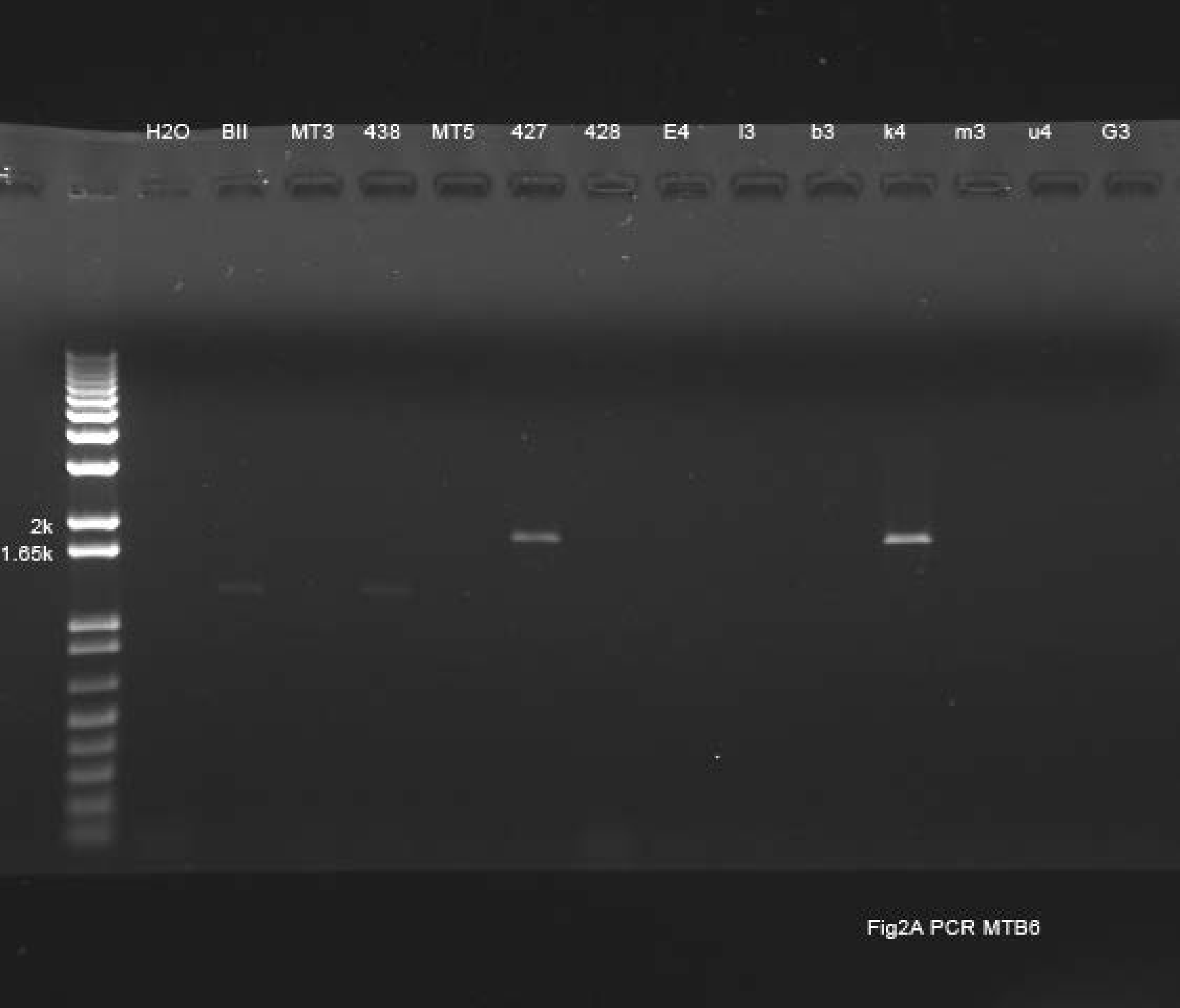

Fig2A PCR MTB6

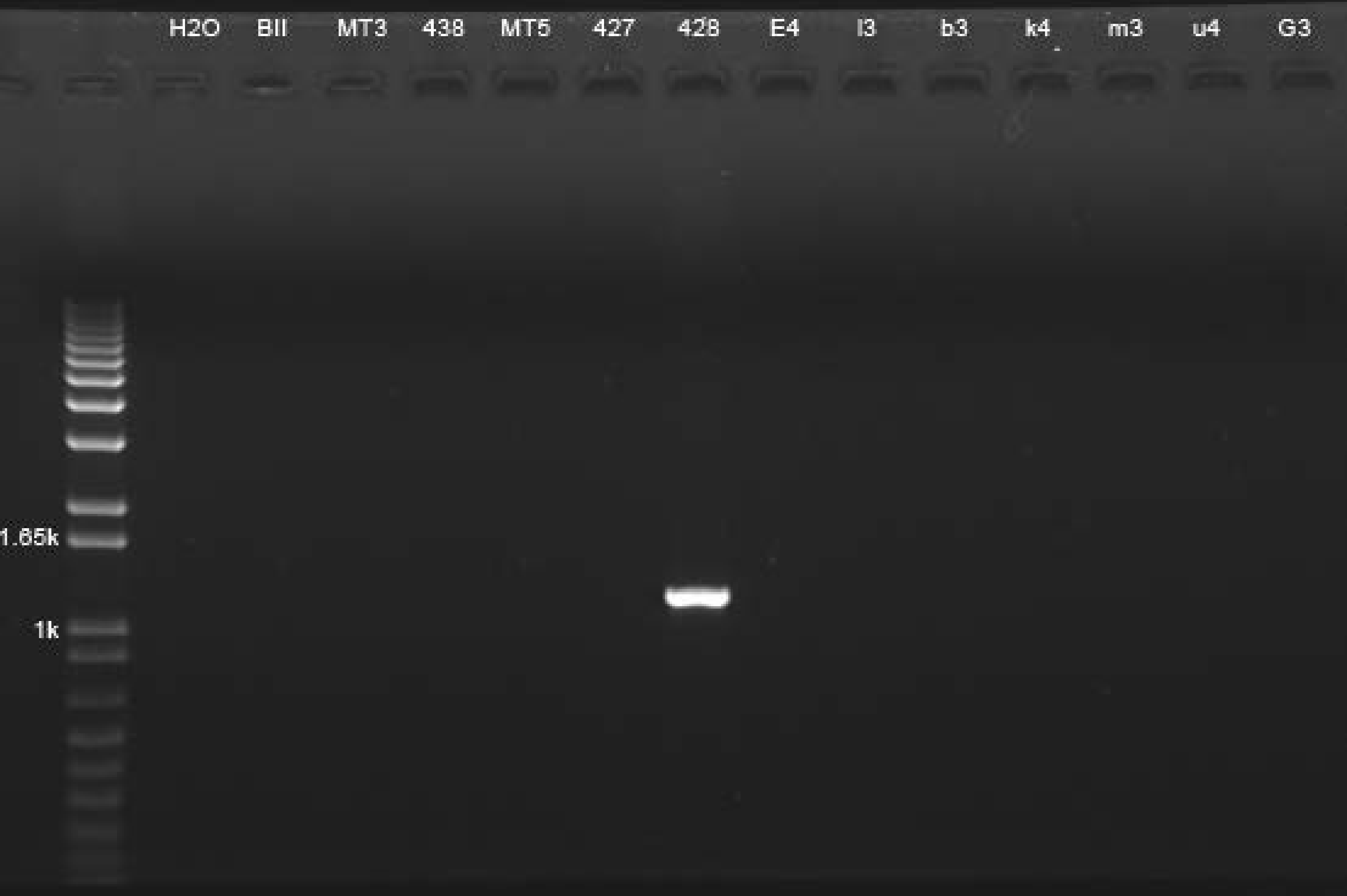

Fig2A PCR MTA7

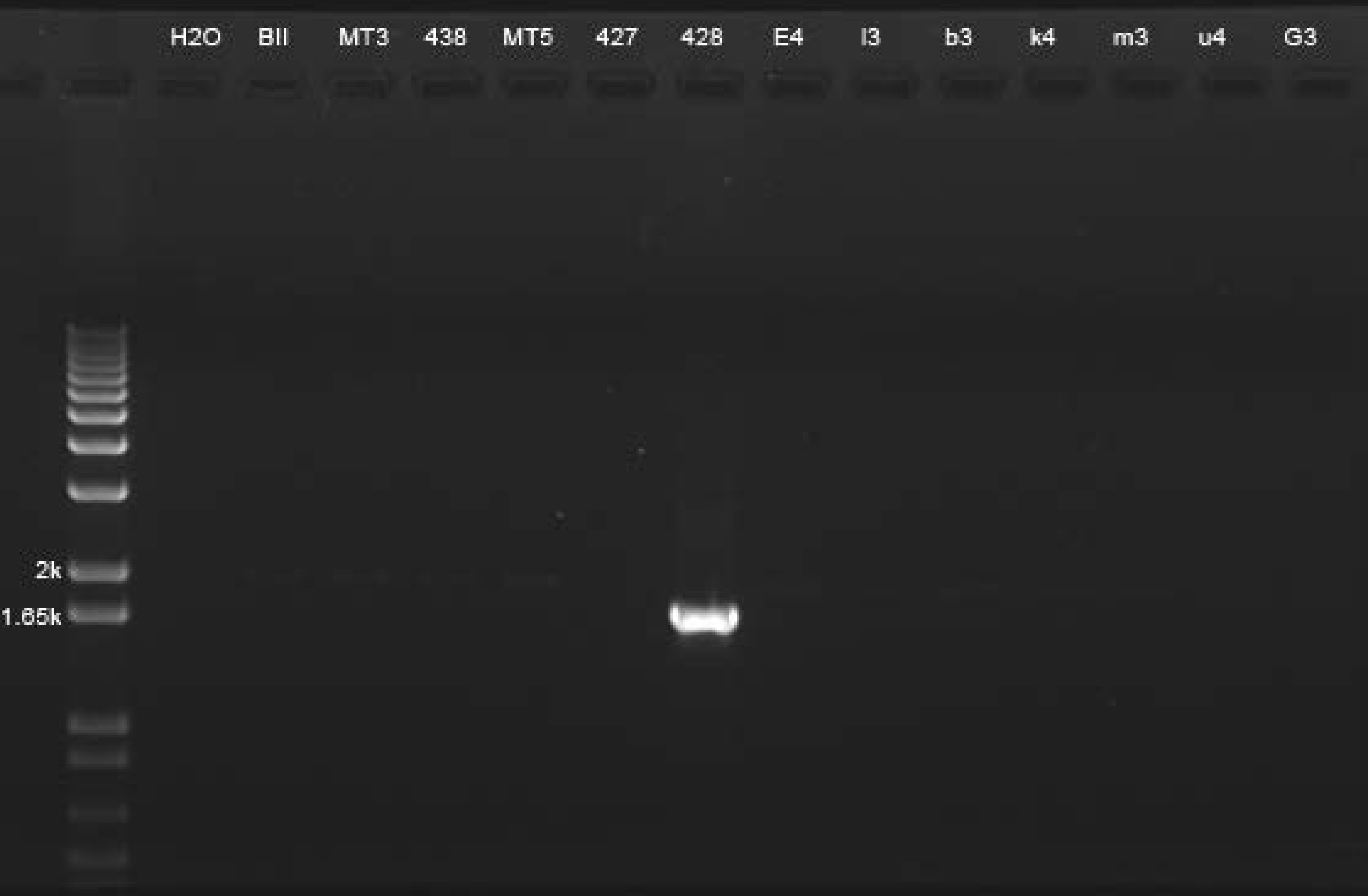

Fig2A PCR MTB7

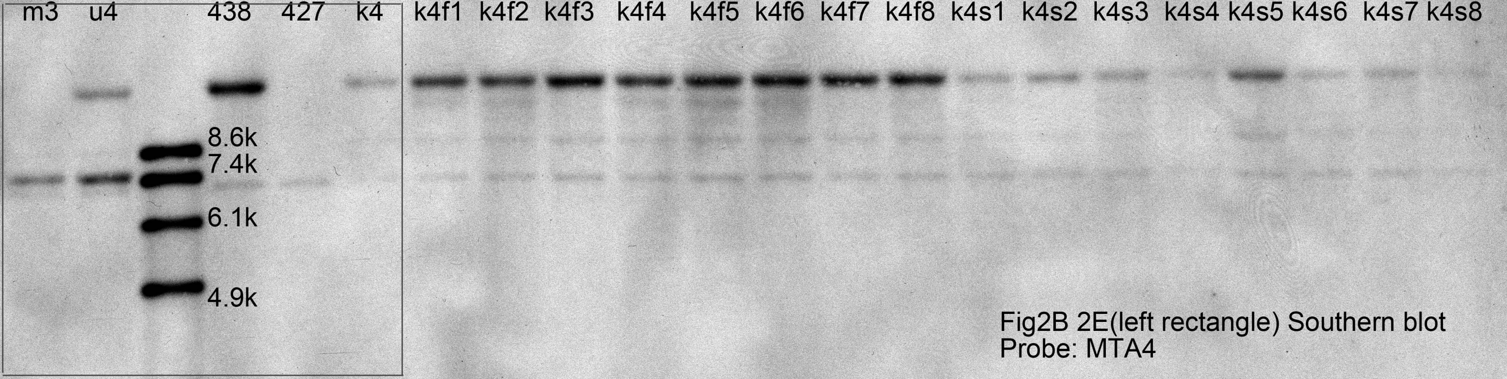

Fig2B 2E(left rectangle) Southern blot
Probe: MTA4

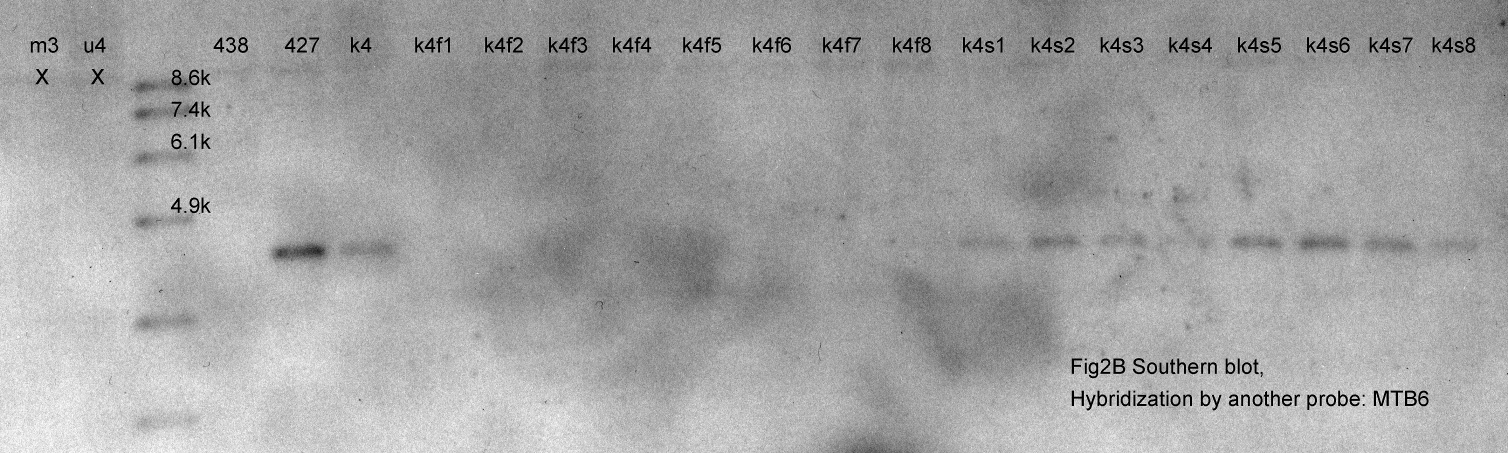

Fig2B Southern blot,
Hybridization by another probe: MTB6

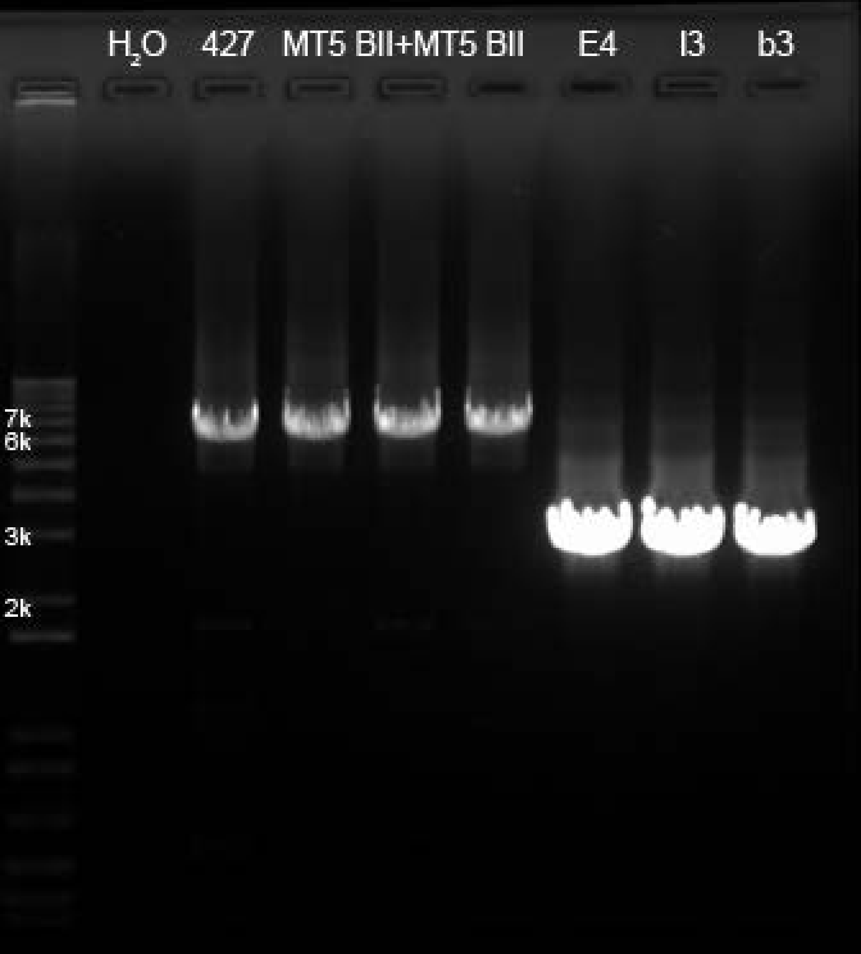
H2O
427
MT5 BII+MT5 BII
E4
I3
b3
7k
6k
3k
2k

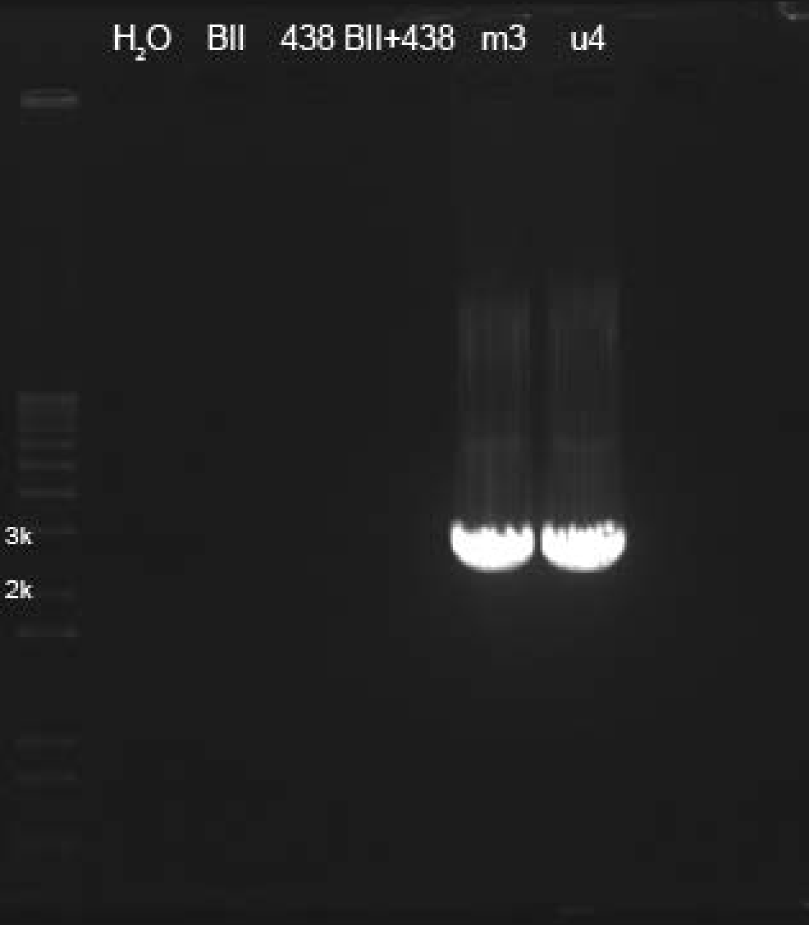

H_2O BII 438 BII+438 m3 u4
3k
2k

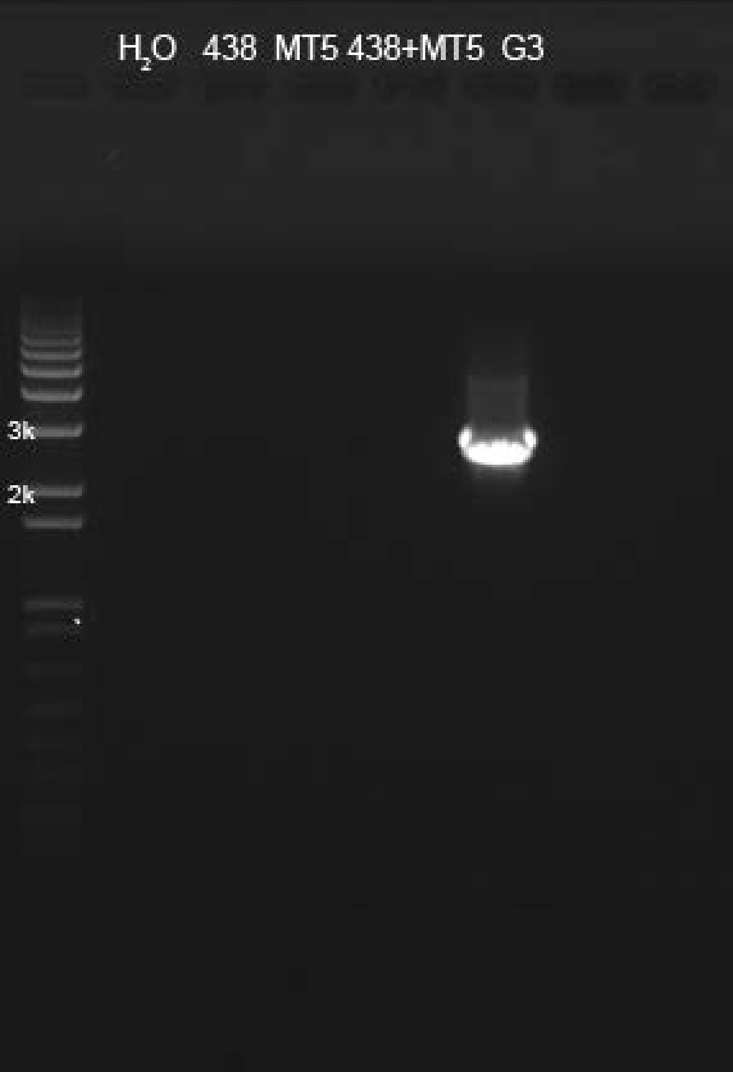

H2O 438 MT5 438+MT5 G3
3k
2k

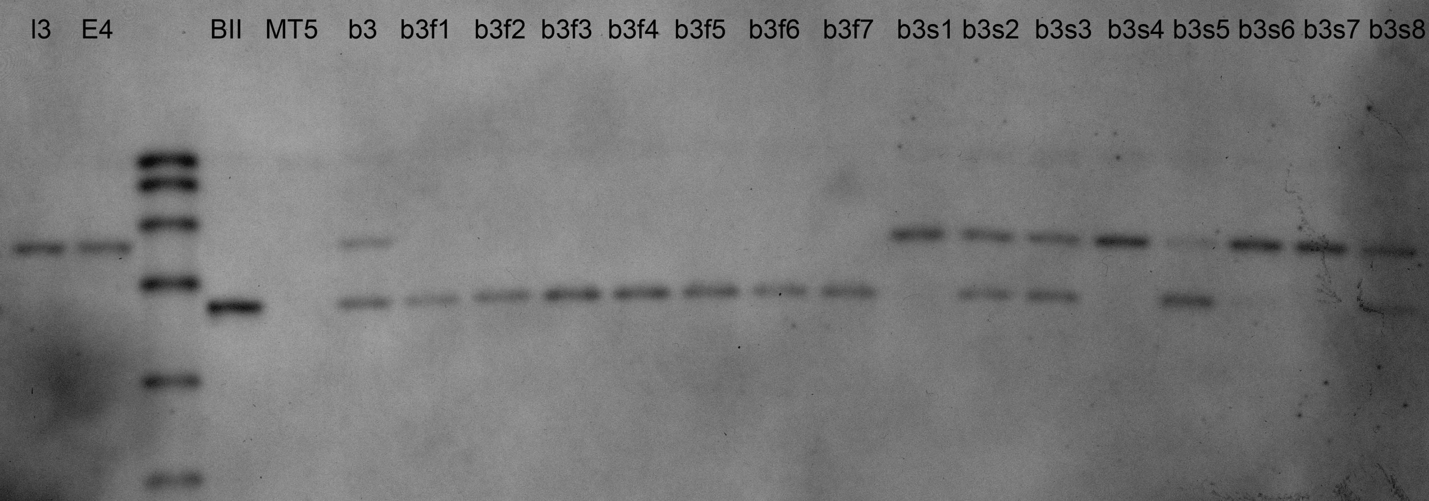
I3 E4 BII MT5 b3 b3f1 b3f2 b3f3 b3f4 b3f5 b3f6 b3f7 b3s1 b3s2 b3s3 b3s4 b3s5 b3s6 b3s7 b3s8

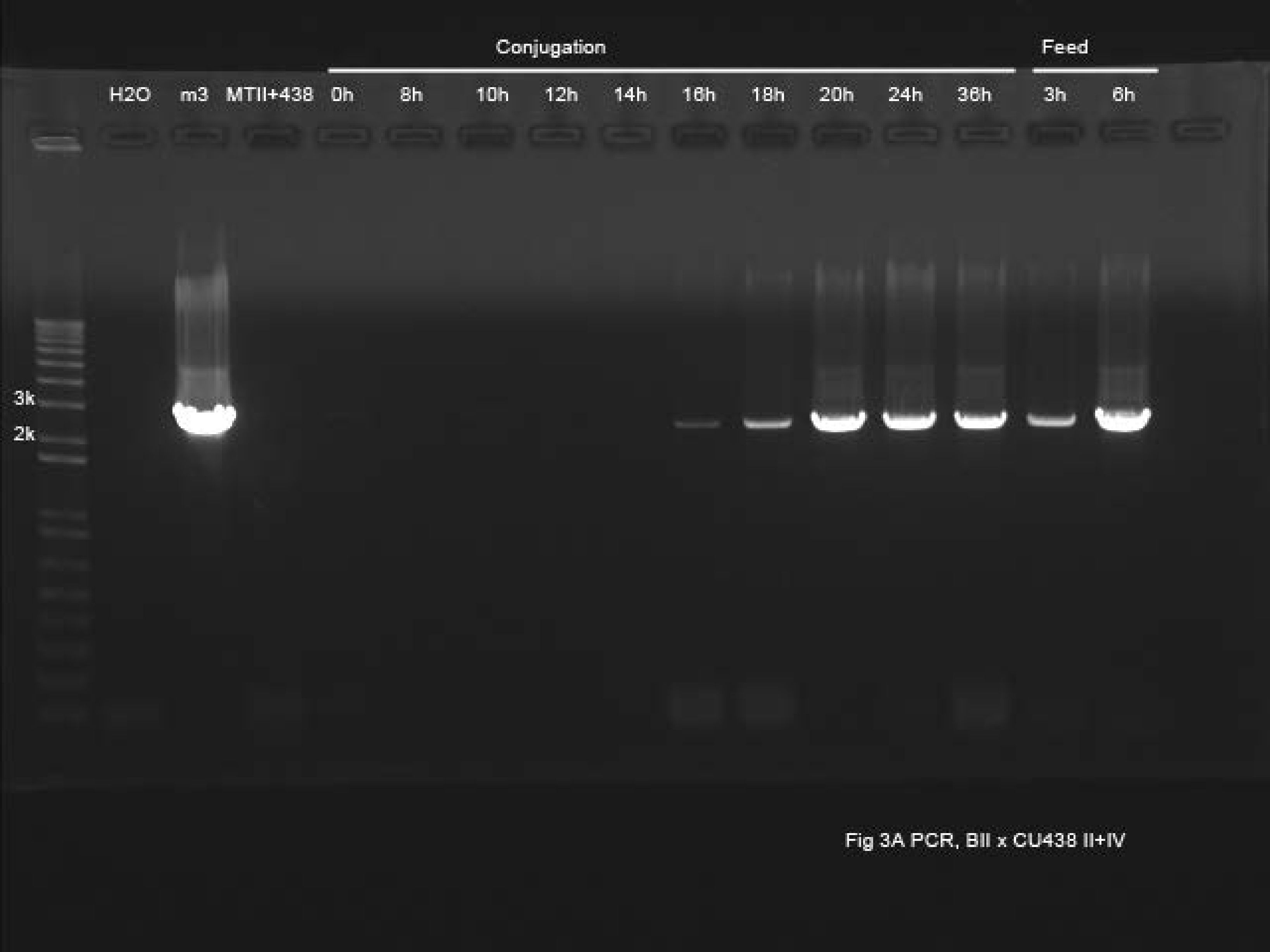
Conjugation
Feed
H2O
m3
MTII+438
0h
8h
10h
12h
14h
16h
18h
20h
24h
36h
3h
6h
3k
2k
Fig 3A PCR, BII x CU438 II+IV

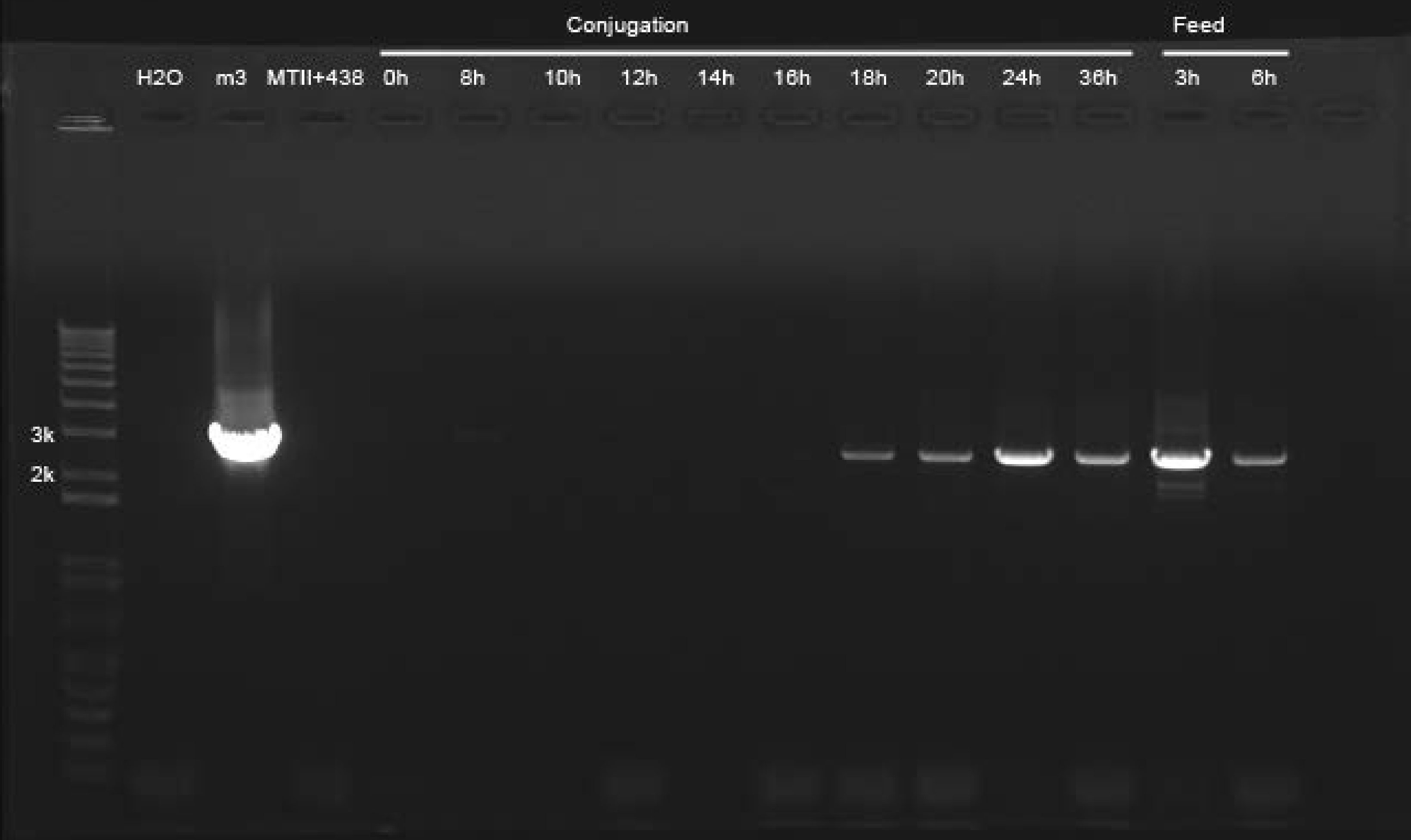

Fig 3A PCR, TKU80 germline knockout II+IV

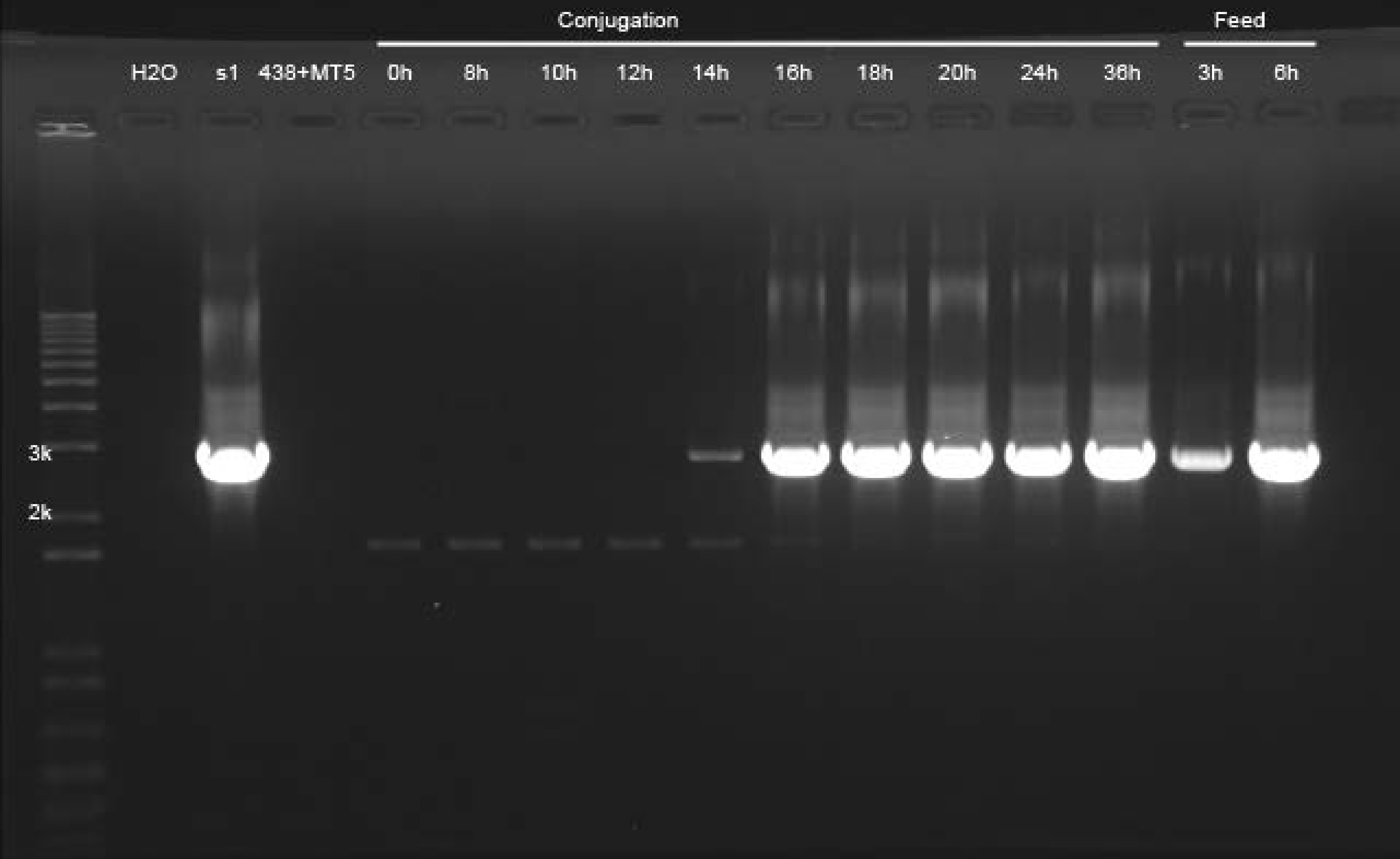

Fig 3A PCR, BII x CU438 IV+V

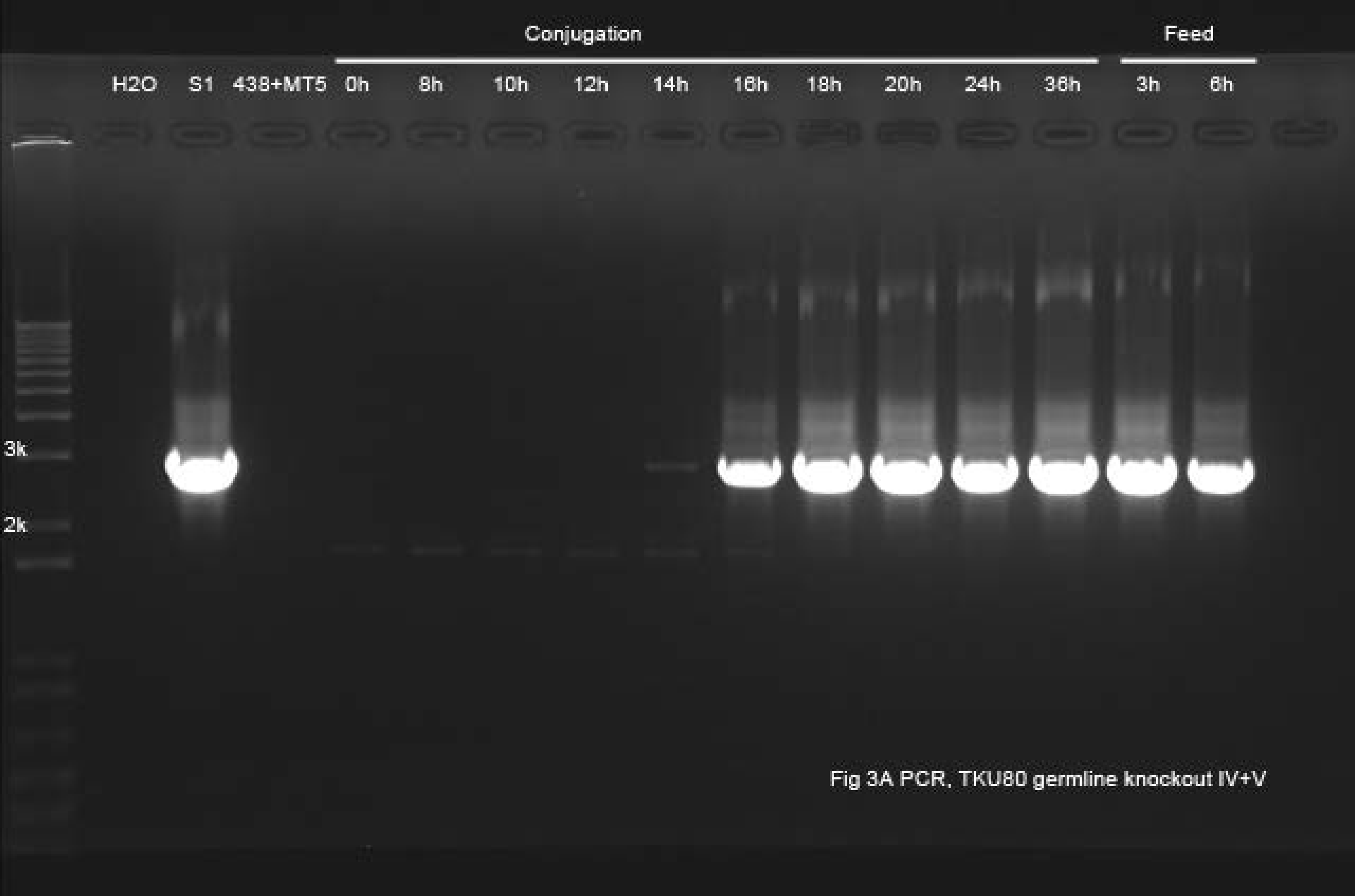
Conjugation
Feed
H2O
S1
438+MT5
0h
8h
10h
12h
14h
16h
18h
20h
24h
36h
3h
6h
3k
2k
Fig 3A PCR, TKU80 germline knockout IV+V

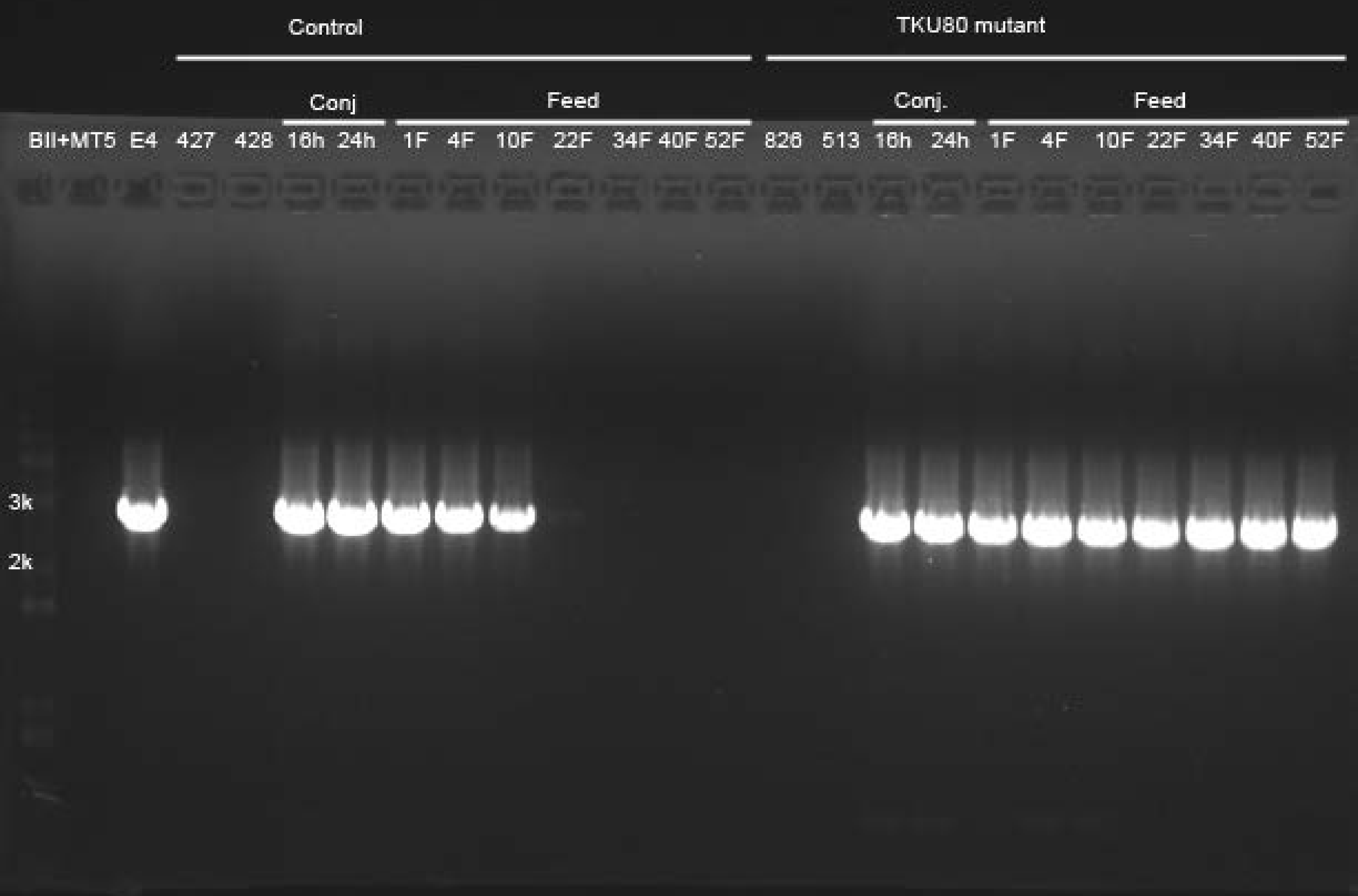

Fig 3B PCR, II+V, left

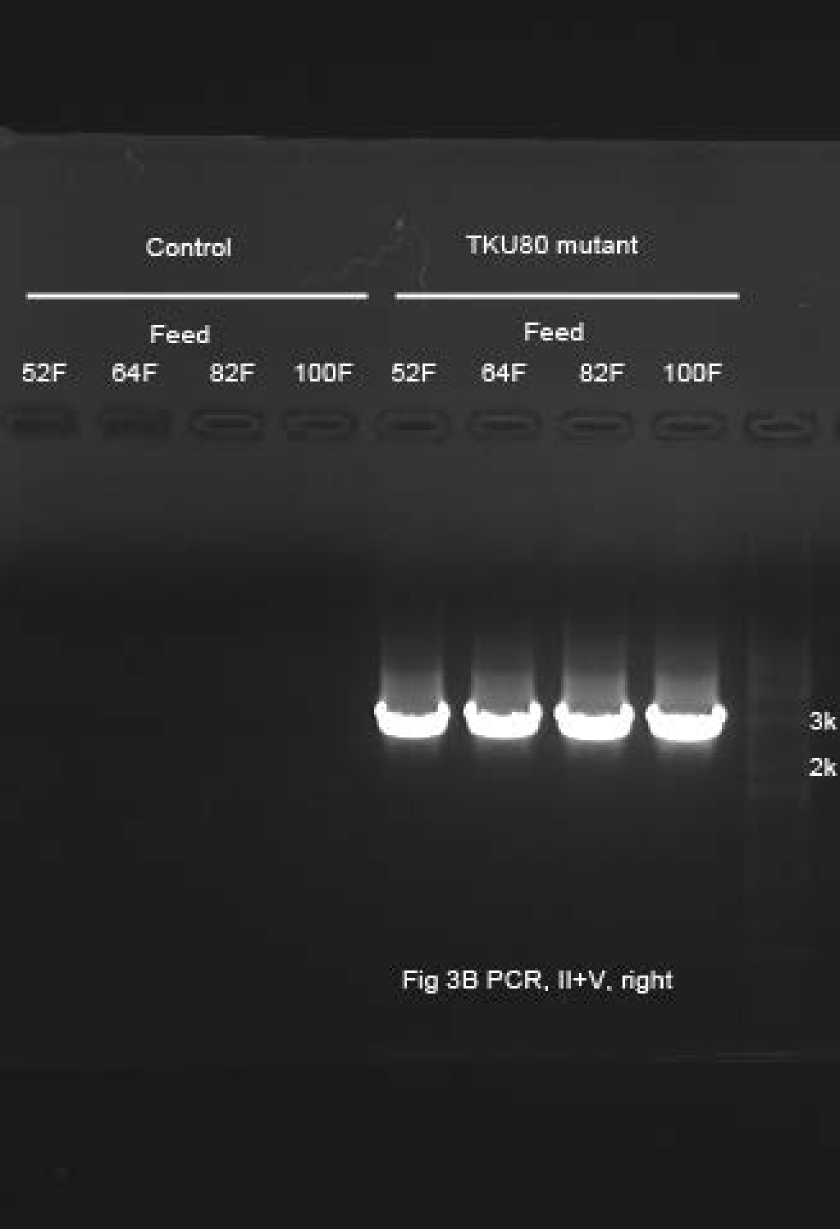
Control
TKU80 mutant
Feed
Feed
52F
64F
82F
100F
52F
64F
82F
100F
3k
2k
Fig 3B PCR, II+V, right

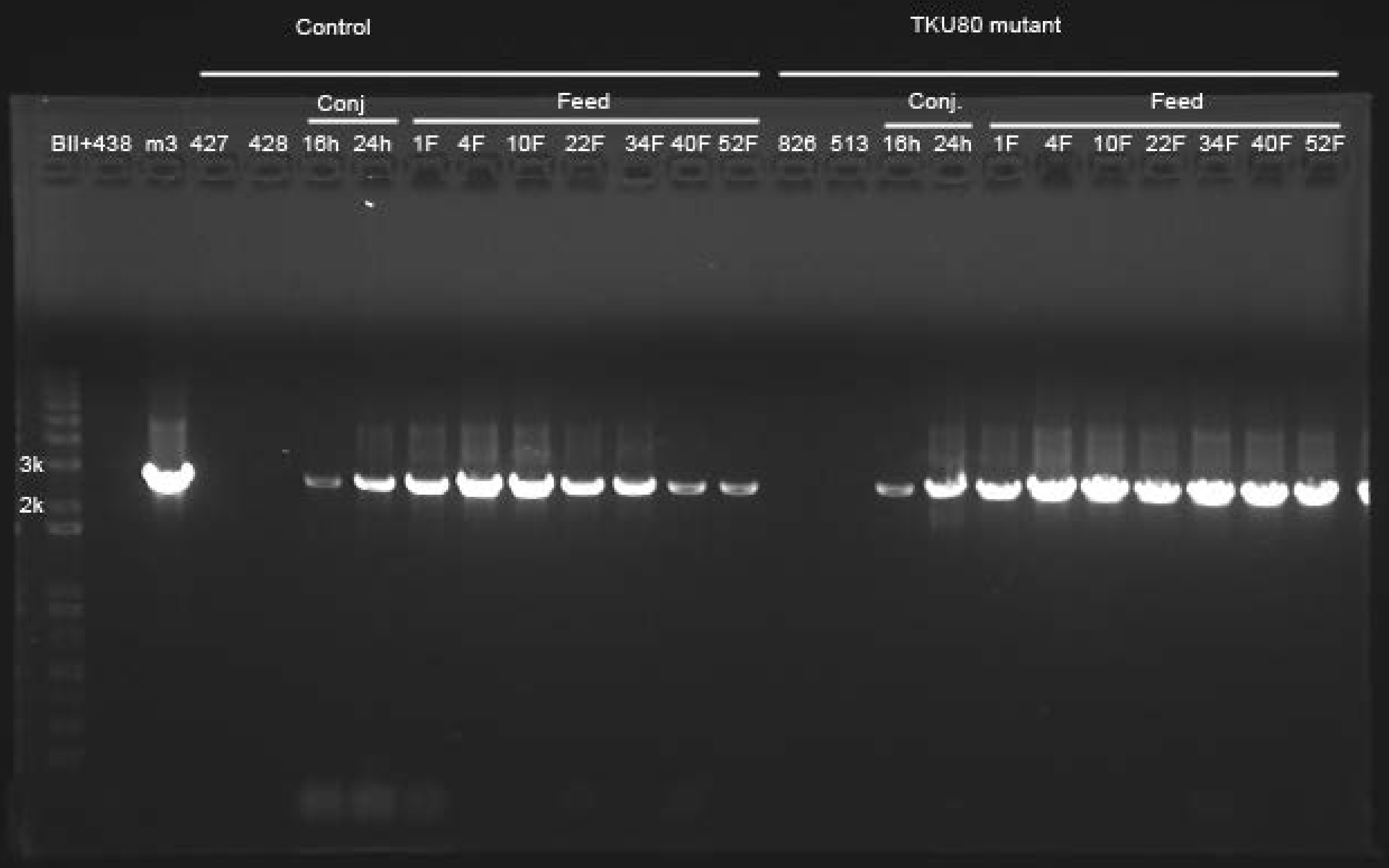

Fig 3B PCR, II+IV, left

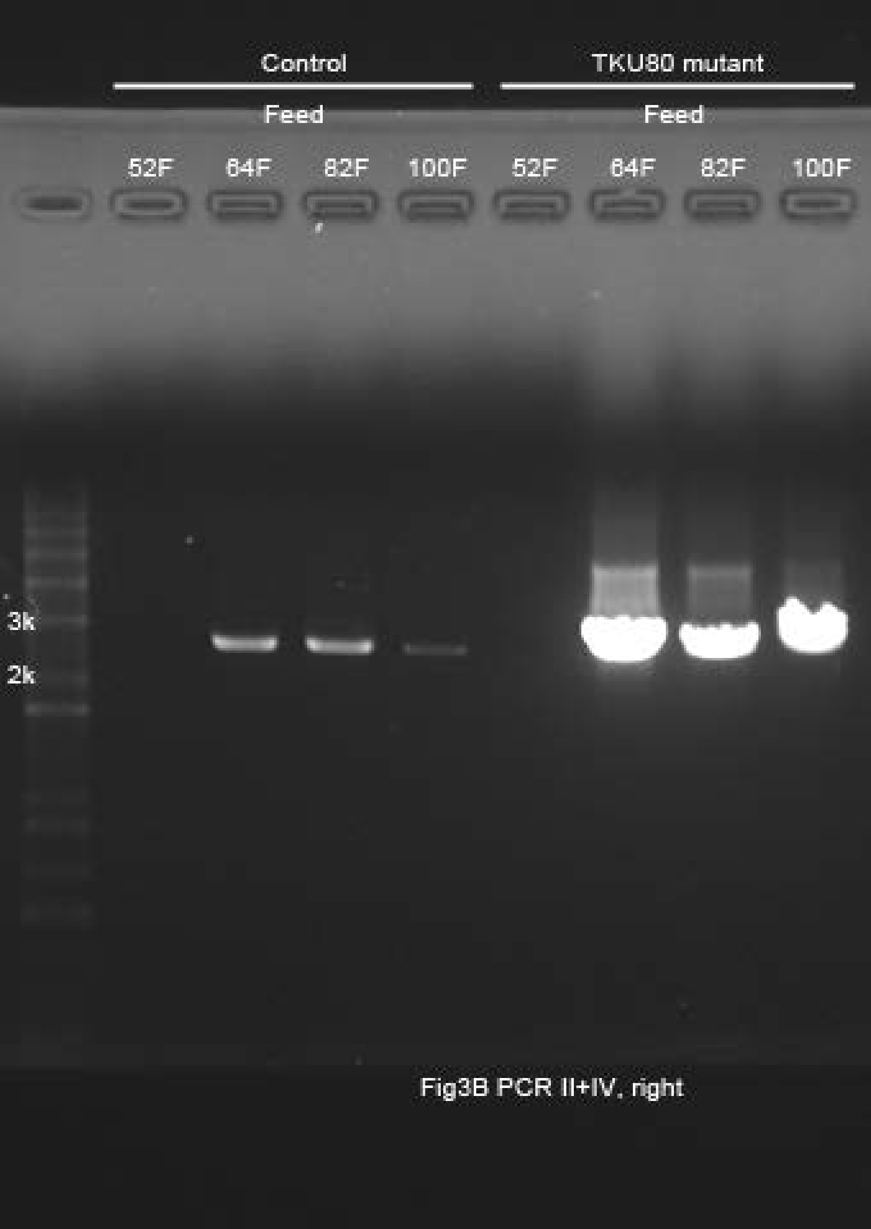
Control
TKU80 mutant
Feed
Feed
52F
64F
82F
100F
52F
64F
82F
100F
3k
2k
Fig3B PCR II+IV, right

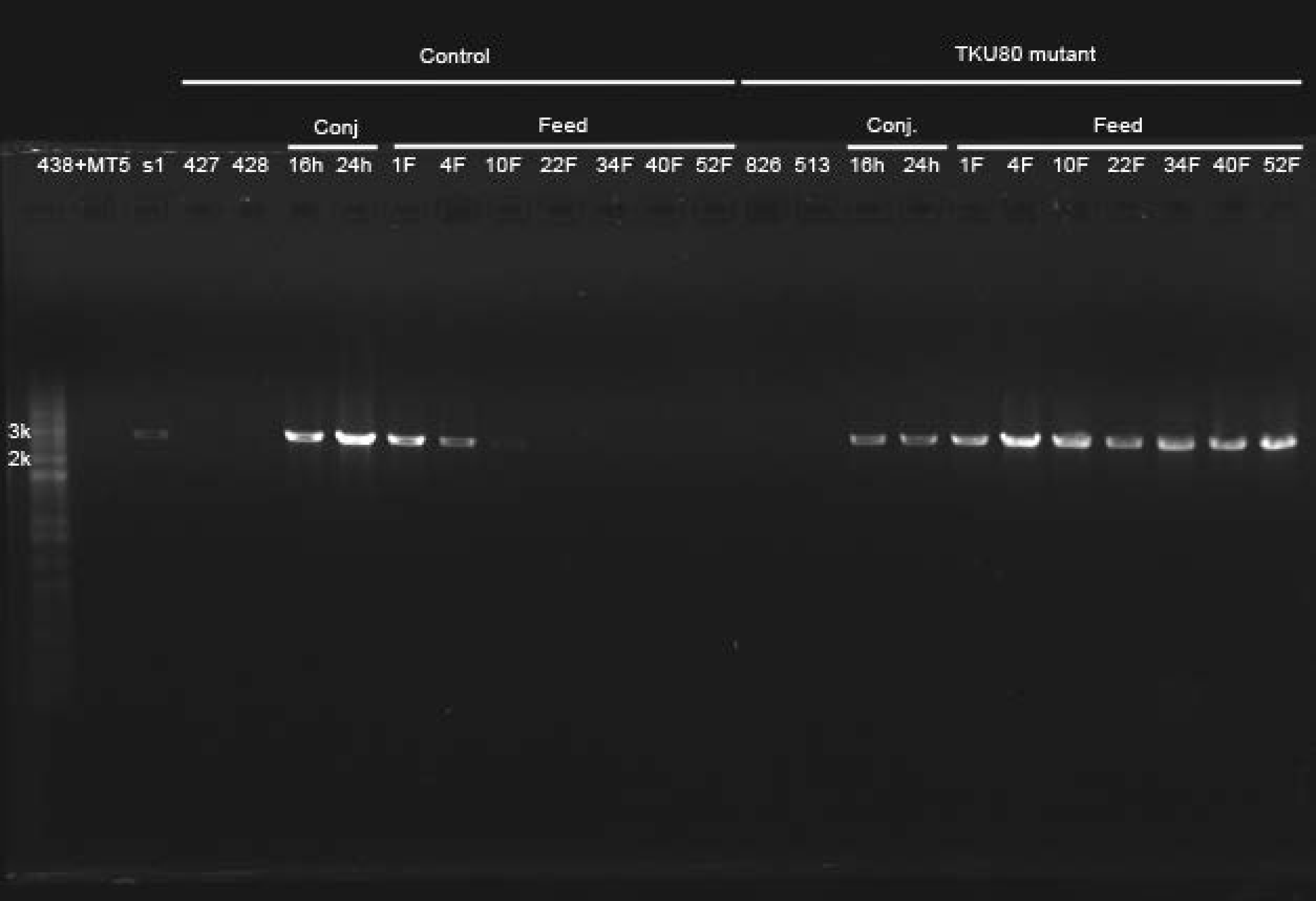

Fig 3B PCR, IV+V, left

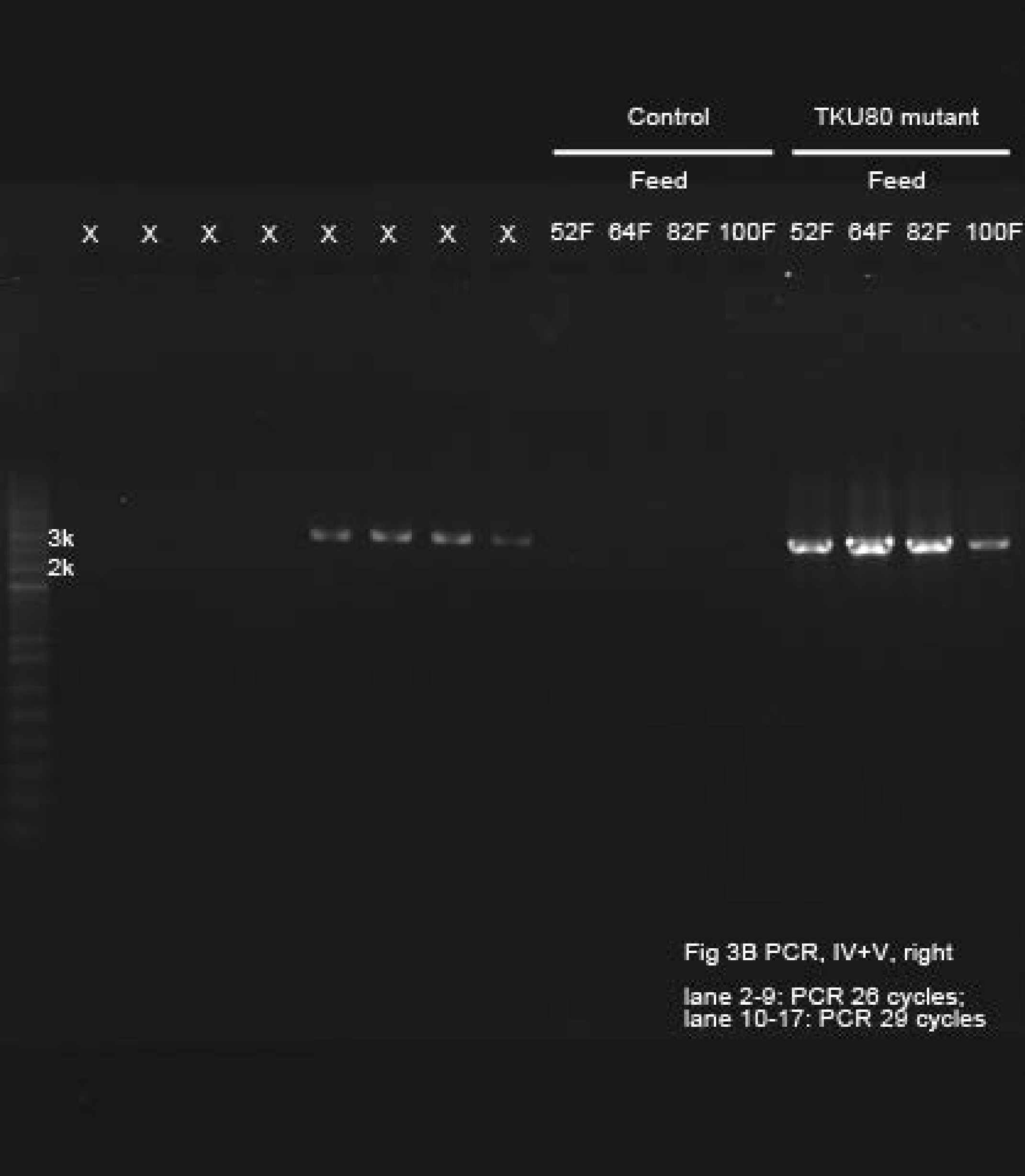
Control
TKU80 mutant
Feed
Feed
X X X X X X X X 52F 64F 82F 100F 52F 64F 82F 100F
3k
2k
Fig 3B PCR, IV+V, right
lane 2-9: PCR 26 cycles;
lane 10-17: PCR 29 cycles

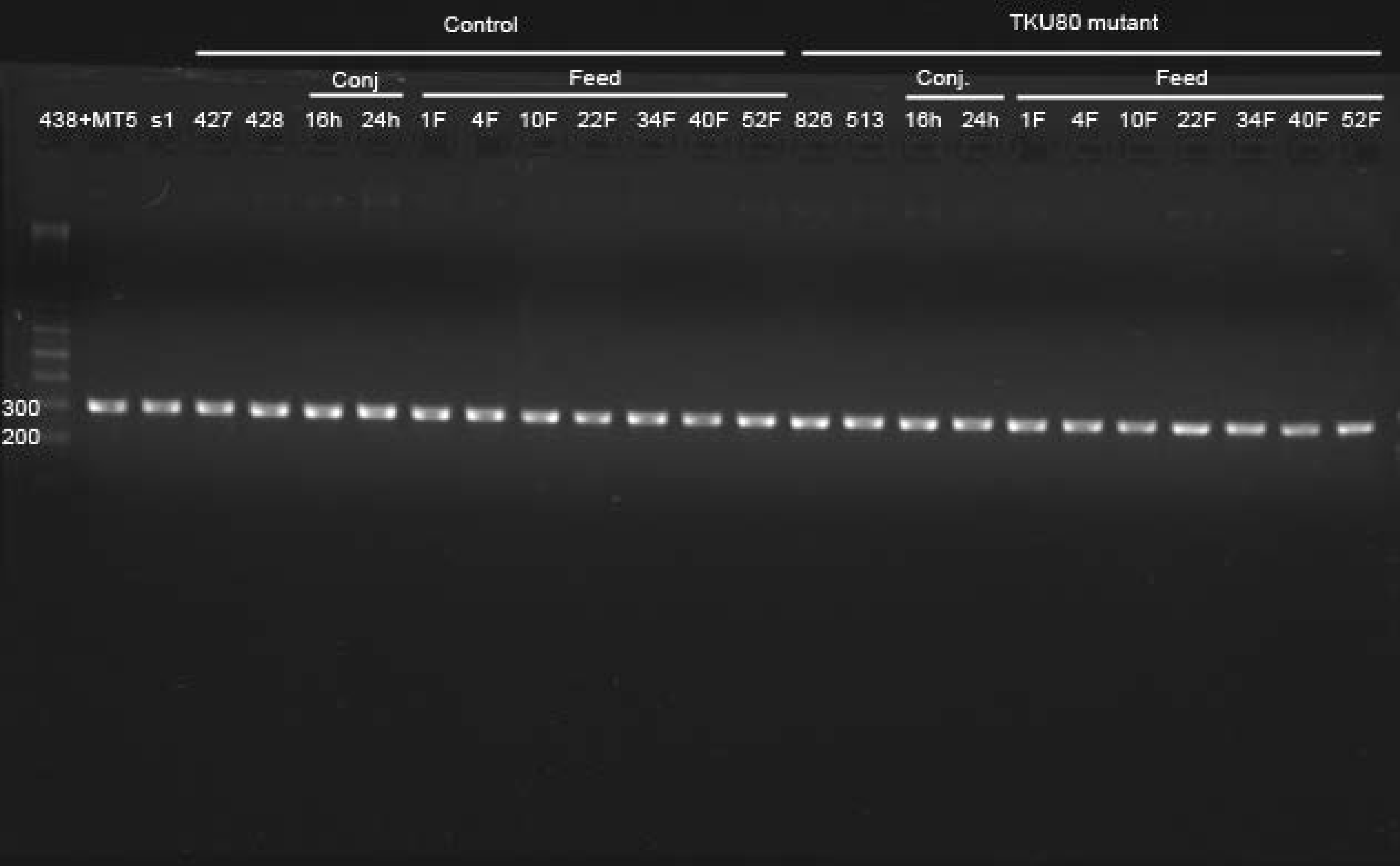

Fig 3B PCR, atub, left

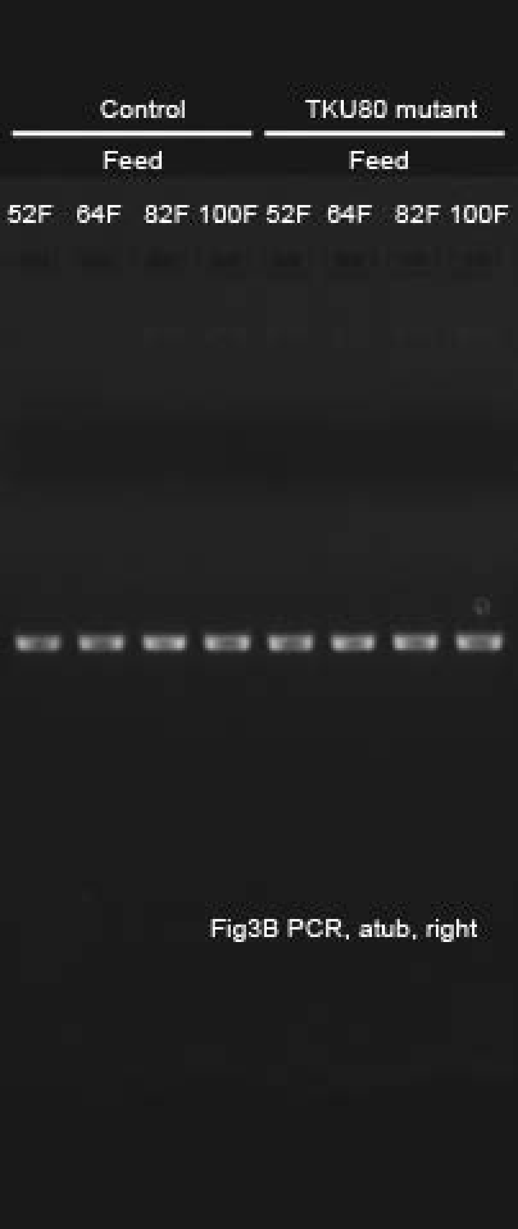
Control
TKU80 mutant
Feed
Feed
52F 64F 82F 100F 52F 64F 82F 100F
Fig3B PCR, atub, right

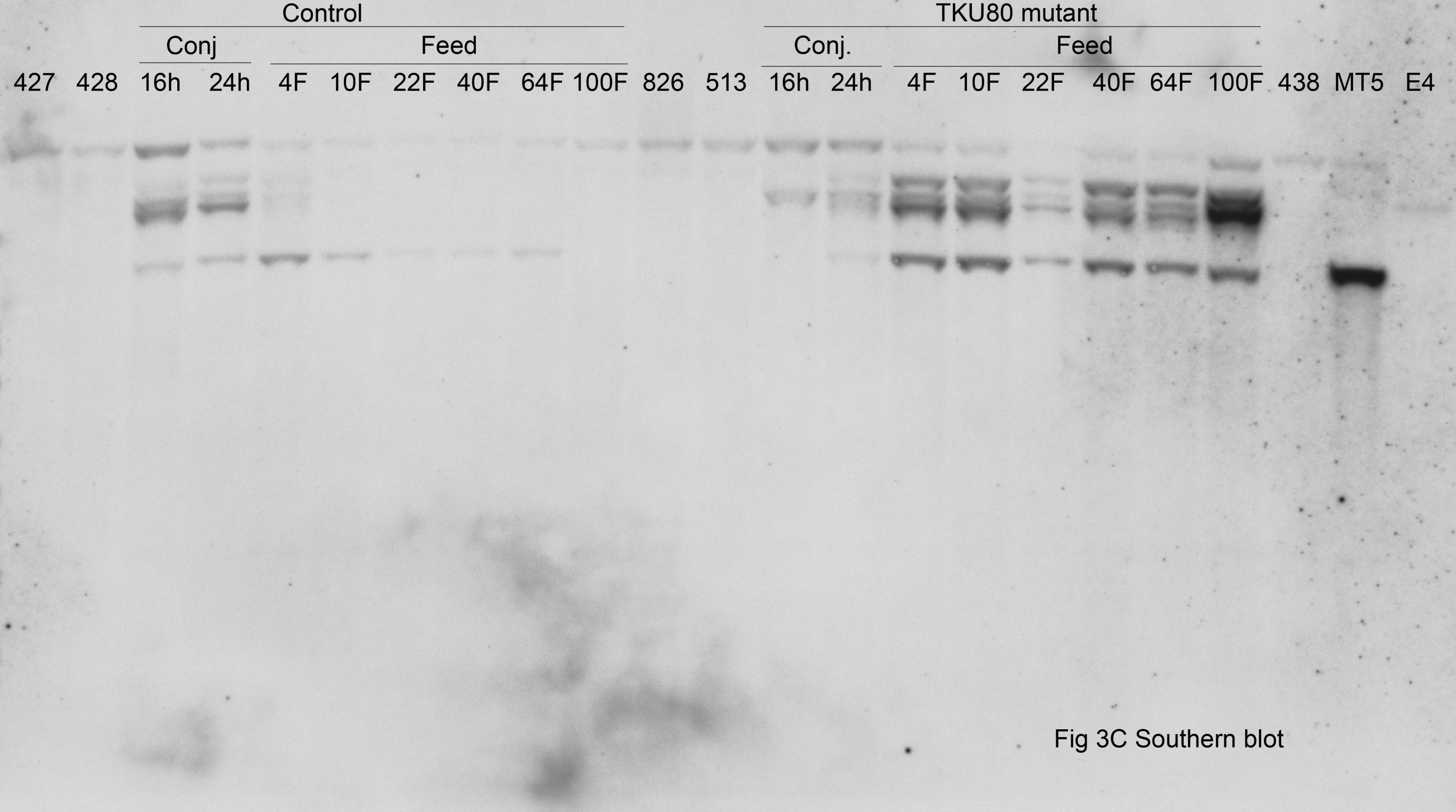

Fig 3C Southern blot

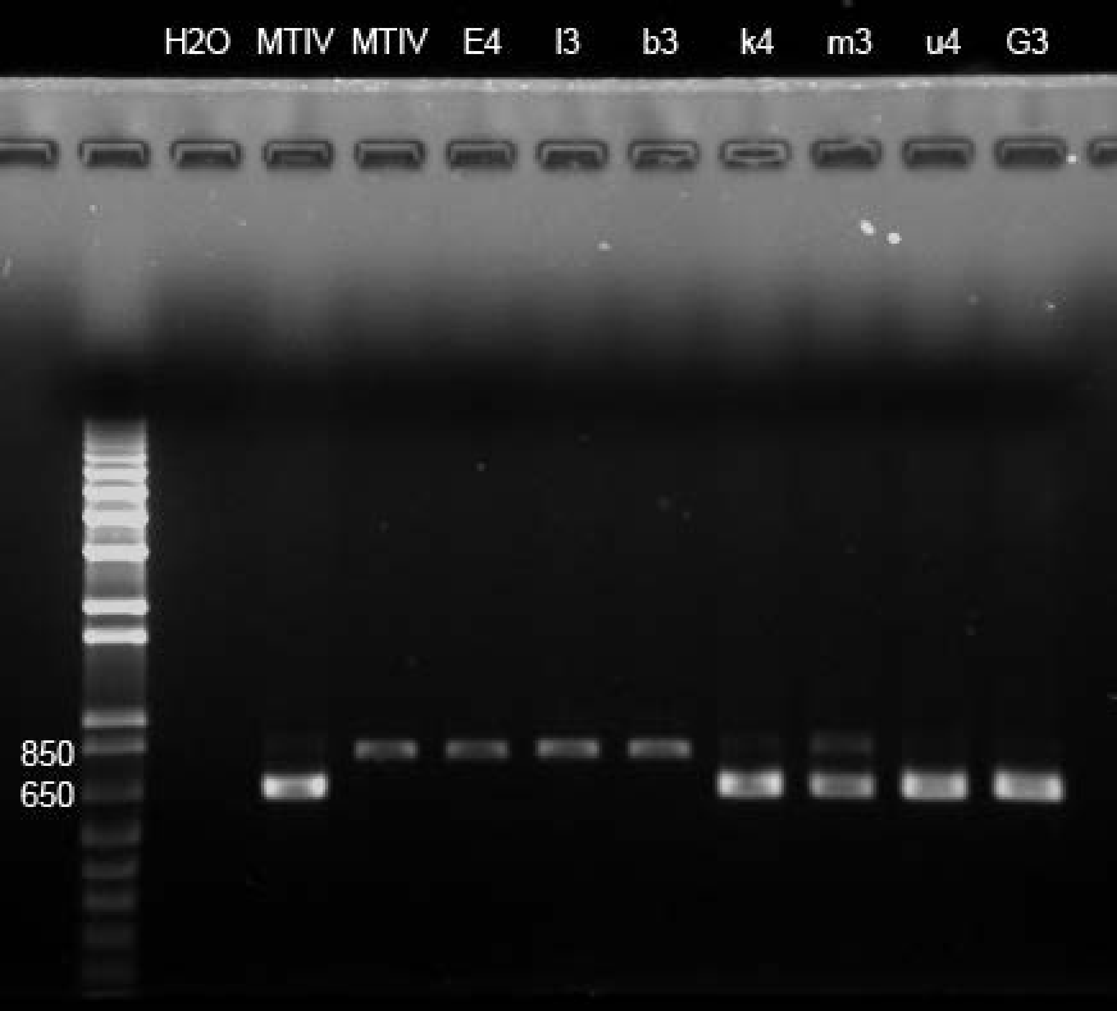

Supp fig S6 RT-PCR MTA4 RT+

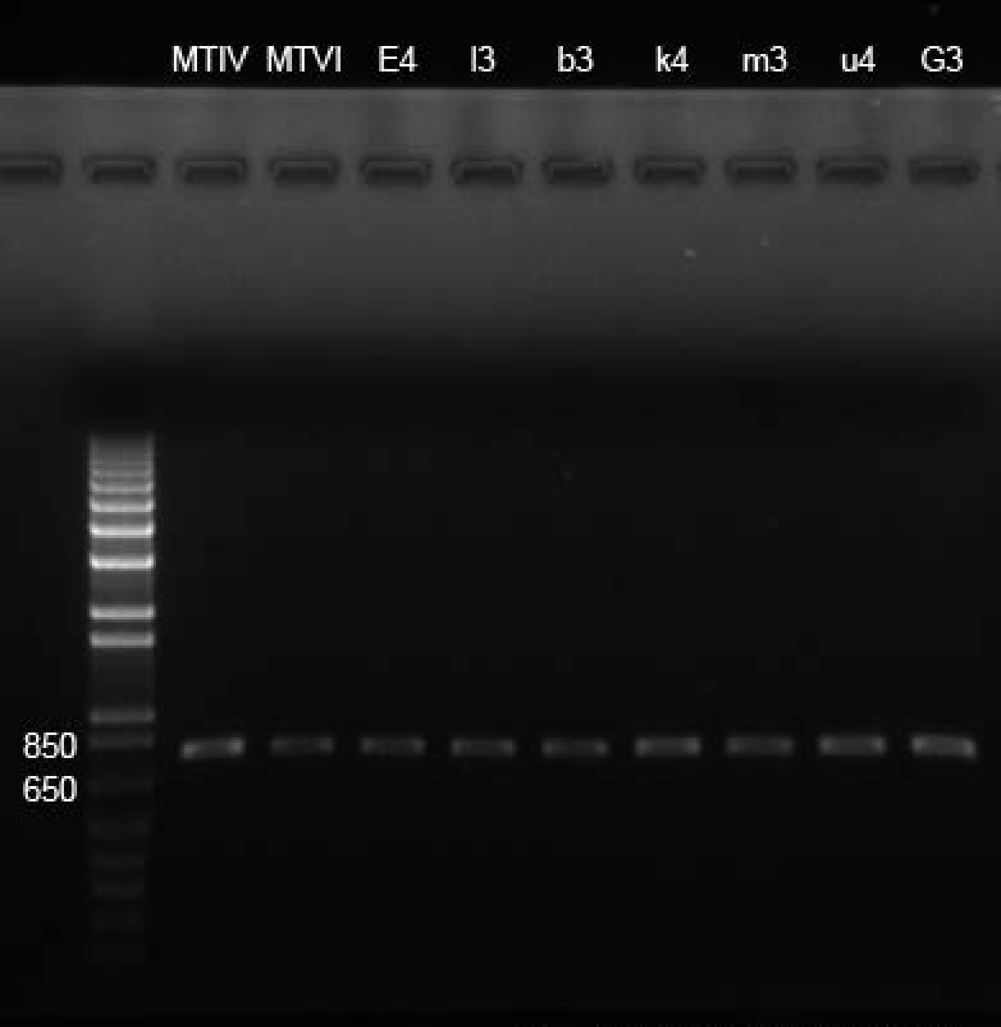

Supp fig S6 RT-PCR MTA4 RT-

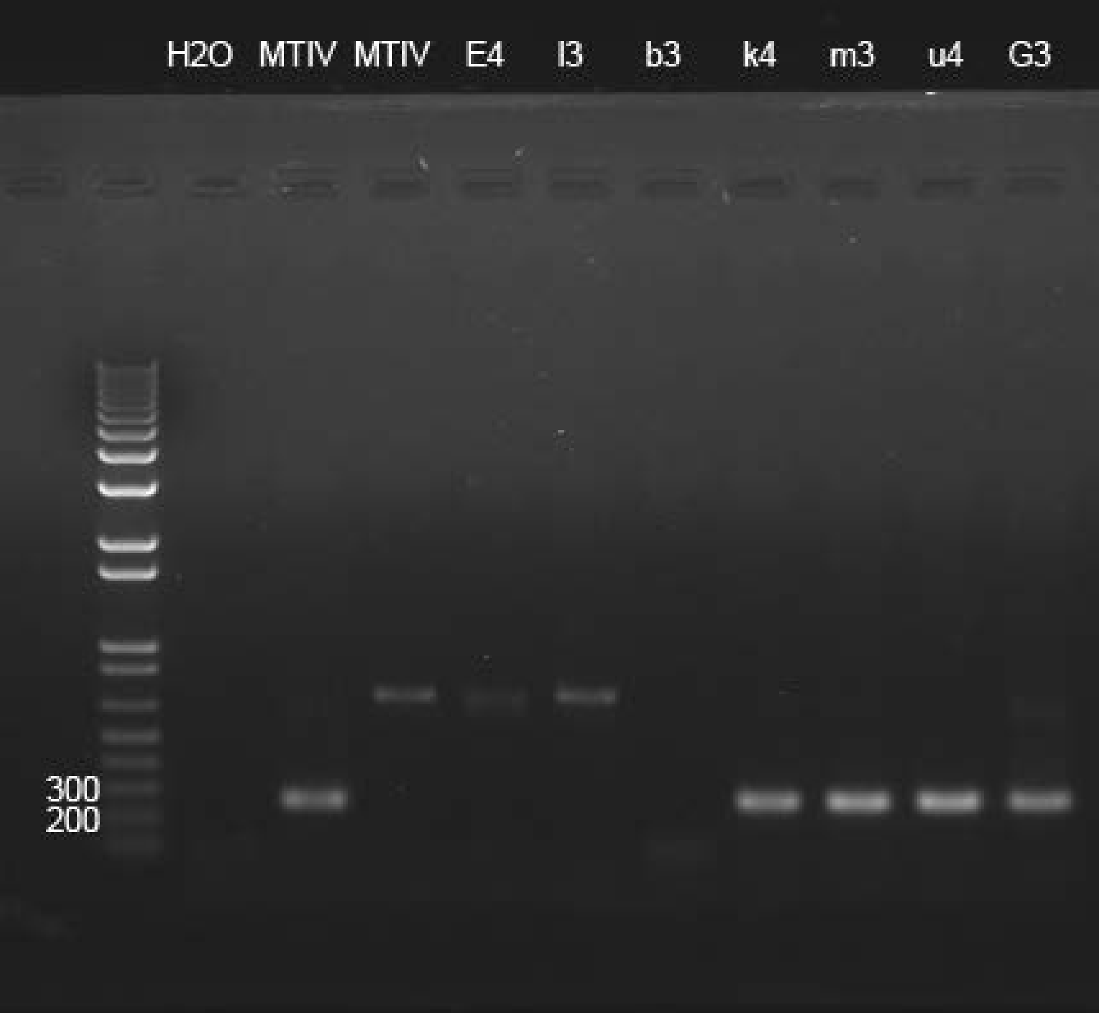

Supp fig S6 RT-PCR MTB4 RT+

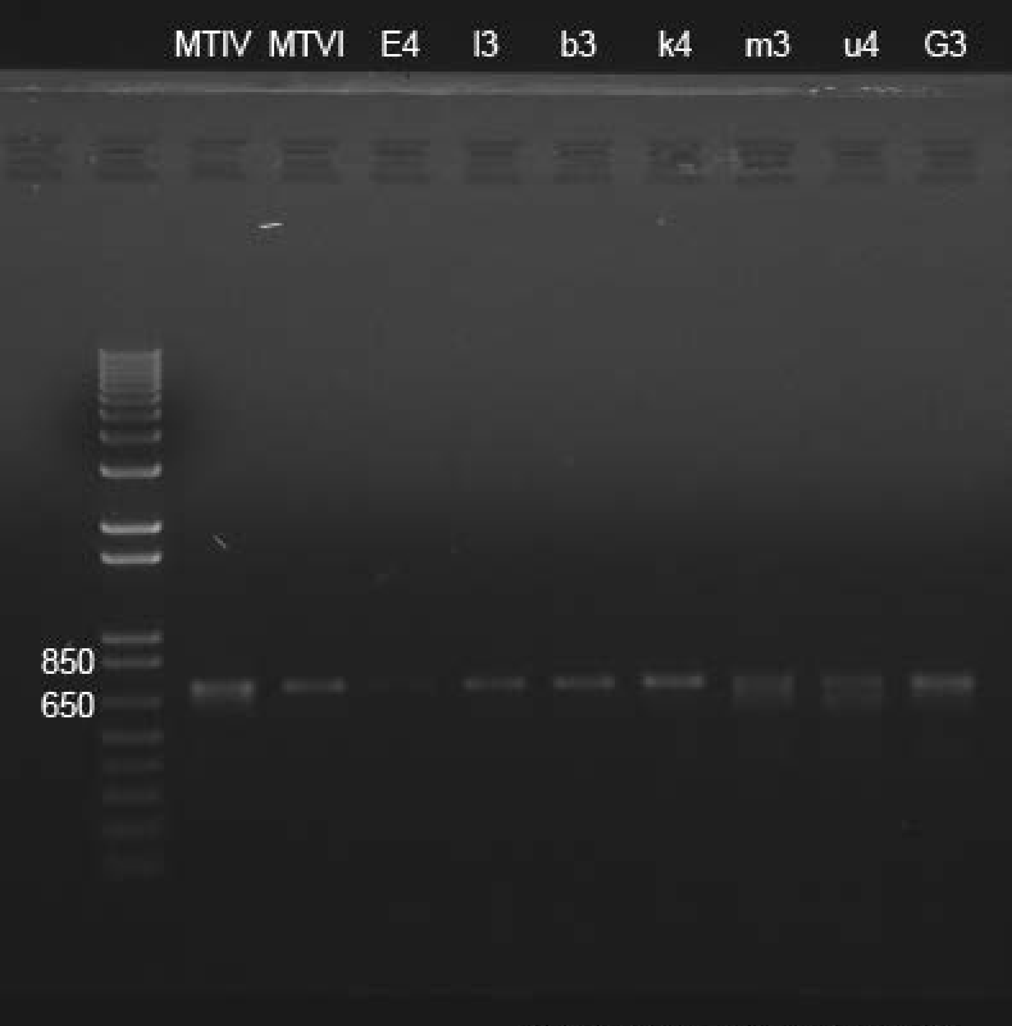

Supp fig S6 RT-PCR MTB4 RT-

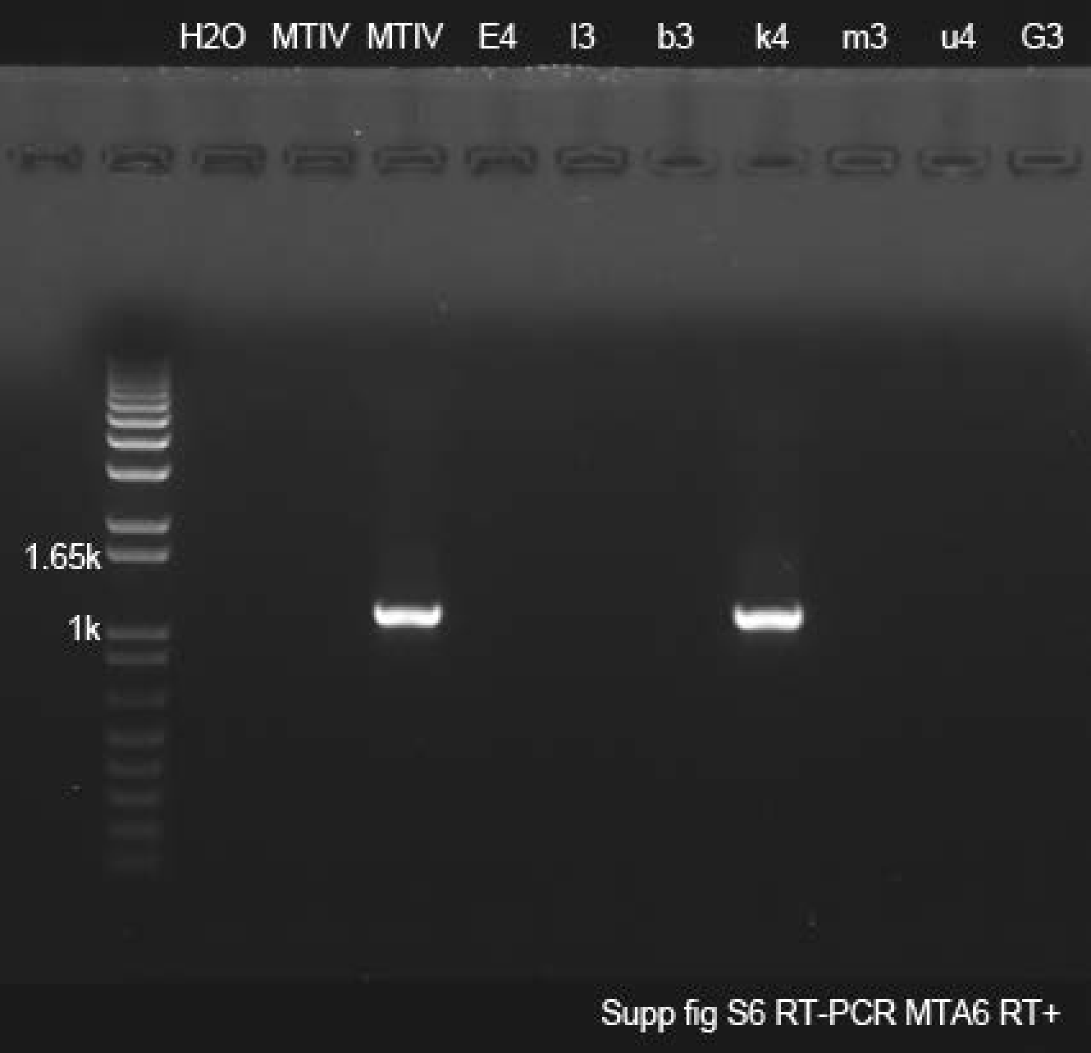
H2O
MTIV
MTIV
E4
I3
b3
k4
m3
u4
G3
1.65k
1k
Supp fig S6 RT-PCR MTA6 RT+

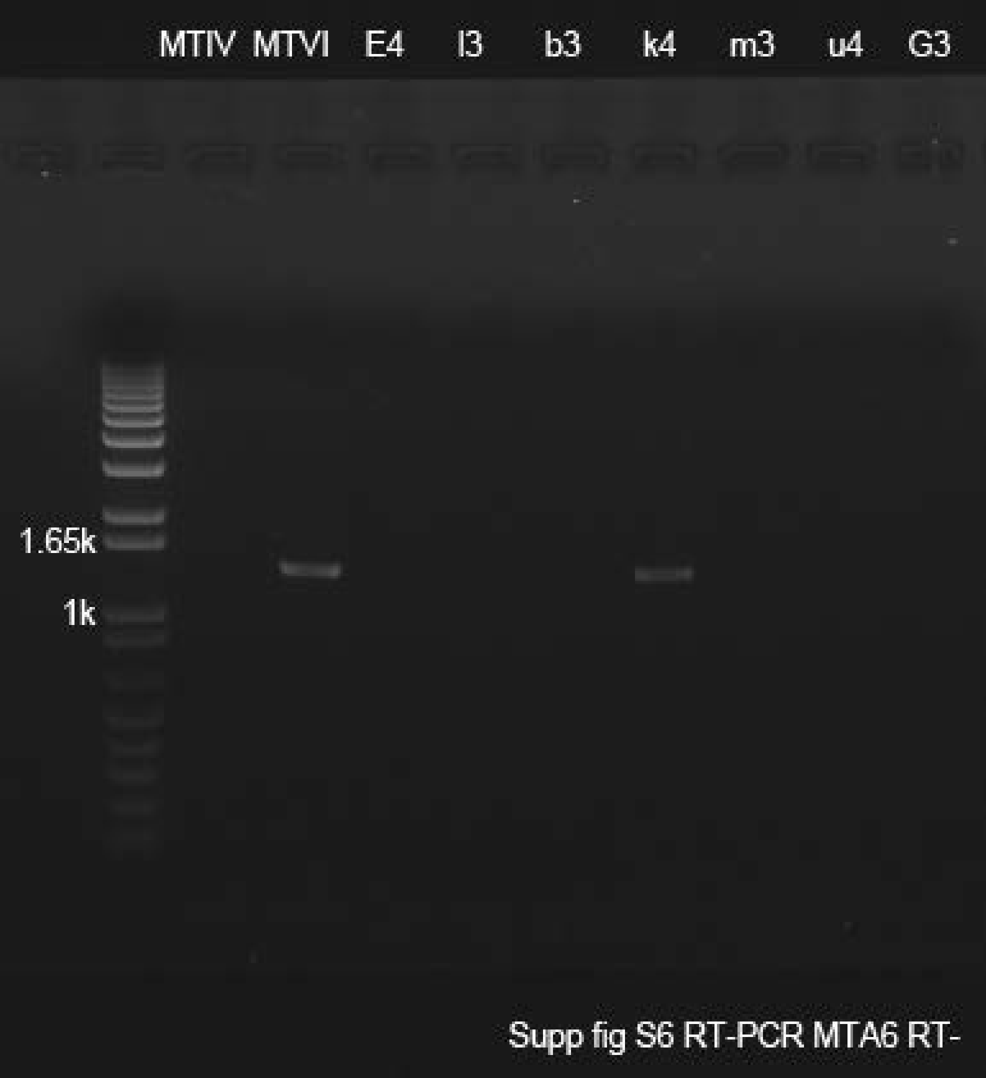
MTIV
MTVI
E4
I3
b3
k4
m3
u4
G3
1.65k
1k
Supp fig S6 RT-PCR MTA6 RT-

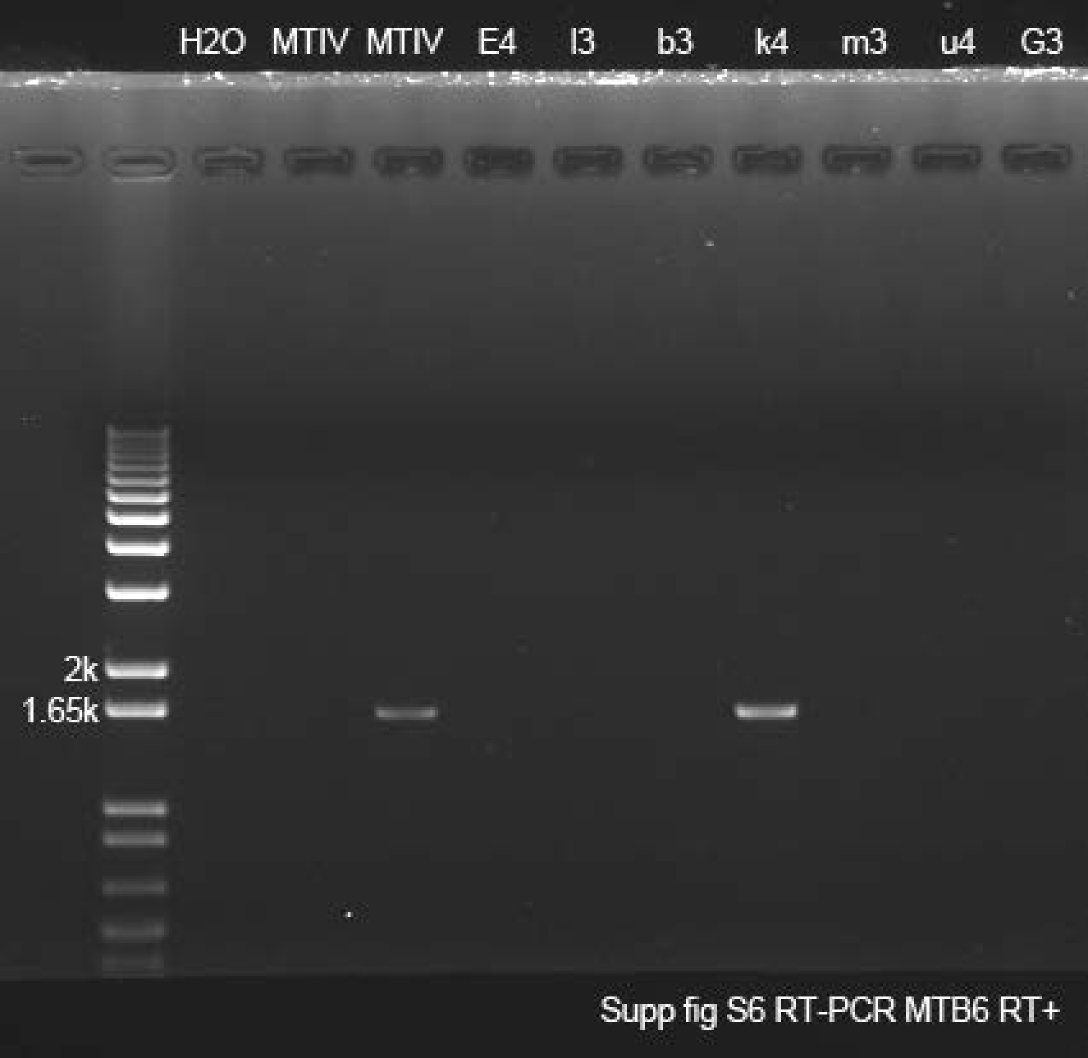

H2O
MTIV
MTIV
E4
I3
b3
k4
m3
u4
G3
2k
1.65k
Supp fig S6 RT-PCR MTB6 RT+

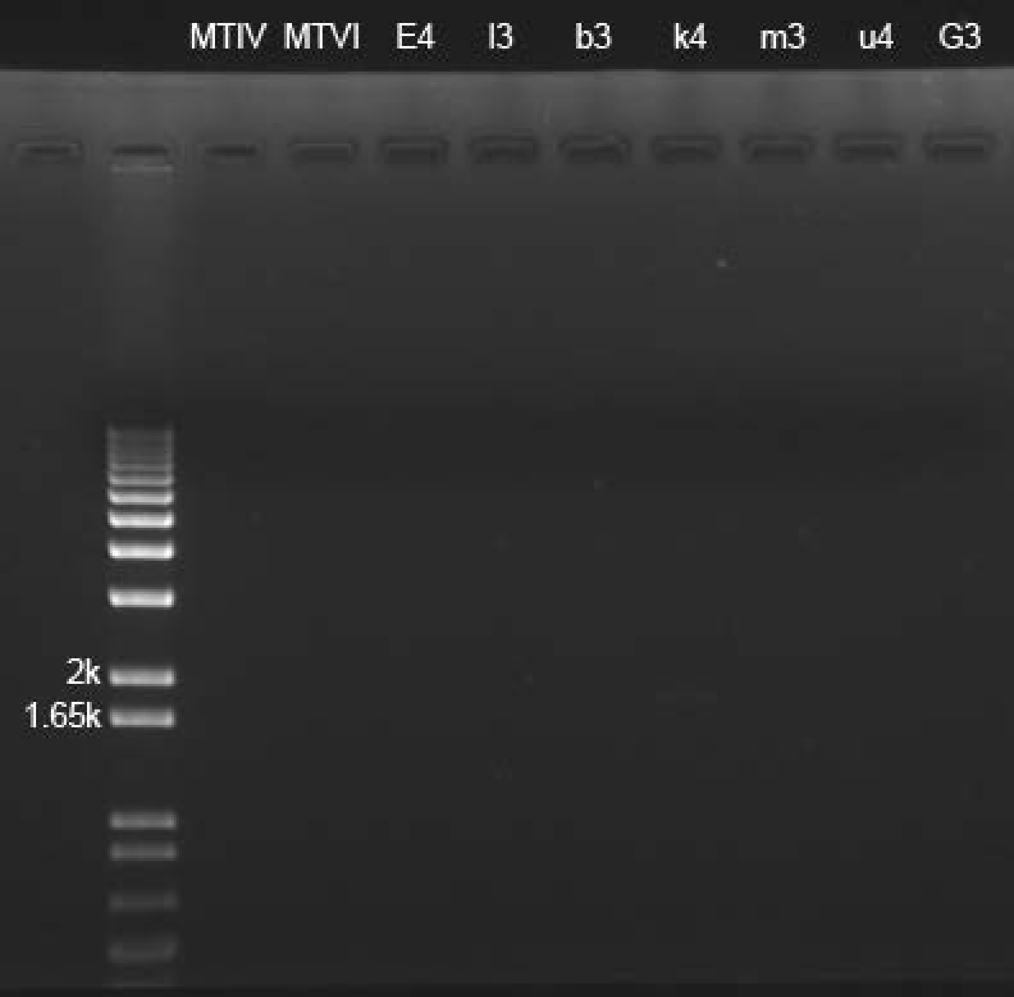

Supp fig S6 RT-PCR MTB6 RT-

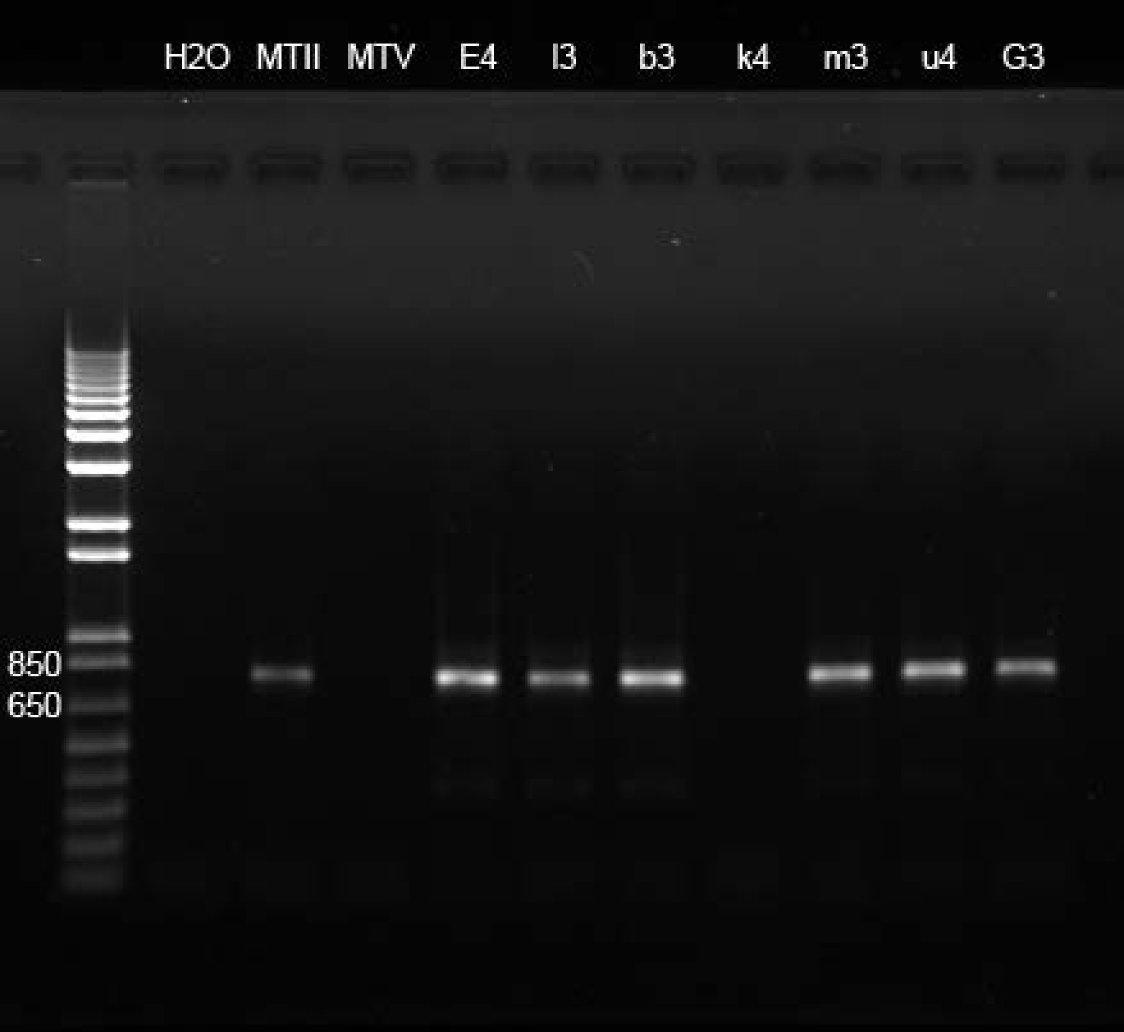

Supp fig S6 RT-PCR MTA2 RT+

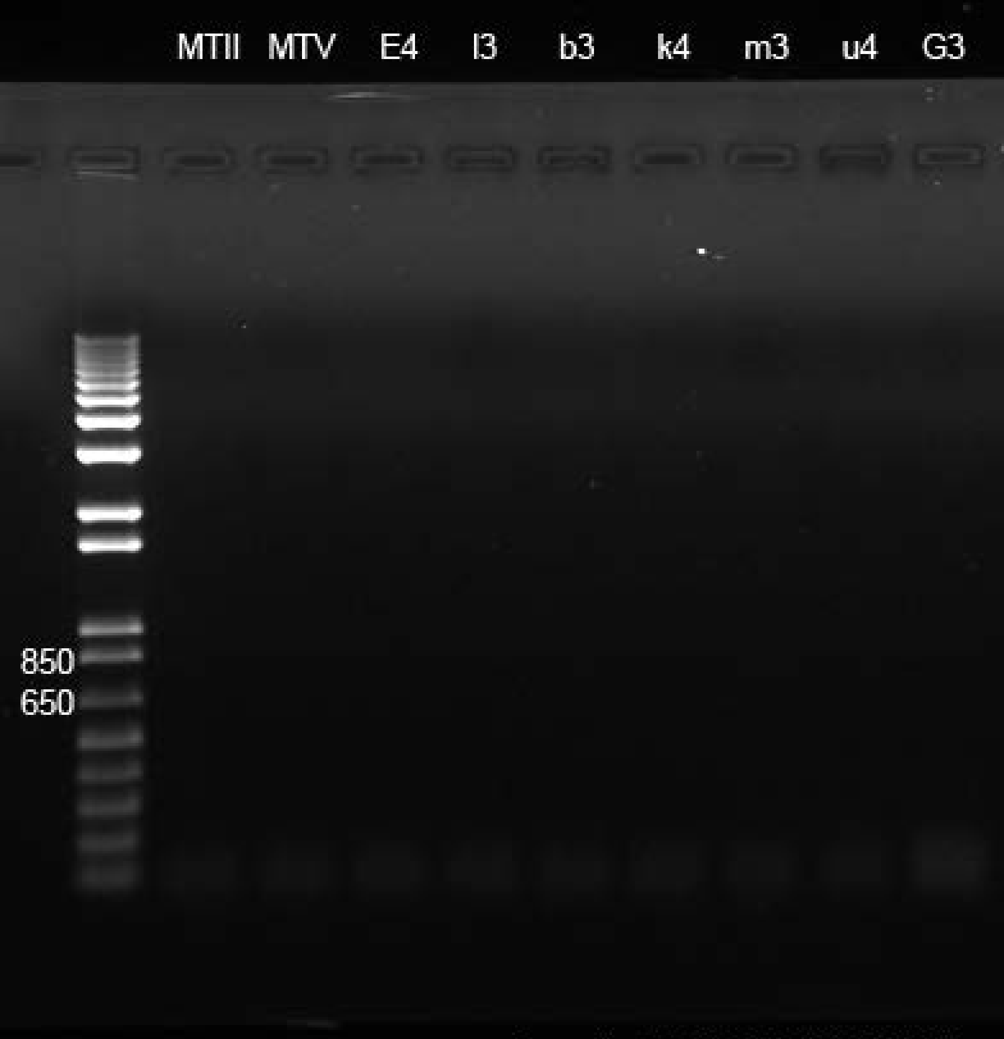

Supp fig S6 RT-PCR MTA2 RT-

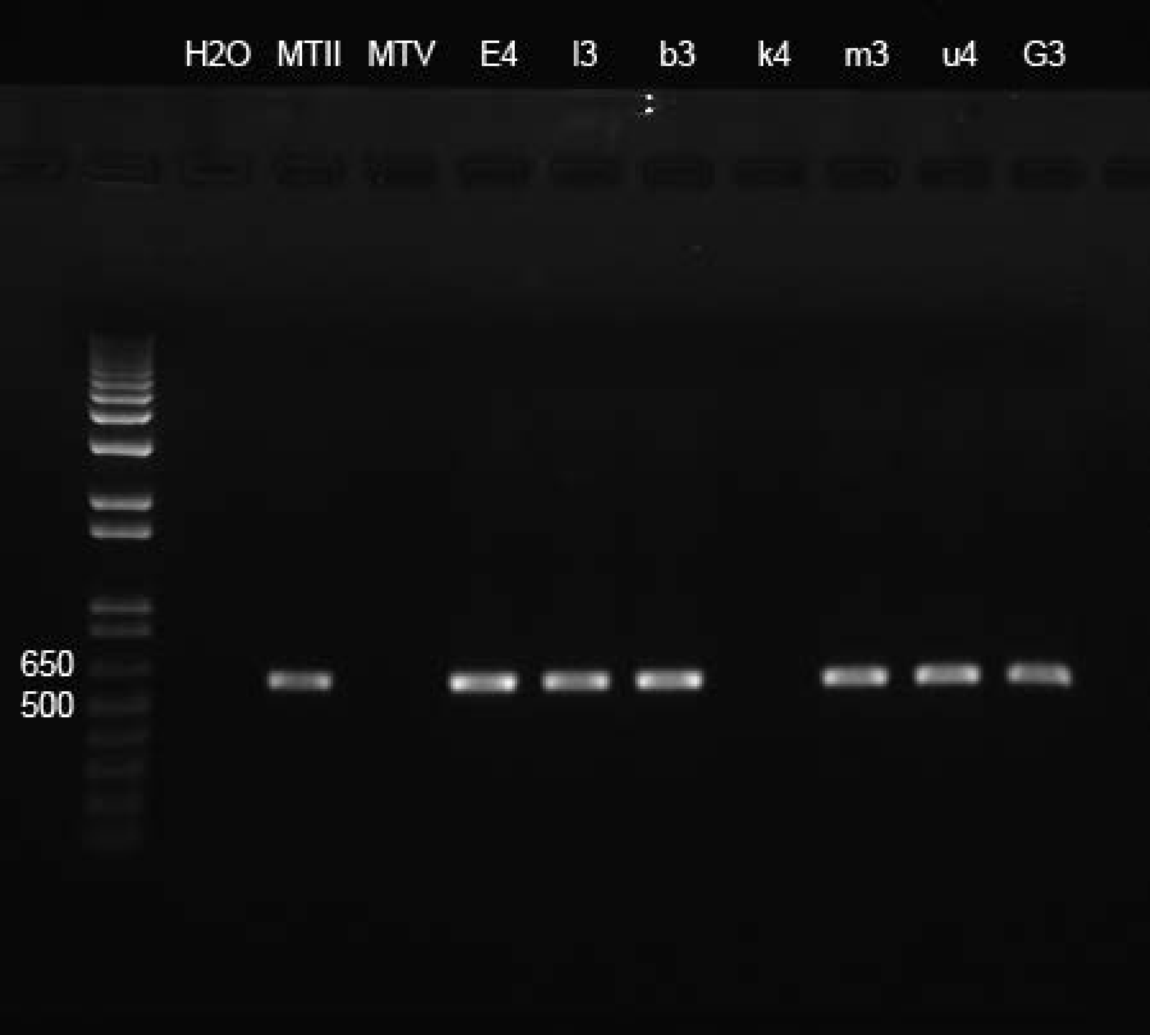

Supp fig S6 RT-PCR MTB2 RT+

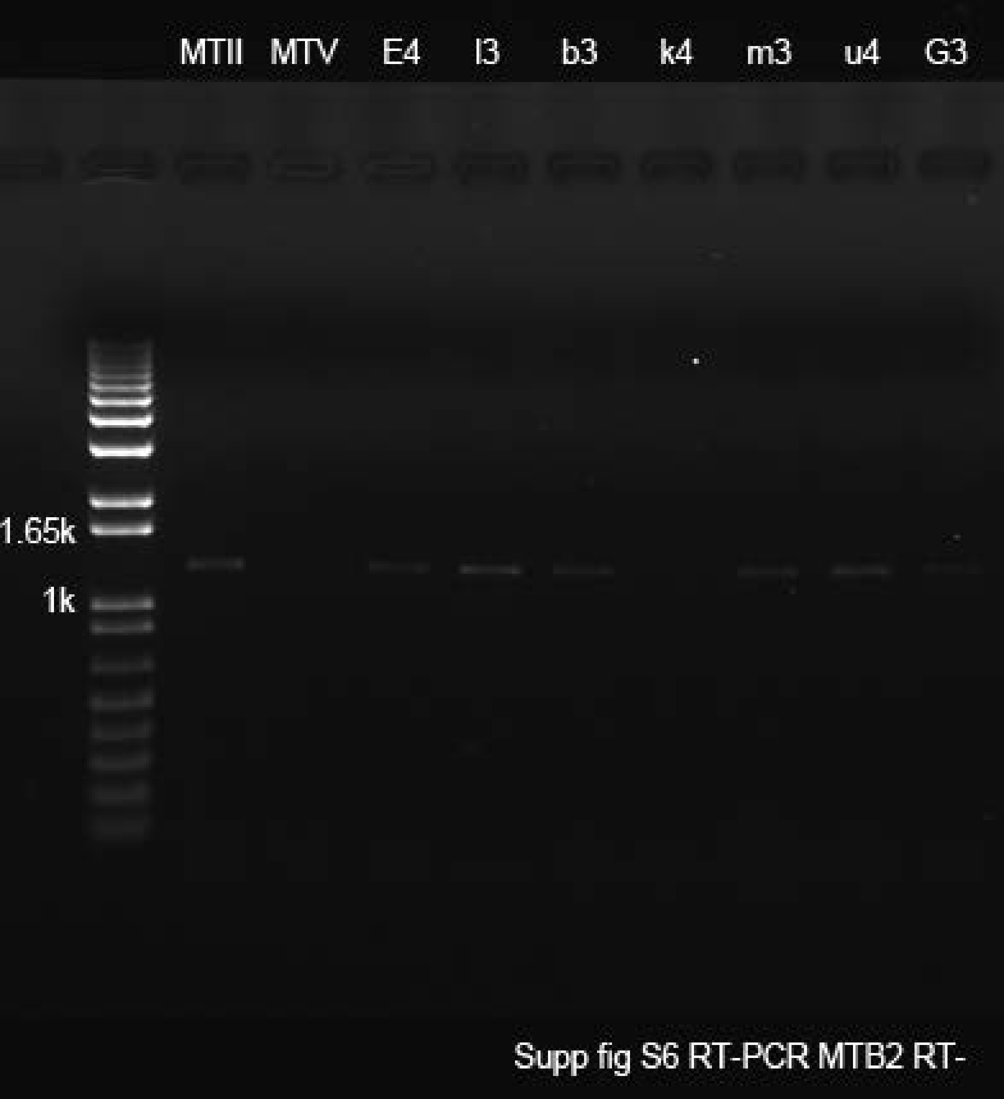
MTII
MTV
E4
I3
b3
k4
m3
u4
G3
1.65k
1k
Supp fig S6 RT-PCR MTB2 RT-

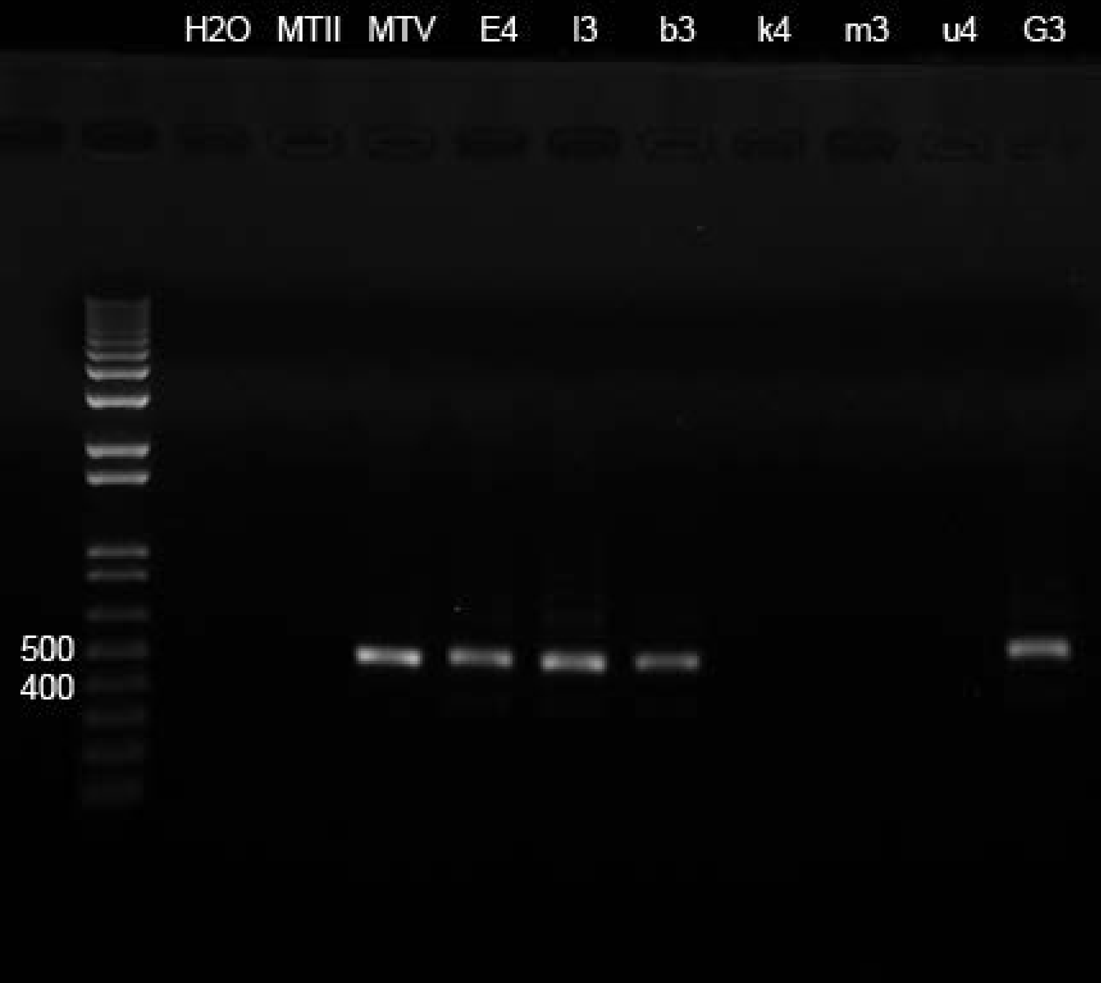

Supp fig S6 RT-PCR MTA5 RT+

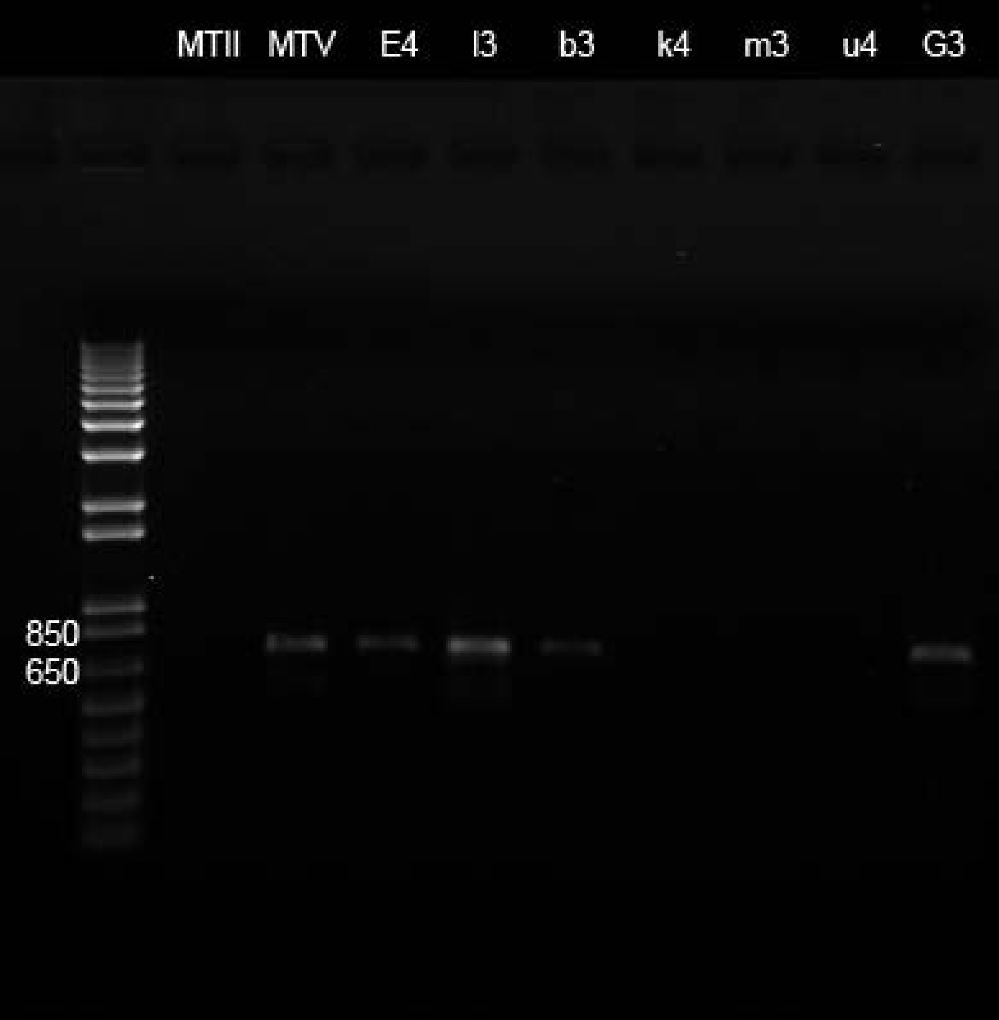

Supp fig S6 RT-PCR MTA5 RT-

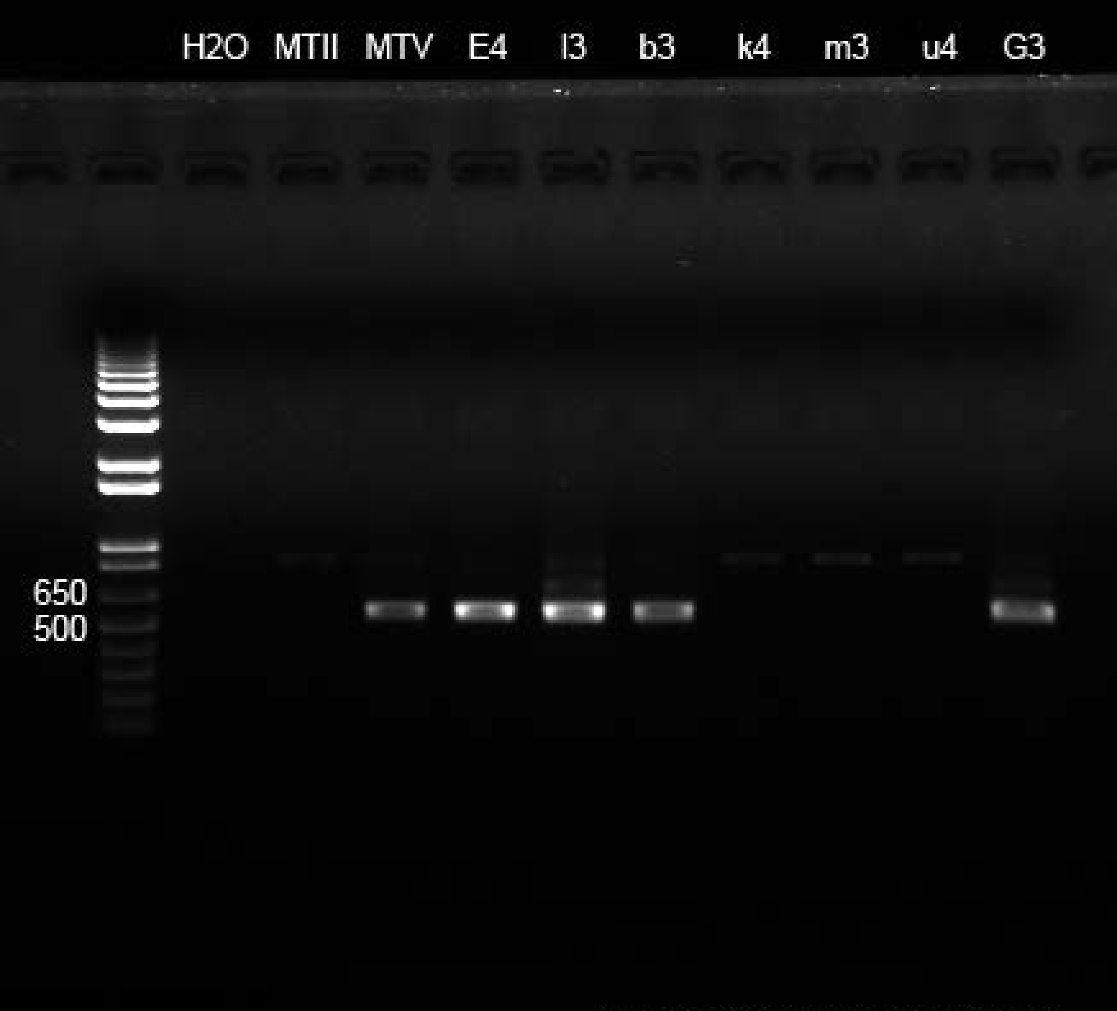

Supp fig S6 RT-PCR MTB5 RT+

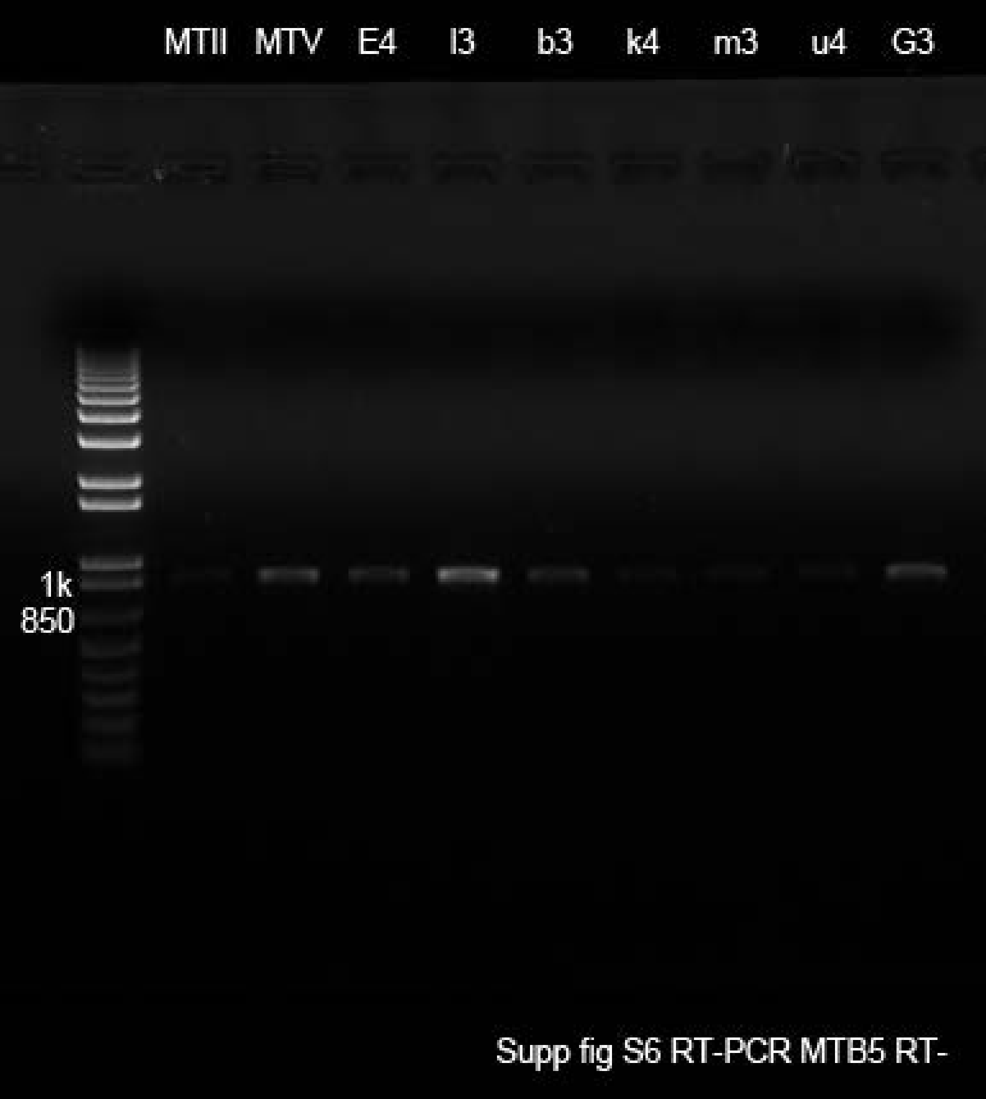
MTII
MTV
E4
I3
b3
k4
m3
u4
G3
1k
850
Supp fig S6 RT-PCR MTB5 RT-

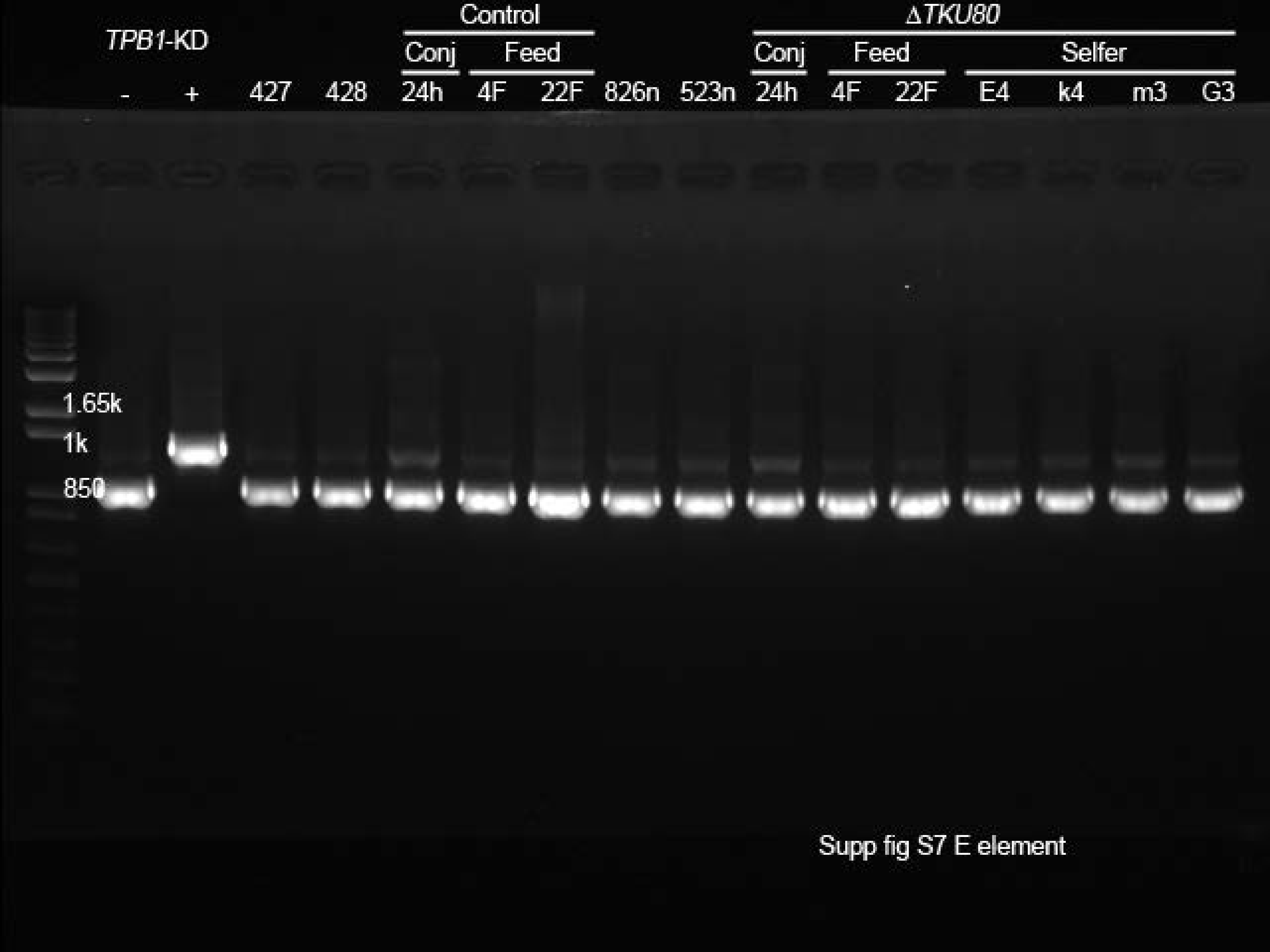
TPB1-KD
-
+
427
428
Control
Conj
24h
Feed
4F
22F
826n
523n
ΔTKU80
Conj
24h
Feed
4F
22F
Selfer
E4
k4
m3
G3
1.65k
1k
850
Supp fig S7 E element

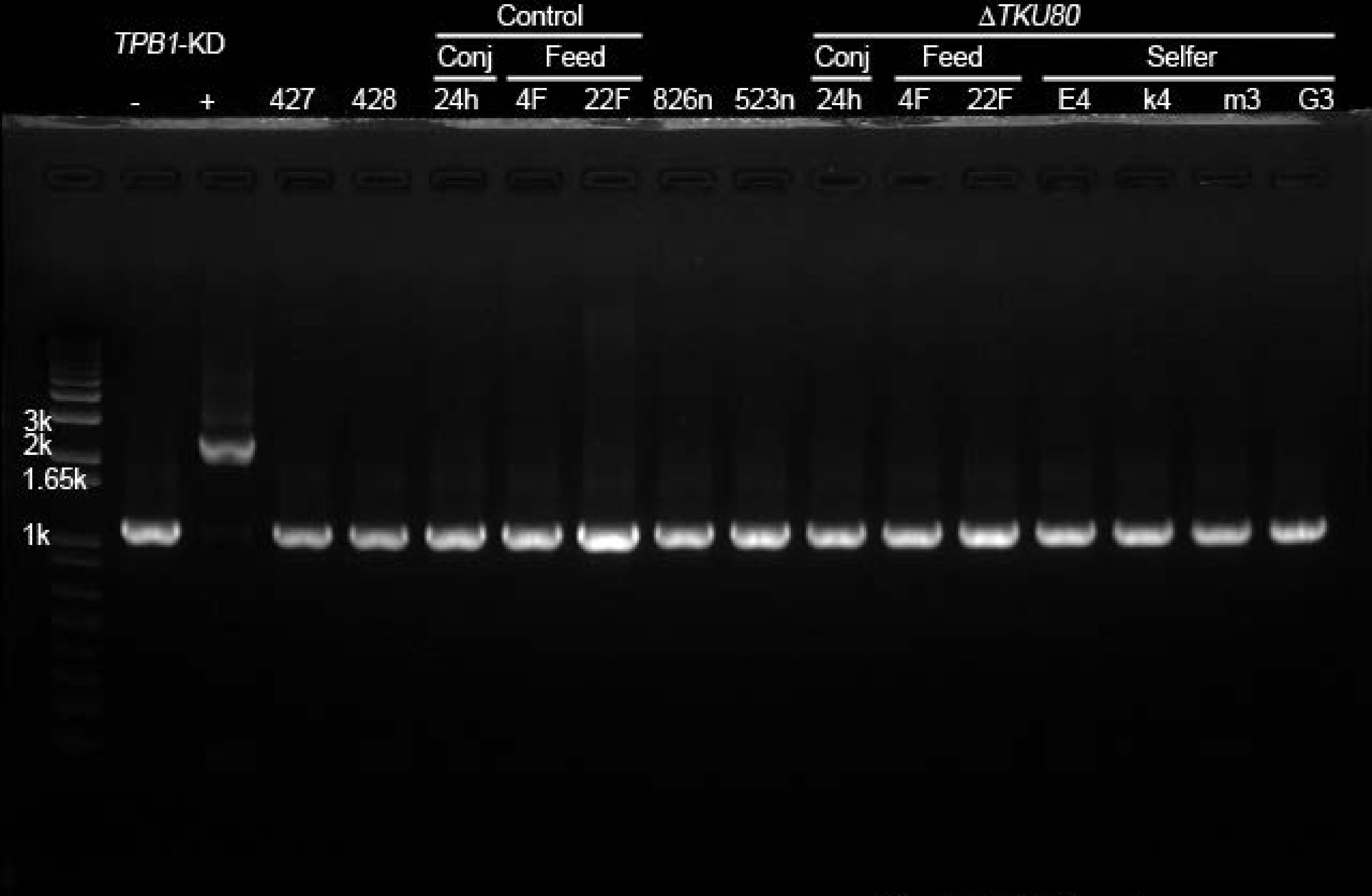

Supp fig S7 G element

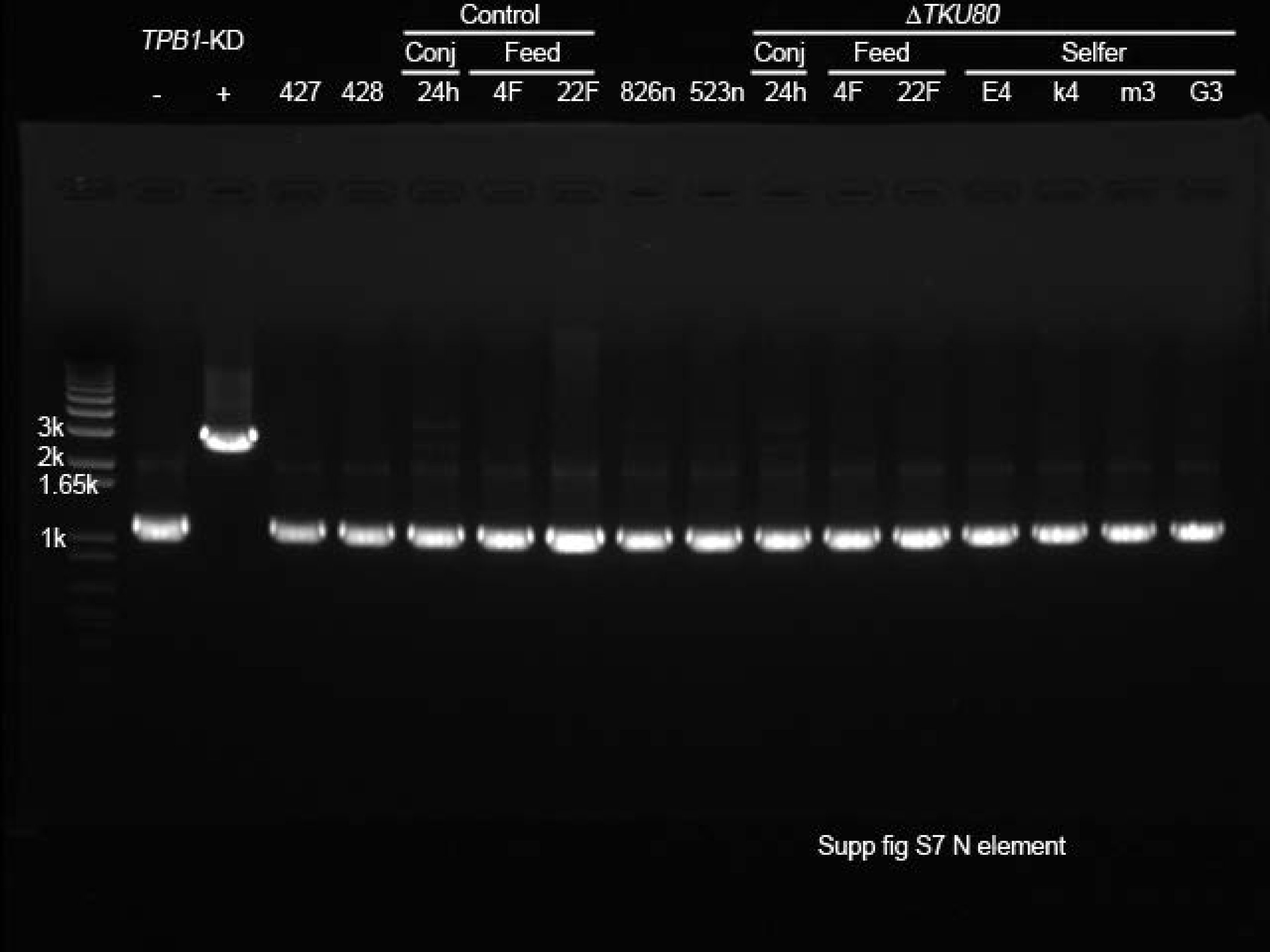
TPB1-KD
Control
ΔTKU80
Conj
Feed
Conj
Feed
Selfer
-
+
427
428
24h
4F
22F
826n
523n
24h
4F
22F
E4
k4
m3
G3
3k
2k
1.65k
1k
Supp fig S7 N element

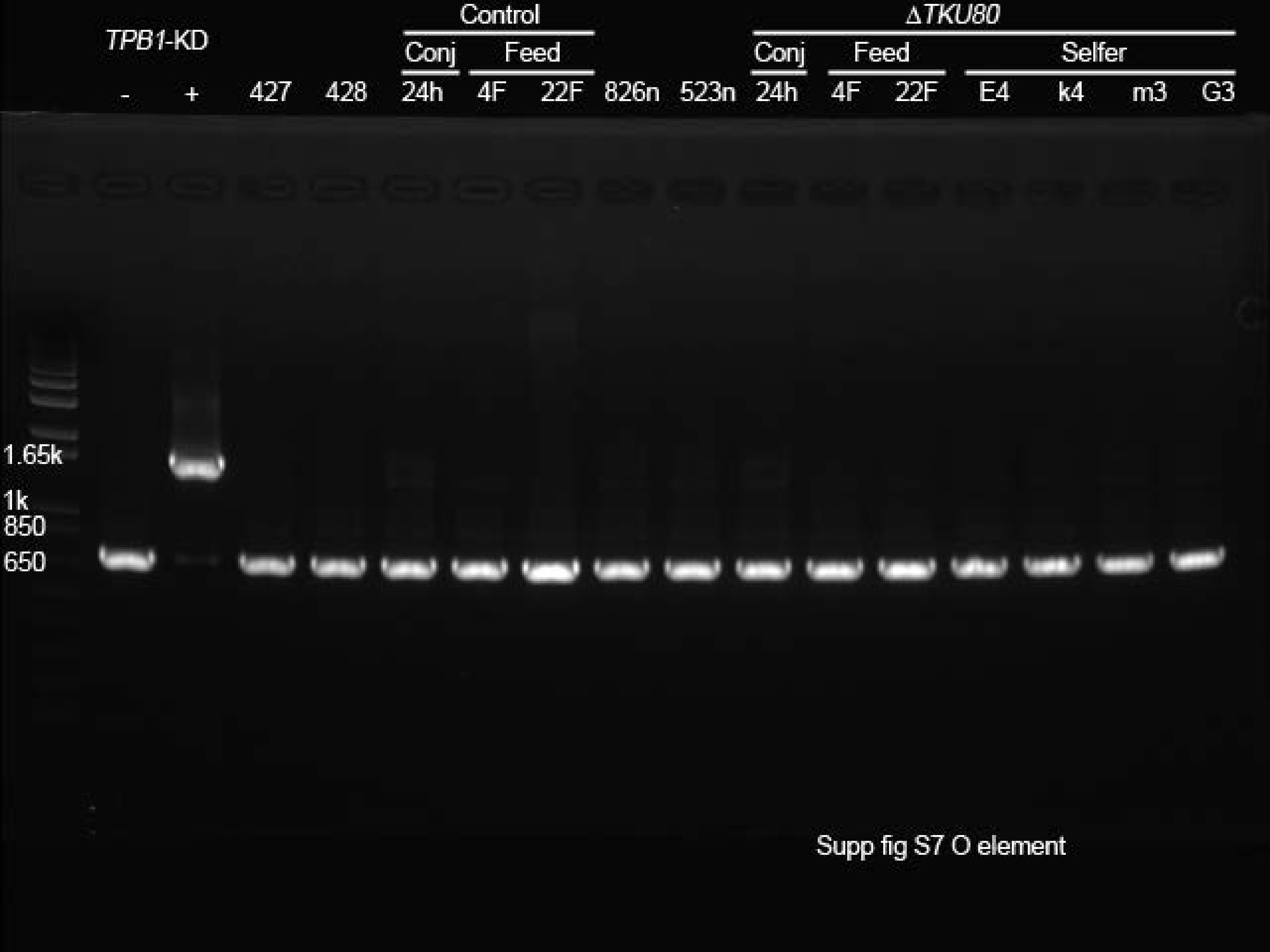
TPB1-KD
Control
ΔTKU80
Conj
Feed
Conj
Feed
Selfer
-
+
427
428
24h
4F
22F
826n
523n
24h
4F
22F
E4
k4
m3
G3
1.65k
1k
850
650
Supp fig S7 O element

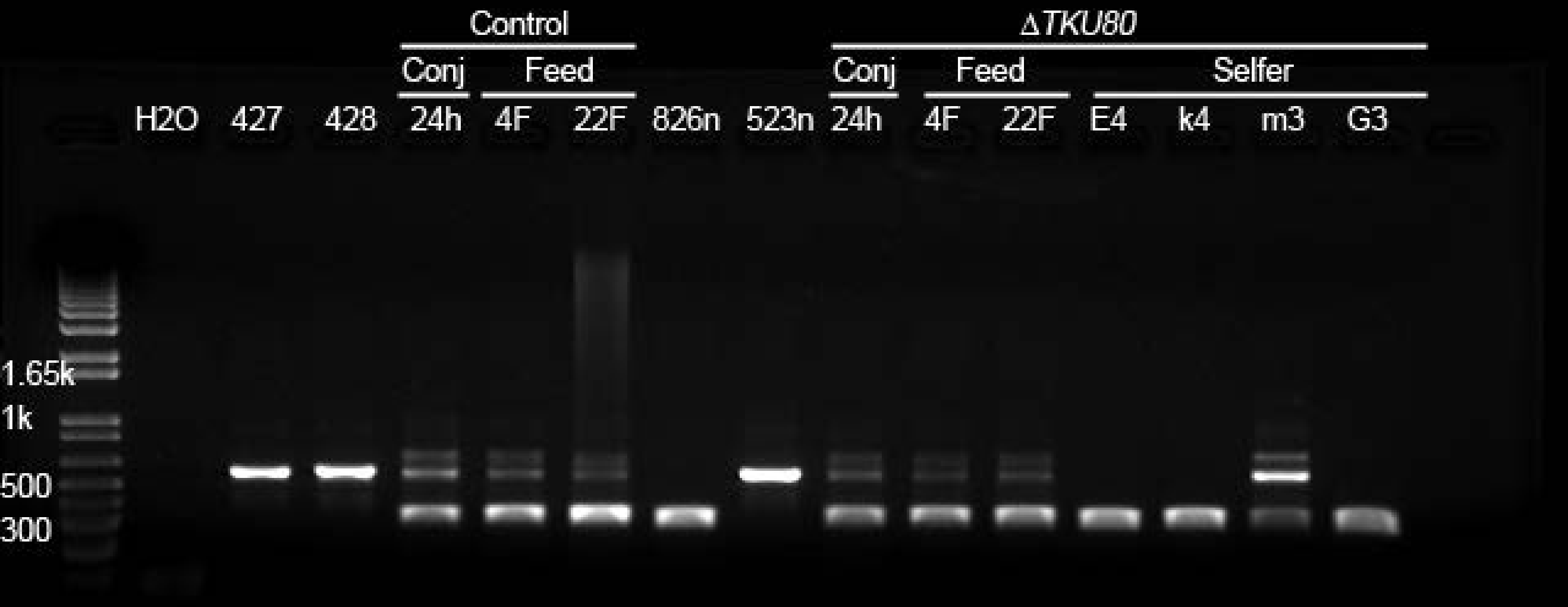

Supp fig S7 M element

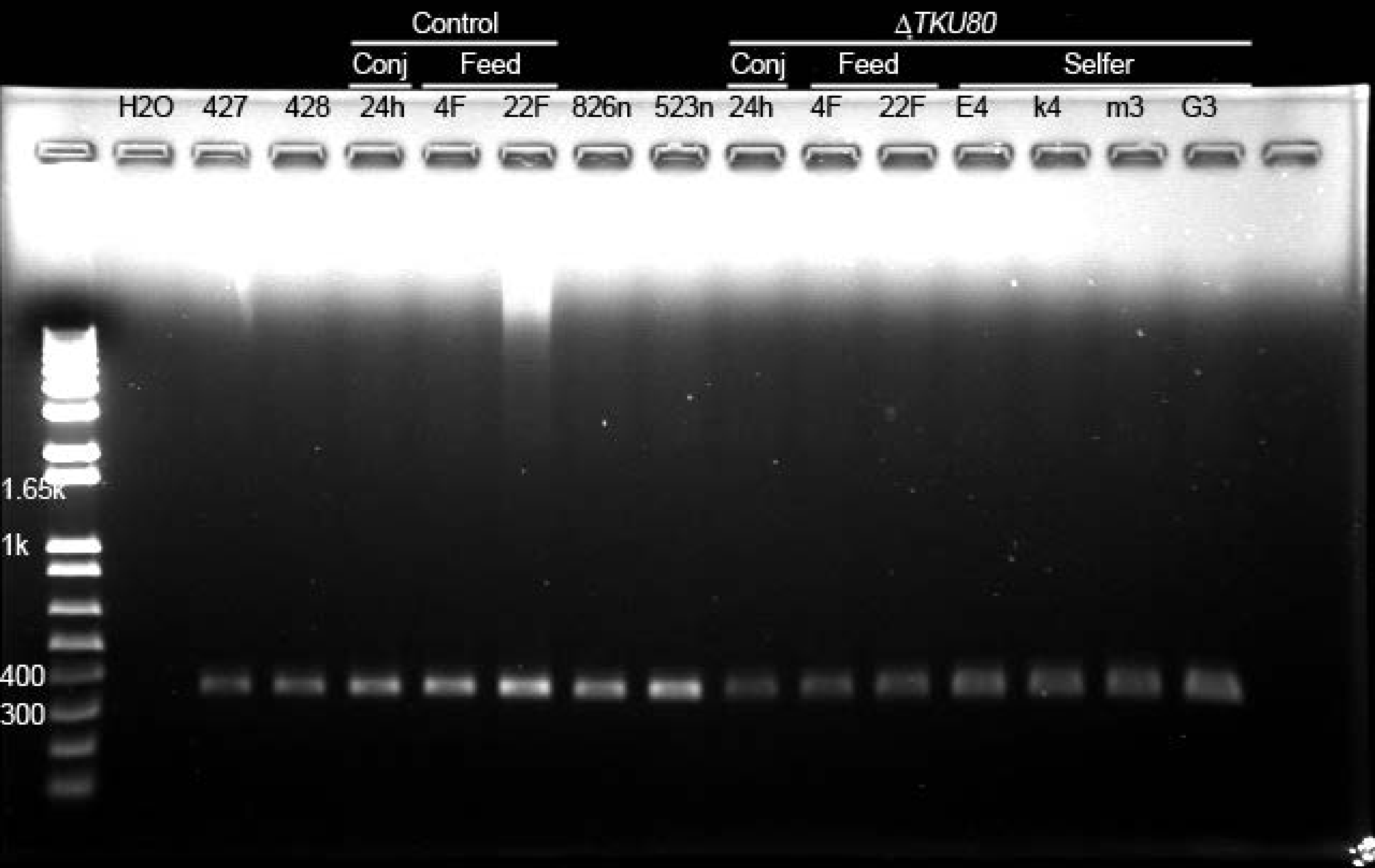

Supp fig S7 R element

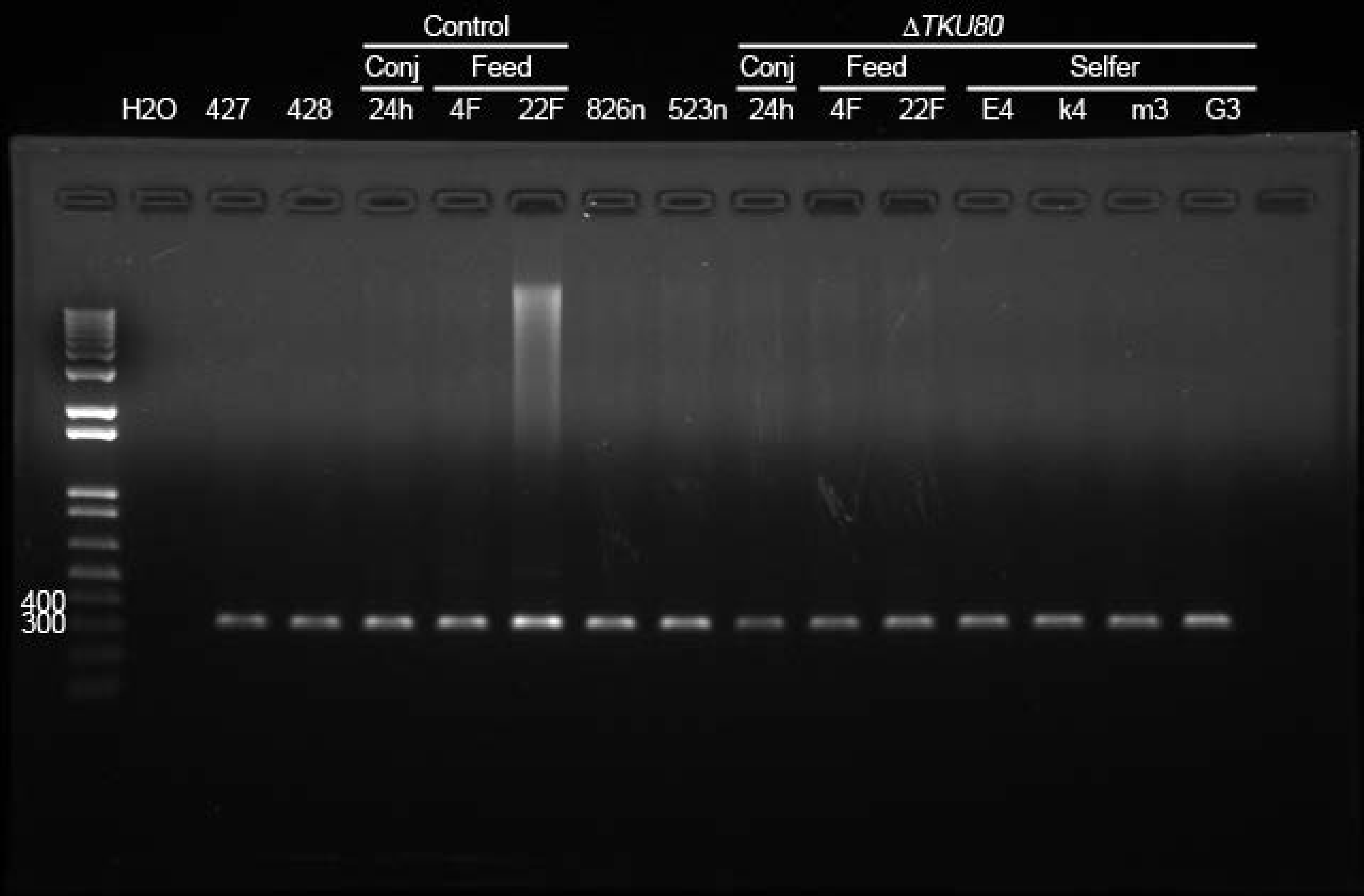

Supp fig S7 Cam element

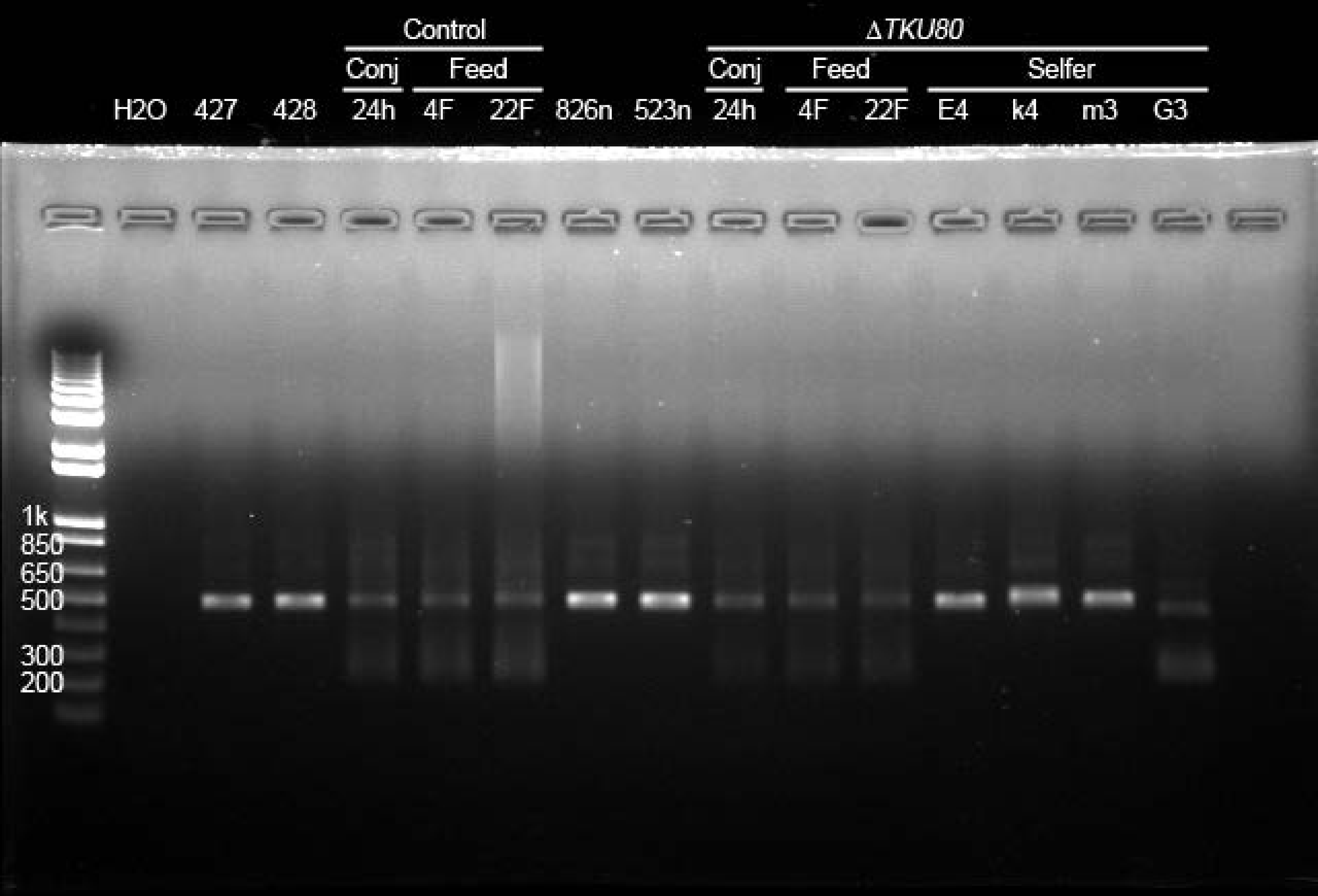

Supp fig S7 Tlr-1 element

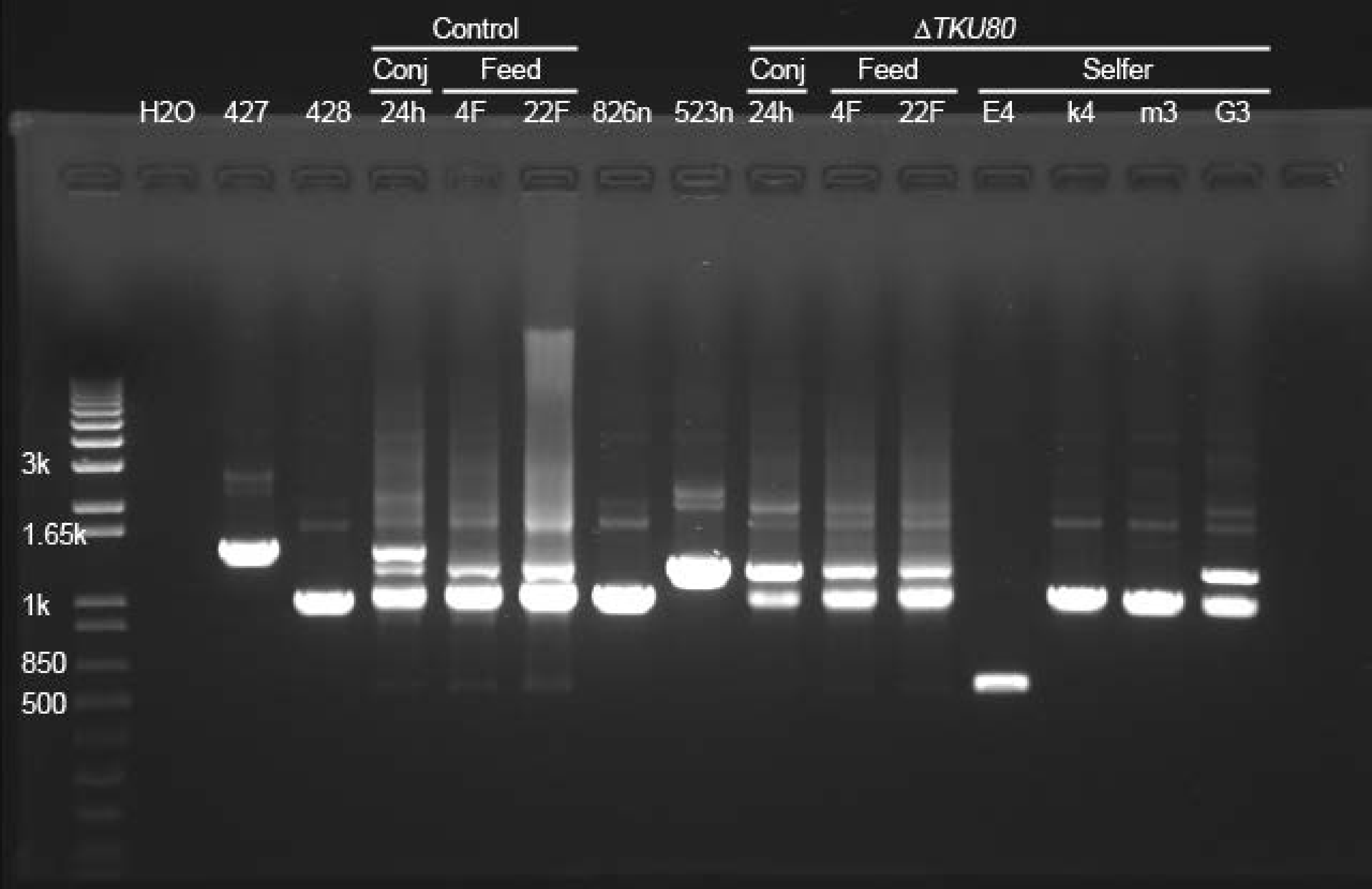

Supp fig S7 Rdn element

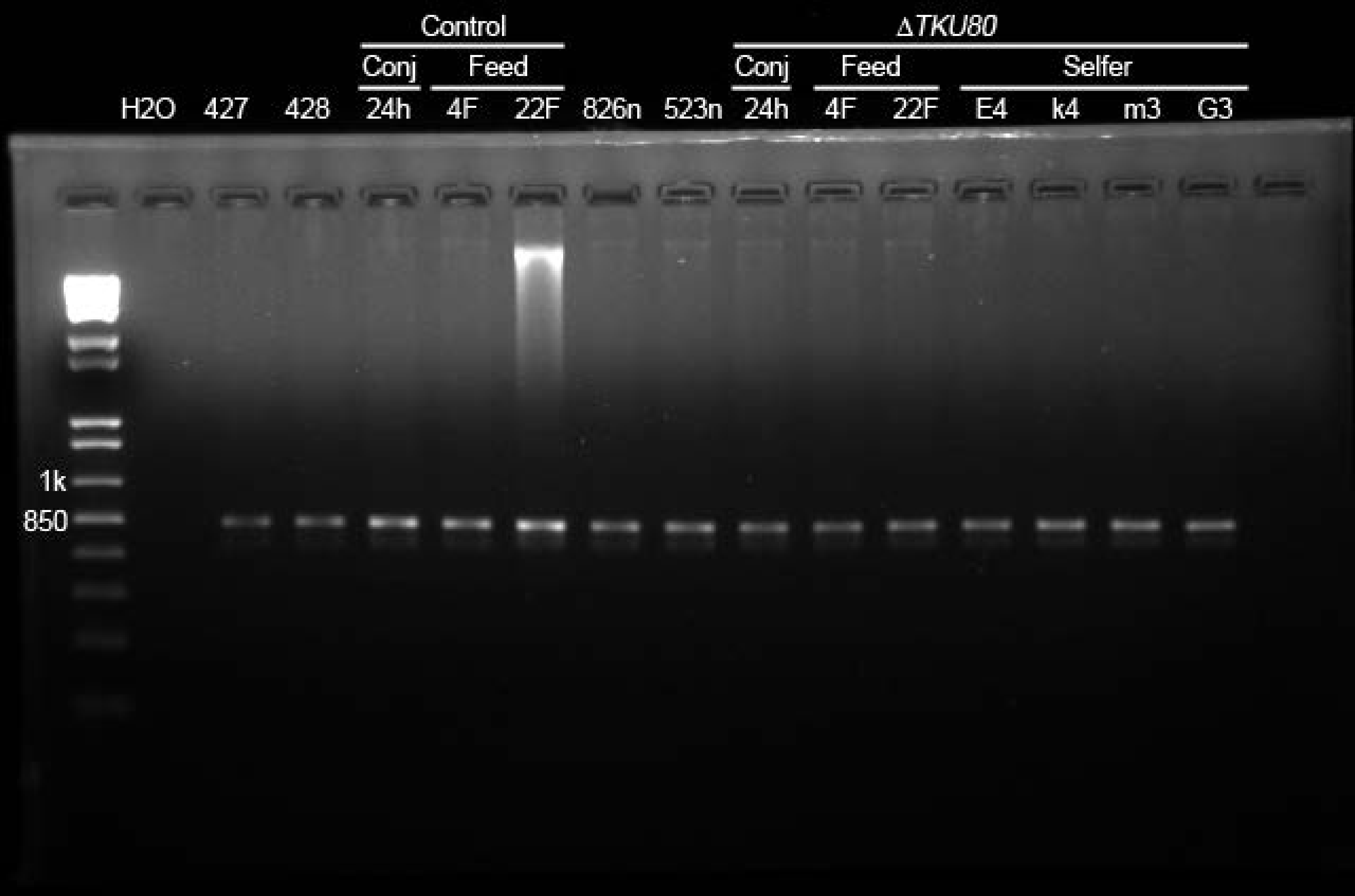

Supp fig S7 Cbs819 Mic

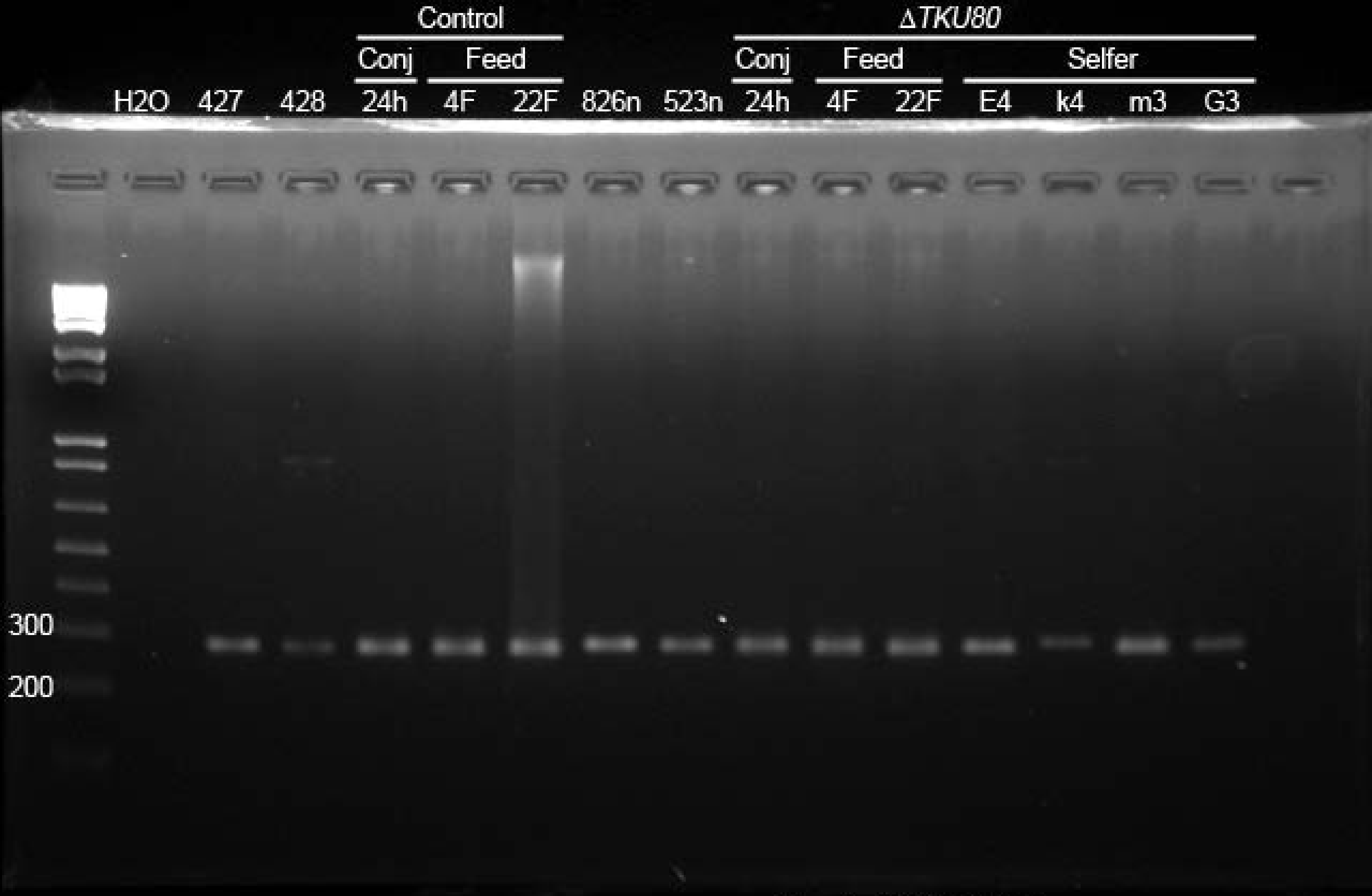

Supp fig S7 Cbs819 Mac

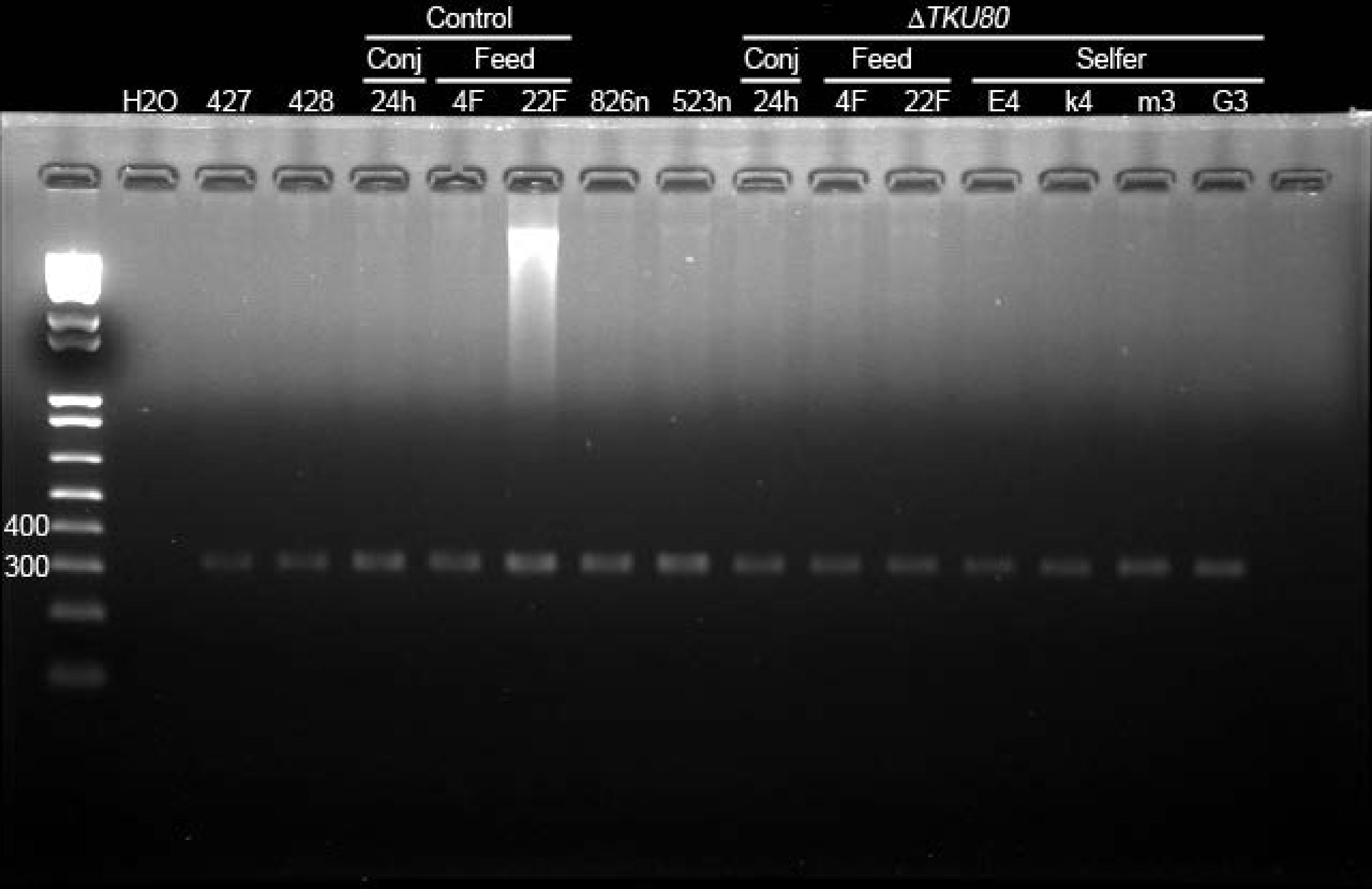

Supp fig S7 Cbs826 Mic

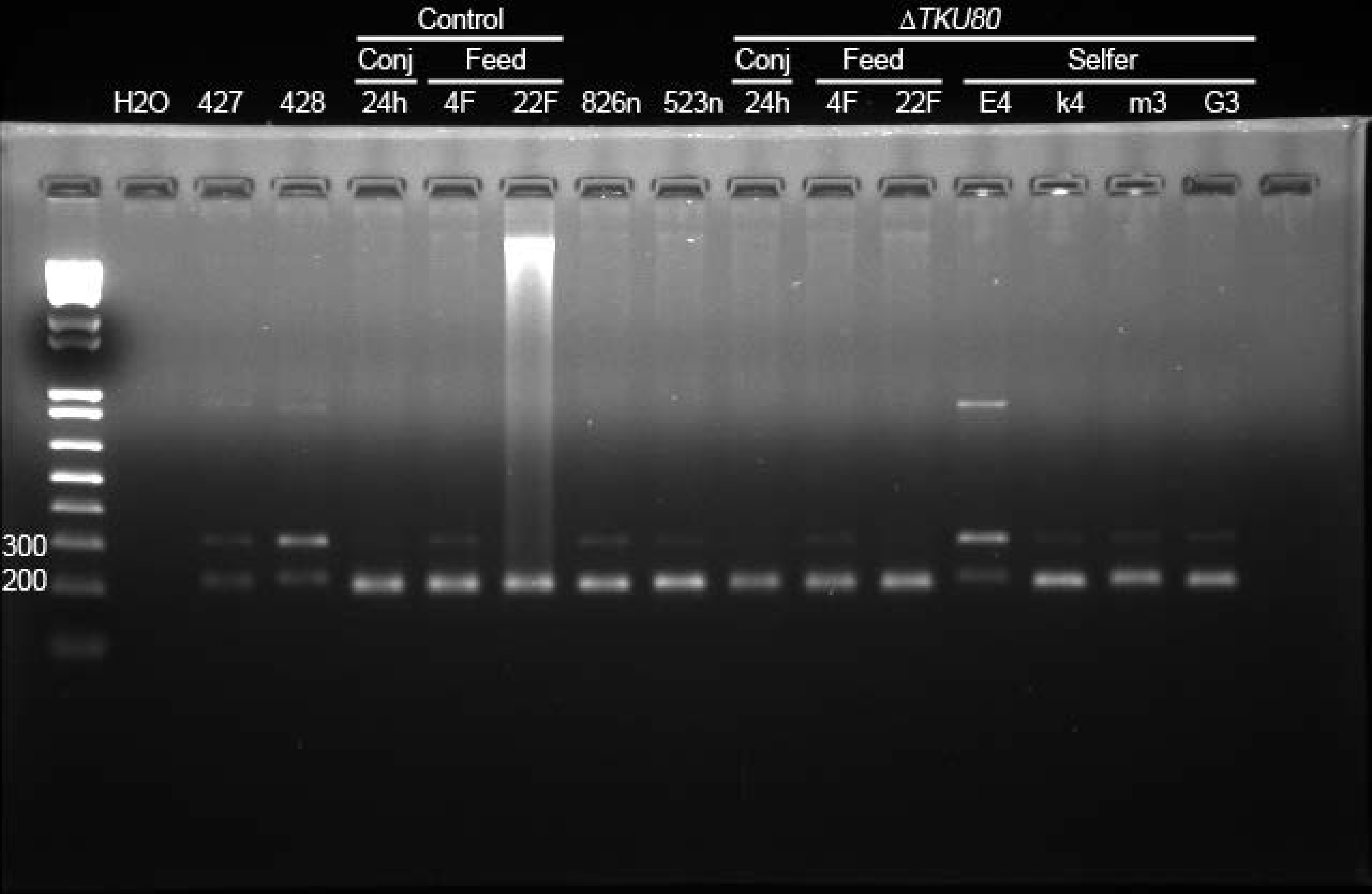

Supp fig S7 Cbs826 Mac

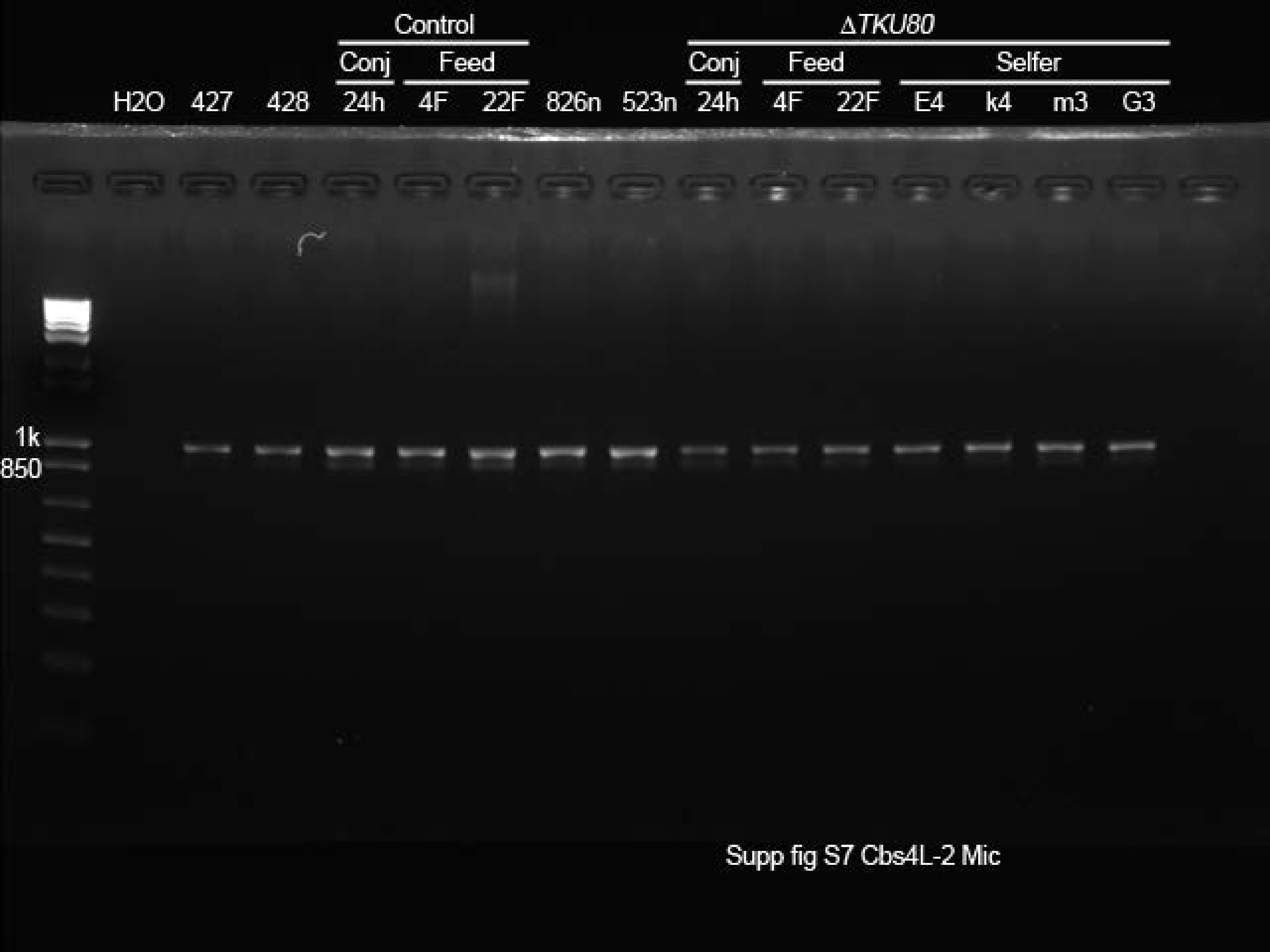
Control
ΔTKU80
Conj
Feed
Conj
Feed
Selfer
H2O
427
428
24h
4F
22F
826n
523n
24h
4F
22F
E4
k4
m3
G3
1k
850
Supp fig S7 Cbs4L-2 Mic

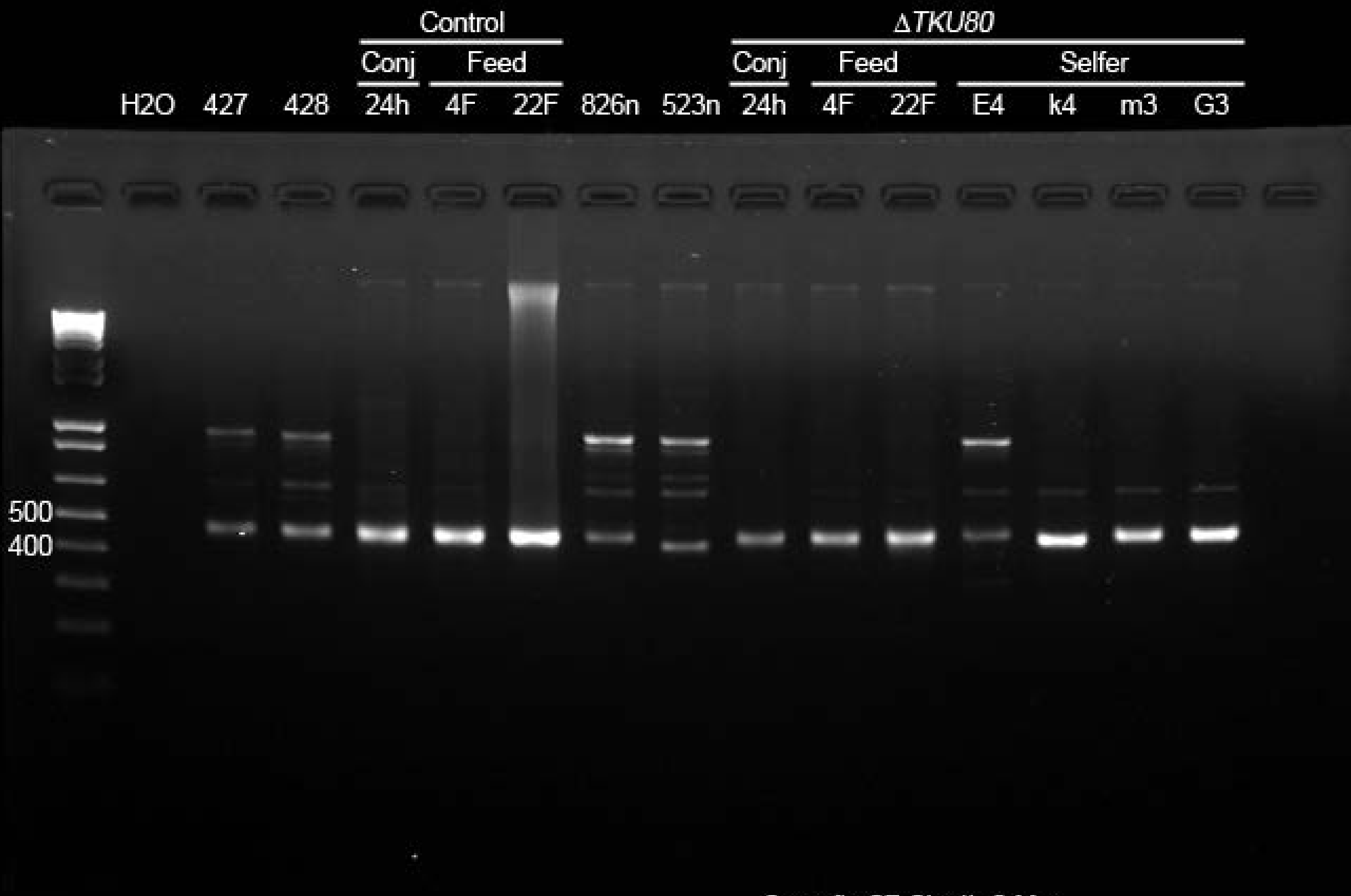

Supp fig S7 Cbs4L-2 Mac

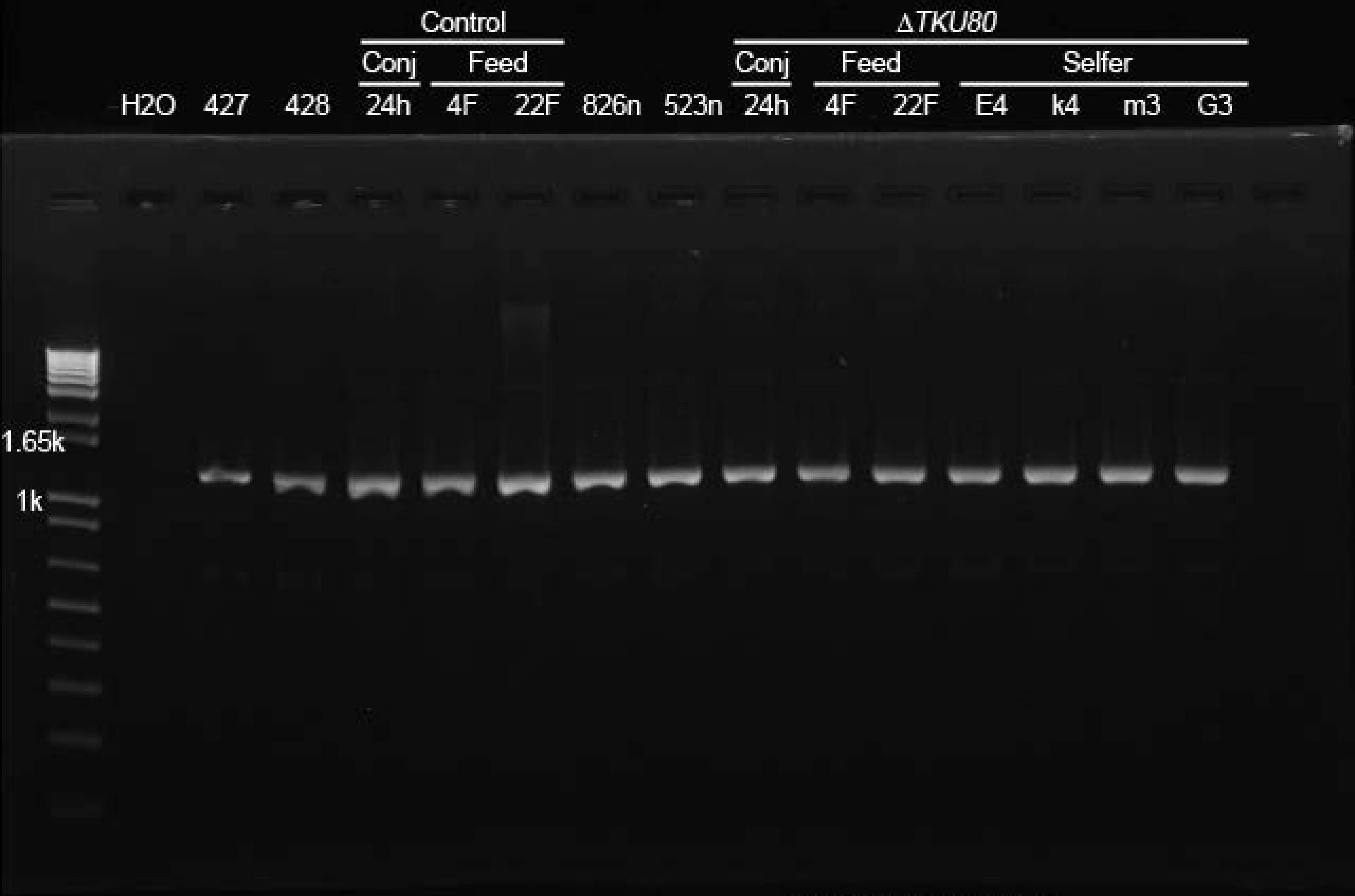

Supp fig S7 Cbs5-2 Mic

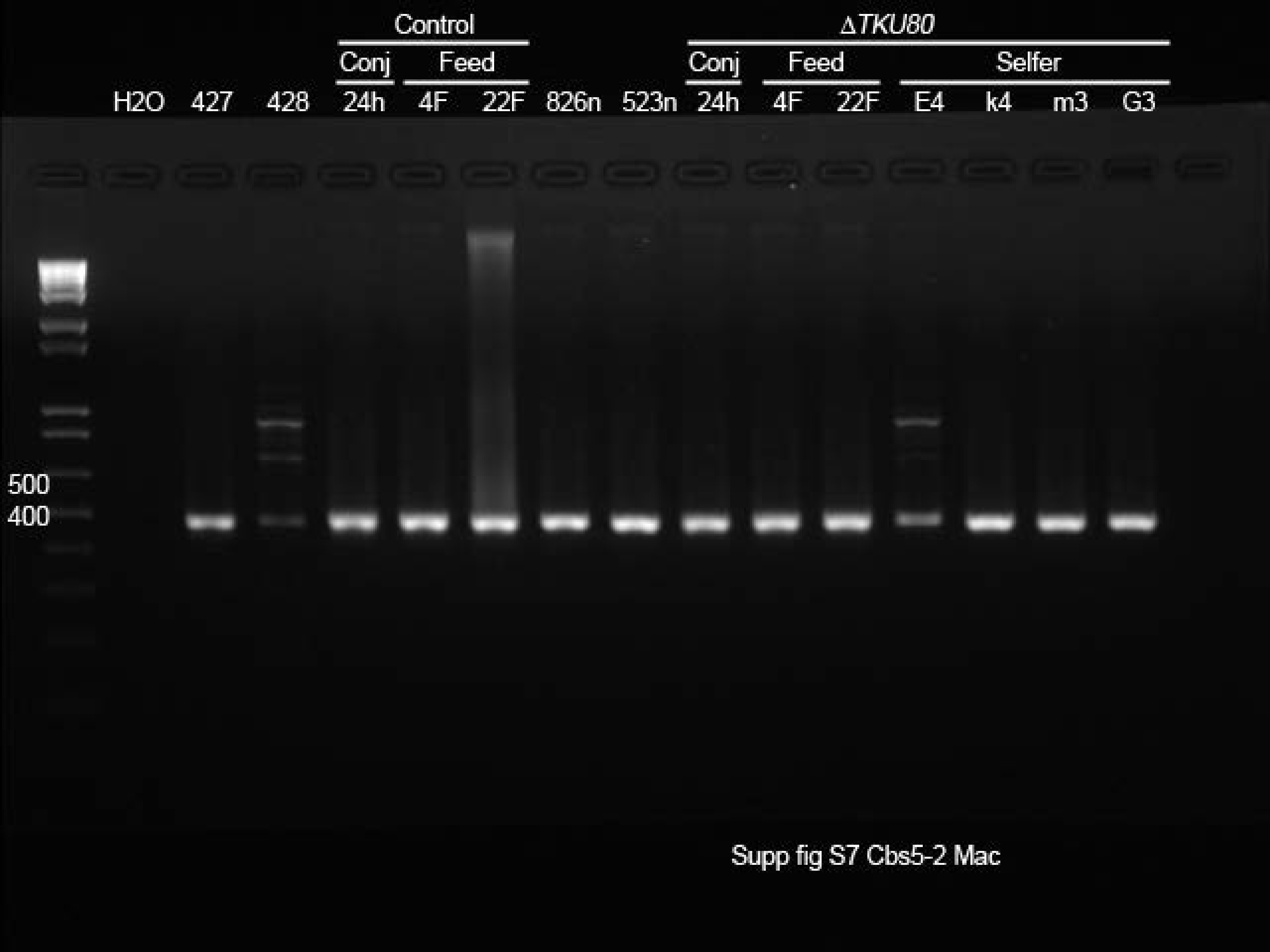
Control
Conj
Feed
ΔTKU80
Conj
Feed
Selfer
H2O
427
428
24h
4F
22F
826n
523n
24h
4F
22F
E4
k4
m3
G3
500
400
Supp fig S7 Cbs5-2 Mac

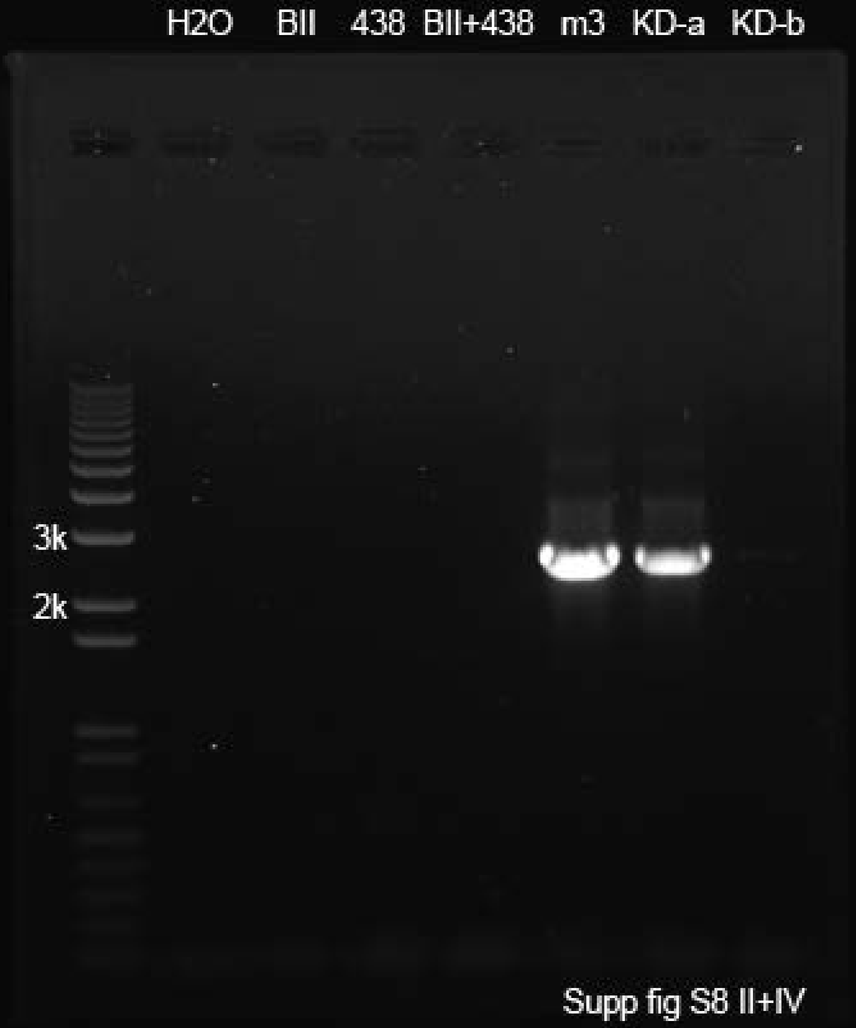
H2O
BII
438
BII+438
m3
KD-a
KD-b
3k
2k
Supp fig S8 II+IV

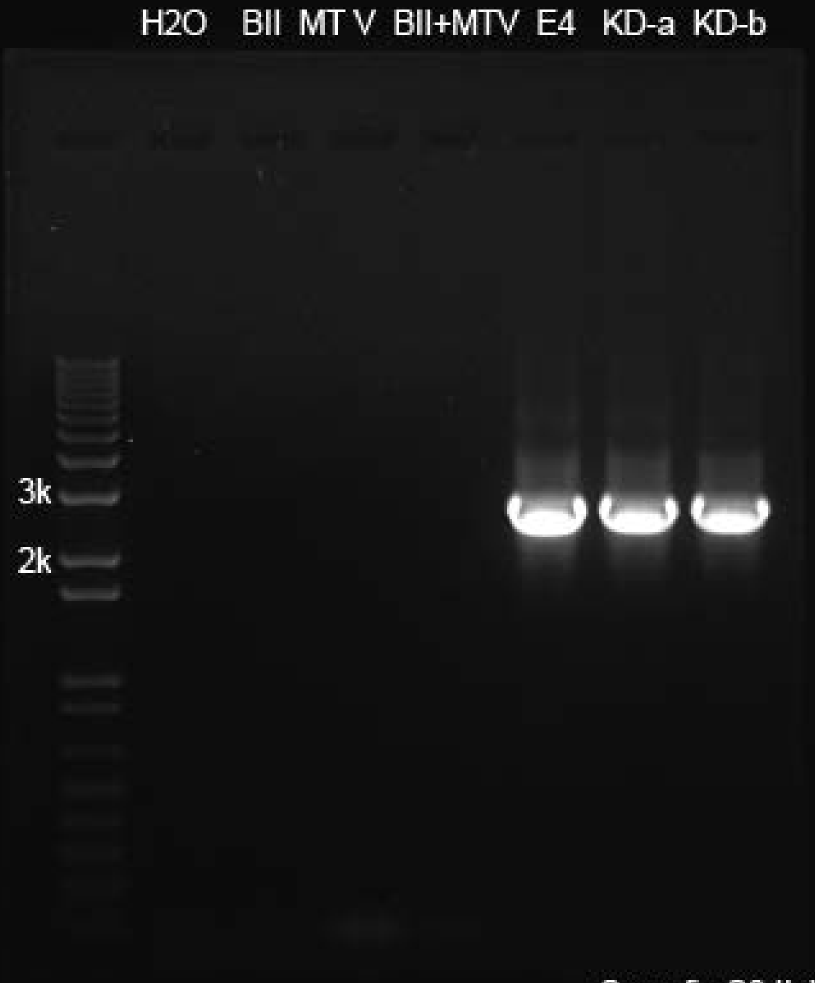

Supp fig S8 II+V

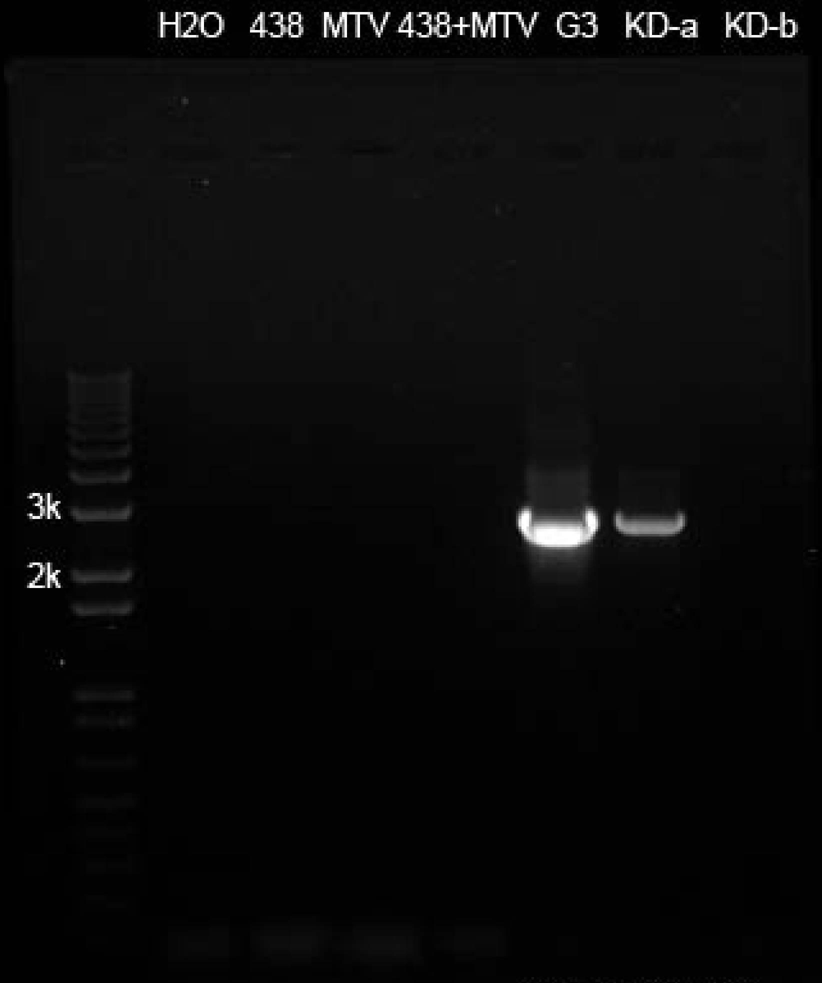

Supp fig S8 IV+V

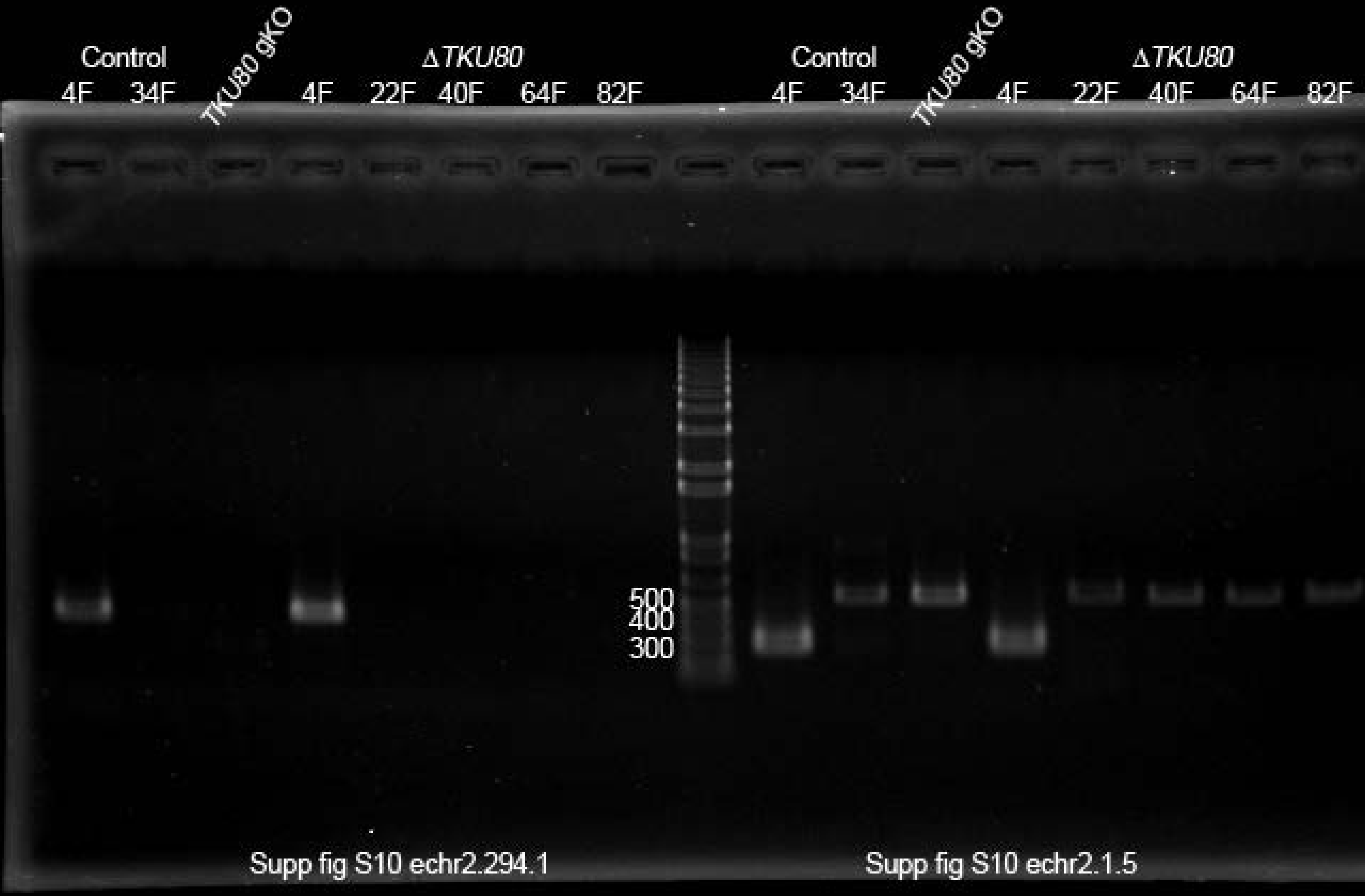
Control
TKU80 gKO
ΔTKU80
4F
34F
4F
22F
40F
64F
82F
Control
TKU80 gKO
ΔTKU80
4F
34F
4F
22F
40F
64F
82F
500
400
300
Supp fig S10 echr2.294.1
Supp fig S10 echr2.1.5

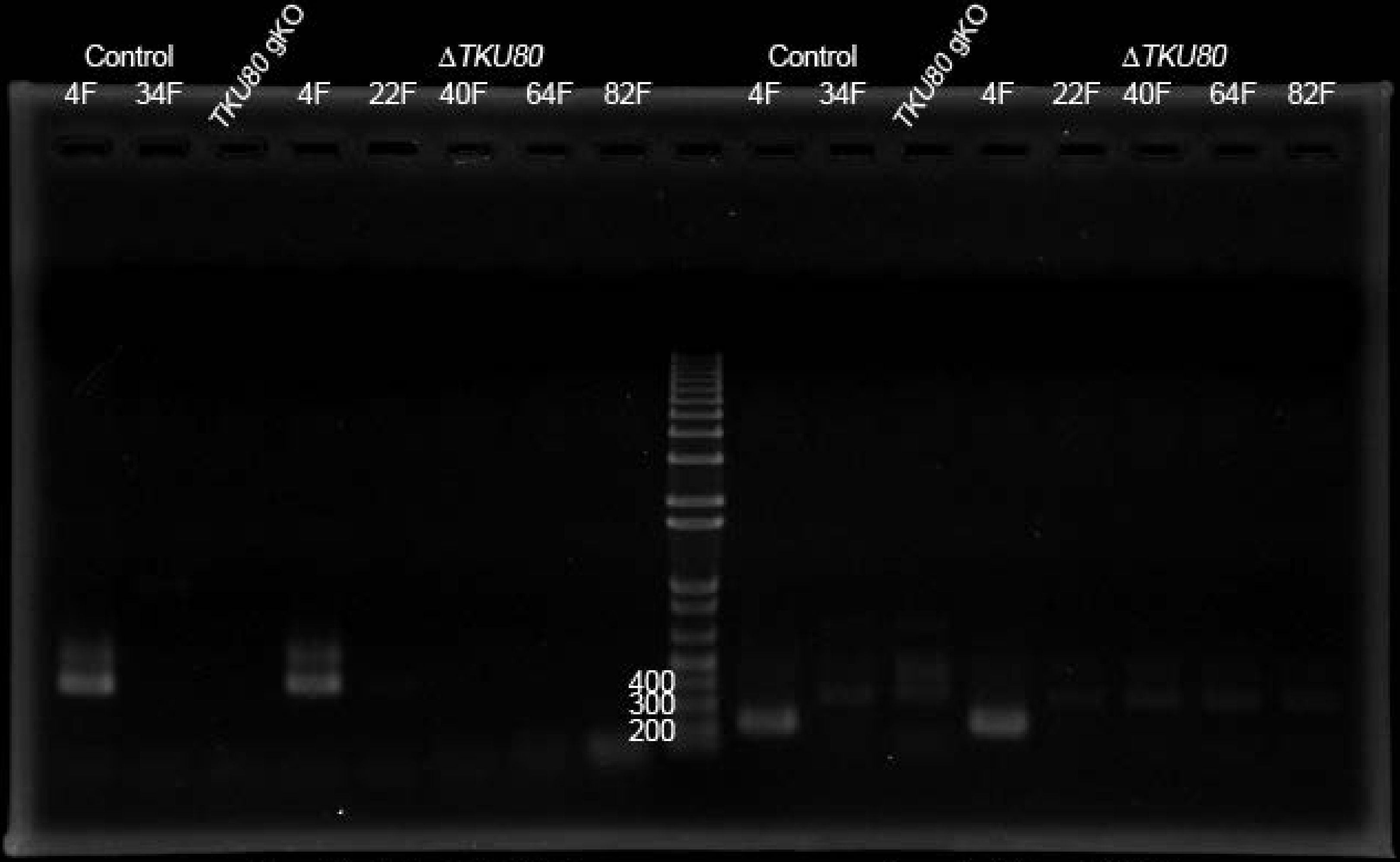

Supp fig S10 echr2.102.1

Supp fig S10 echr2.75.1

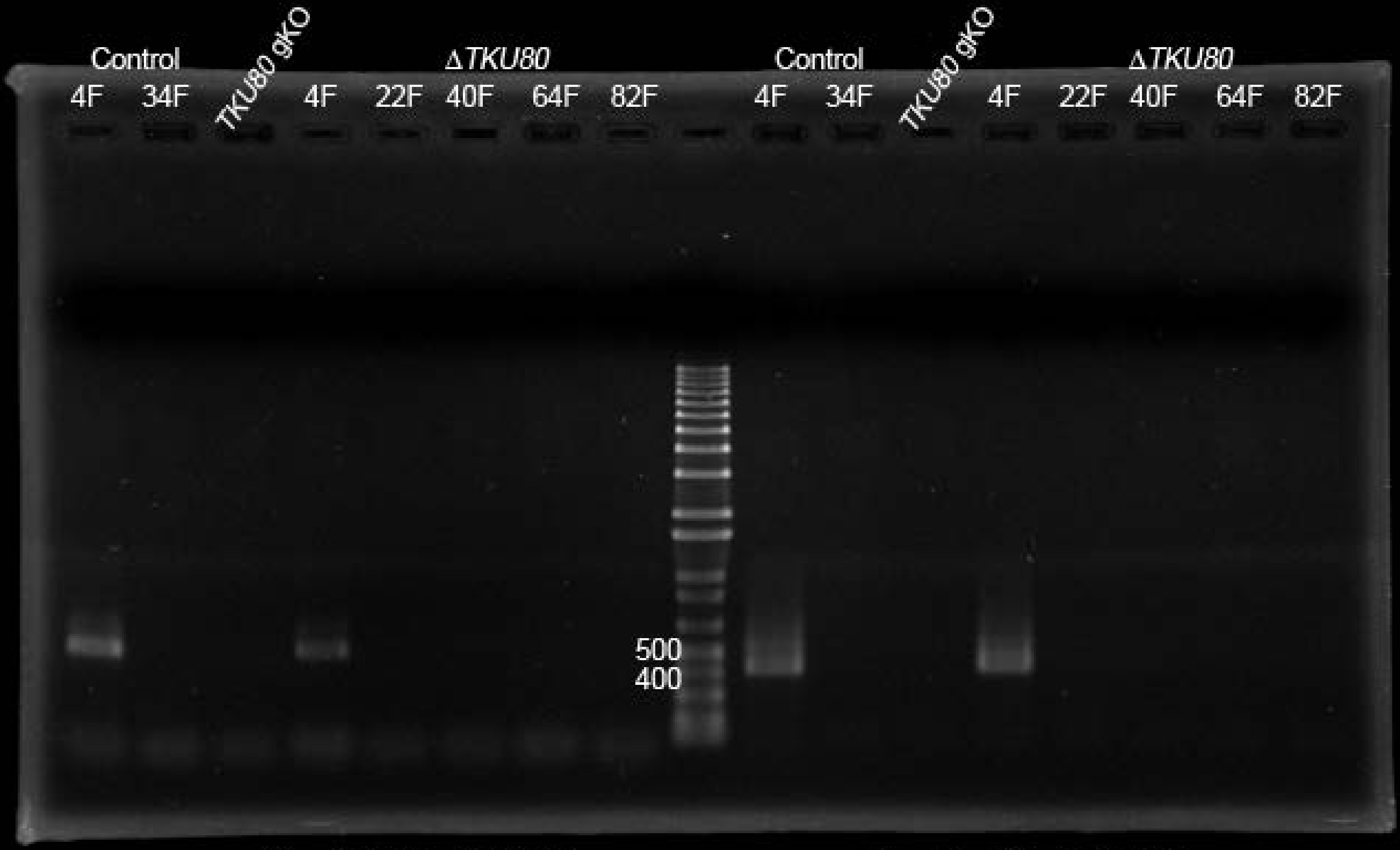

Supp fig S10 echr2.105.1

Supp fig S10 echr2.240.1

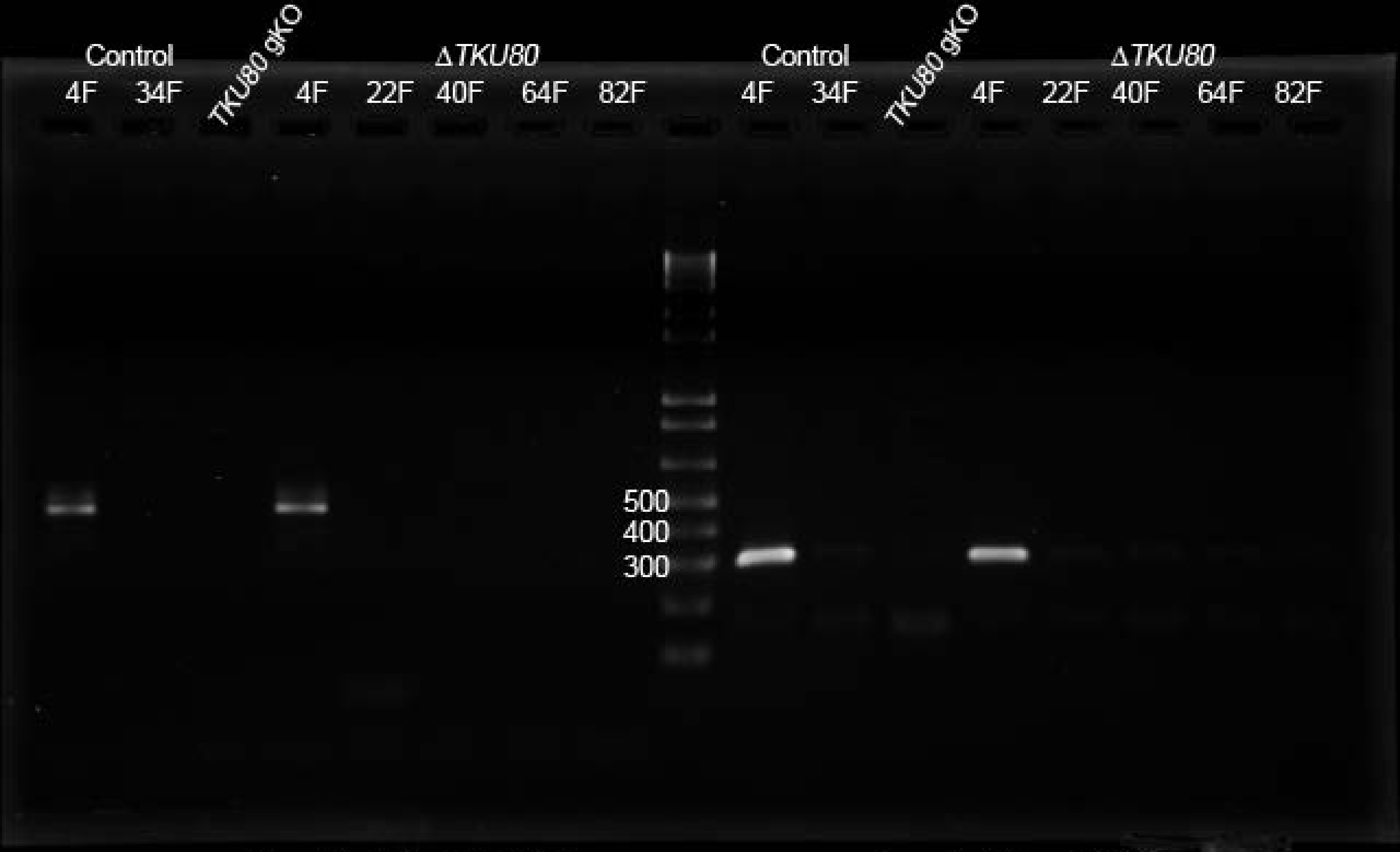

Supp fig S10 echr2.190.1

Supp fig S10 echr2.78.1

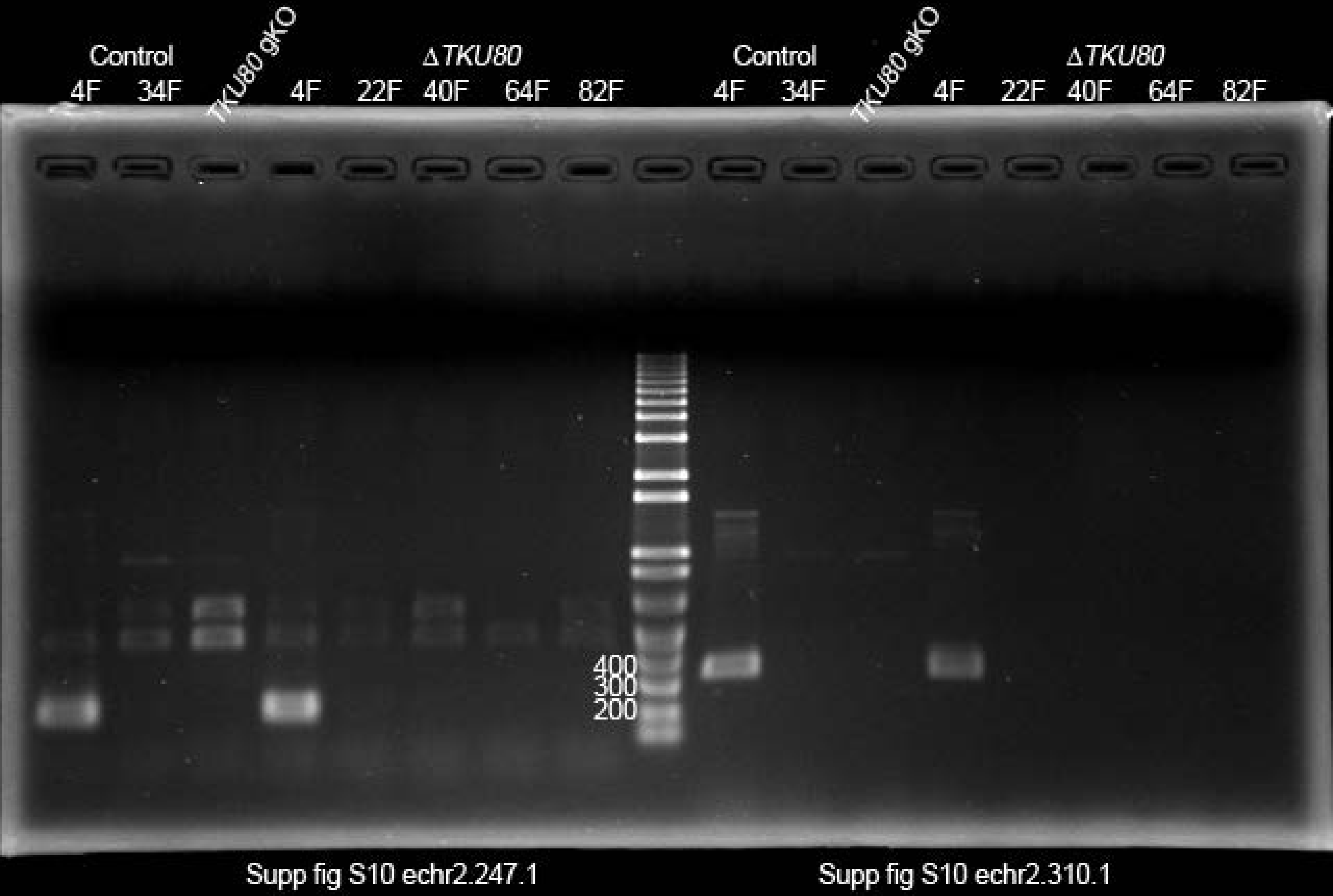
Control
TKU80 gKO
ΔTKU80
4F 34F 4F 22F 40F 64F 82F
Control
TKU80 gKO
ΔTKU80
4F 34F 4F 22F 40F 64F 82F
400
300
200
Supp fig S10 echr2.247.1
Supp fig S10 echr2.310.1

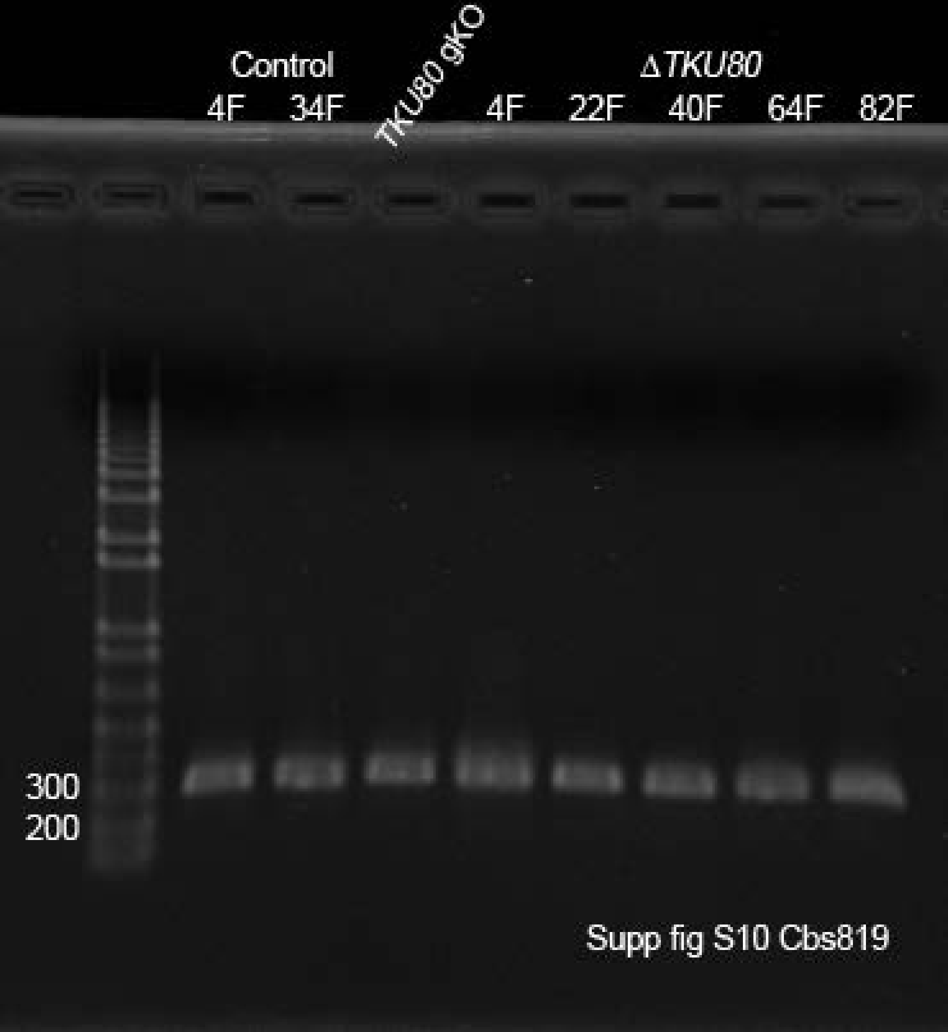
Control
4F
34F
TKU80 gKO
ΔTKU80
4F
22F
40F
64F
82F
300
200
Supp fig S10 Cbs819
